# An integrated respiratory microbial gene catalogue to better understand the microbial aetiology of *Mycoplasma pneumoniae* pneumonia

**DOI:** 10.1093/gigascience/giz093

**Published:** 2019-07-31

**Authors:** Wenkui Dai, Heping Wang, Qian Zhou, Dongfang Li, Xin Feng, Zhenyu Yang, Wenjian Wang, Chuangzhao Qiu, Zhiwei Lu, Ximing Xu, Mengxuan Lyu, Gan Xie, Yinhu Li, Yanmin Bao, Yanhong Liu, Kunling Shen, Kaihu Yao, Xikang Feng, Yonghong Yang, Ke Zhou, Shuaicheng Li, Yuejie Zheng

**Affiliations:** 1 Department of Respiratory Diseases, Shenzhen Children’s Hospital, Shenzhen 518026, China; 2 Department of Computer Science, City University of Hong Kong, Hong Kong 999077, China; 3 Wuhan National Laboratory for Optoelectronics, Huazhong University of Science and Technology, No. 1037 Luoyu Road, Wuhan 430074, China; 4 Department of Microbial Research, WeHealthGene Institute, Shenzhen 518000, China; 5 Institute of Statistics, Nankai University, No. 94 Weijin Road, Tianjin 300071, China; 6 Department of Respiratory Diseases, Beijing Children's Hospital, Beijing 100045, China

**Keywords:** pneumonia, *Mycoplasma pneumoniae*, oropharynx, microbiome, respiratory microbial gene catalogue

## Abstract

**Background:**

The imbalanced respiratory microbiota observed in pneumonia causes high morbidity and mortality in childhood. Respiratory metagenomic analysis demands a comprehensive microbial gene catalogue, which will significantly advance our understanding of host–microorganism interactions.

**Results:**

We collected 334 respiratory microbial samples from 171 healthy children and 76 children with pneumonia. The respiratory microbial gene catalogue we established comprised 2.25 million non-redundant microbial genes, covering 90.52% of prevalent genes. The major oropharyngeal microbial species found in healthy children were *Prevotella* and *Streptococcus*. In children with *Mycoplasma pneumoniae* pneumonia (MPP), oropharyngeal microbial diversity and associated gene numbers decreased compared with those of healthy children. The concurrence network of oropharyngeal microorganisms in patients predominantly featured *Staphylococcus spp*. and *M. pneumoniae*. Functional orthologues, which are associated with the metabolism of various lipids, membrane transport, and signal transduction, accumulated in the oropharyngeal microbiome of children with pneumonia. Several antibiotic resistance genes and virulence factor genes were identified in the genomes of *M. pneumoniae* and 13 other microorganisms reconstructed via metagenomic data. Although the common macrolide/β-lactam resistance genes were not identified in the assembled *M. pneumoniae* genome, a single-nucleotide polymorphism (A2063G) related to macrolide resistance was identified in a 23S ribosomal RNA gene.

**Conclusions:**

The results of this study will facilitate exploration of unknown microbial components and host–microorganism interactions in studies of the respiratory microbiome. They will also yield further insights into the microbial aetiology of MPP.

## Background

Several microorganisms have been identified as indispensible for the respiratory microbiota (RM) [1–5]. In cases of pneumonia, an imbalance in the levels of the microorganisms within the RM [6, 7] is a leading cause of high morbidity and mortality [8, 9] worldwide, especially in children <5 years old [10, 11]. In recent years, the number of cases of *Mycoplasma pneumoniae* pneumonia (MPP) in Chinese children has been increasing [12] and the microbial aetiology of this disease remains poorly understood.

Previous studies by our group have revealed altered RM in children with MPP [13, 14]. However, current RM research has mainly focused on analysis of 16S rRNA [6, 7, 15, 16], which only provides cues about known bacterial components at the genus level. Emerging 16S rRNA analysis has revealed an imbalanced microbial structure in the respiratory tracts of children with pneumonia [7, 17, 18]; however, changes in microbial functions and the species-level microbial components of the RM of patients with MPP remain unexplored. In addition, current multi-omics studies are limited to explorations of known bacterial genomes in the RM [15]. Nevertheless, the RM includes many unknown microbial species [1–3, 5, 6] that require further exploration.

A comprehensive catalogue of reference genes is crucial for in-depth functional metagenomic analysis such as species/gene profiling, discovery of microbial biomarkers, and functional annotation. Given that the RM varies with the environment [19], age [1, 2, 4], and disease [6, 7, 15, 16], we took samples from the nasopharynx (NP), oropharynx (OP), and lungs of 76 children with pneumonia and OP samples from 171 healthy children in China. These samples were used to establish an integrated respiratory microbial gene catalogue (RMGC) with which to study the imbalanced RM in Chinese children with MPP. Using this catalogue, we assessed the microbial components and functions in the OP microbiota of healthy children and children with MPP, as well as the characteristics of recovered microbial genomes.

## Data Description

Between 3 July 2016 and 27 August 2016, 247 patients were recruited from the hospitalization zone in the Department of Respiratory Diseases of Shenzhen Children's Hospital, China. Inclusion criteria were characteristic chest radiographic abnormalities consistent with pneumonia, exclusion of asthma, and clearance of respiratory infections or exposure to antibiotics within 1 month of sampling (Table 1). NP (25–800-A-50, Puritan, Guilford, ME, USA) and OP (155C, COPAN, Murrieta, CA, USA) swabs were collected from 76 hospitalized patients within 24 hours of hospitalization and before the administration of antibiotics. Broncho-alveolar lavage fluid was collected 2–15 days after hospitalization (Supplementary Table 1).

**Table 1:** Sample information

Characteristic	Patients with pneumonia (n = 76)	Healthy children (n = 171)
Sex		
Female	24	87
Male	52	84
Age (years).	2.9 (0.2–12.7)	4.3 (0.1–8.9)
Sampling site		
OP	75	171
NP	42	0
Lung	46	0
Delivery mode		
Vaginal birth	46	102
Caesarean delivery	30	69
Feeding pattern		
Breast	48	84
Breast + formula	12	66
Formula	16	21
Family history of allergy	1	0
History of pneumonia	14	0
Asthma	0	0
Clinical symptoms		
Lung consolidation, atelectasis, infiltration	76	NA
Fever	44	0
Cough	72	0
Wheezing	20	0
Hospitalization time (days)	9 (2–37)	0
C-response protein <0.499 mg/L	22	NA
Procalcitonin <0.5 ng/mL	73	NA
Eosinophils 0.5–5%	44	NA

NA, not available.

Healthy children were recruited during physical examinations at Shenzhen Children's Hospital, China, between July and August of 2016. OP swabs were collected from 171 healthy children meeting the following inclusion criteria: no diagnosis of asthma or family history of allergy; no history of pneumonia; a lack of wheezing, fever, cough, or other respiratory/allergic symptoms at sampling 1 month prior to the study and 1 week after sampling; no exposure to antibiotics 1 month prior to sampling.

All samples were collected by an experienced clinician. Samples were stored at −80°C within 20 minutes of collection and DNA was extracted within 10 days of sampling. A TGuide S32 Magnetic Swab Genomic DNA Kit (DP603-T2, TIANGEN Biotech [Beijing] Co., Ltd., Beijing, China) was used to extract the DNA. Metagenomic sequencing was performed using the Illumina Hi-Seq platform (San Diego, CA, USA) according to the manufacturer's instructions. Unused swabs and DNA extraction kits from the same batch served as negative controls to assess DNA contamination.

### Sample information and data output

Two hundred forty-seven children aged <13 years were enrolled in this study (Table 1 and Supplementary Table 1). After removal of host contamination and low-quality data, metagenomic sequencing produced 4,765,288,986 reads, with a mean of 14,267,332 reads per sample. The DNA concentration of unused sampling swabs and DNA extraction kits was <0.01 ng/μL, whereas the DNA concentration was >80 ng/μL in sampling swabs and broncho-alveolar lavage fluid. Furthermore, the DNA amplification results of extracted bacterial DNA were <0.01 nmol/L for the enveloped sampling or extraction materials (Supplementary Fig. 1)

### Construction of the RMGC

**Figure 1:**
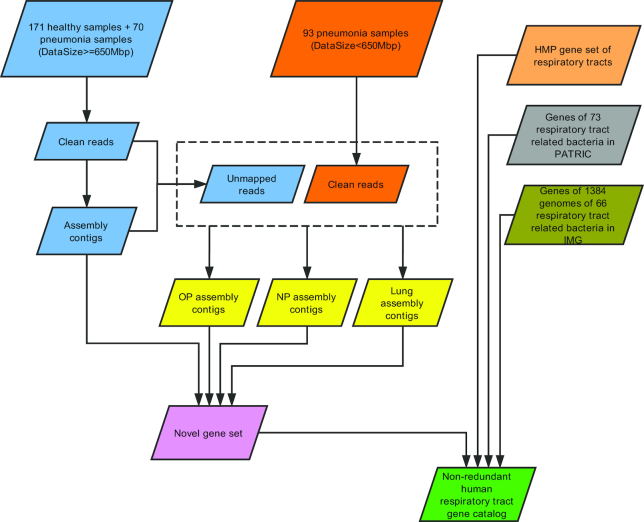
Construction of the human Respiratory Microbial Gene Catalogue (RMGC). Genome assembly was performed for each sample with ≥650 Mb of data. For samples with <650 Mb of data, data from the same respiratory site (NP, OP, or lung) were mixed and assembled. Gene predictions were conducted for all assembled contigs with ≥500 bp and respiratory bacterial genomes in the Integrated Microbial Genomes and Microbiomes (IMG) database. Genes with ≥100 bp were retained. Respiratory gene sets from the Human Microbiome Project (HMP) and Pathosystems Resource Integration Center (PATRIC) were combined to construct the non-redundant RMGC containing 2,245,343 genes.

**Figure 2:**
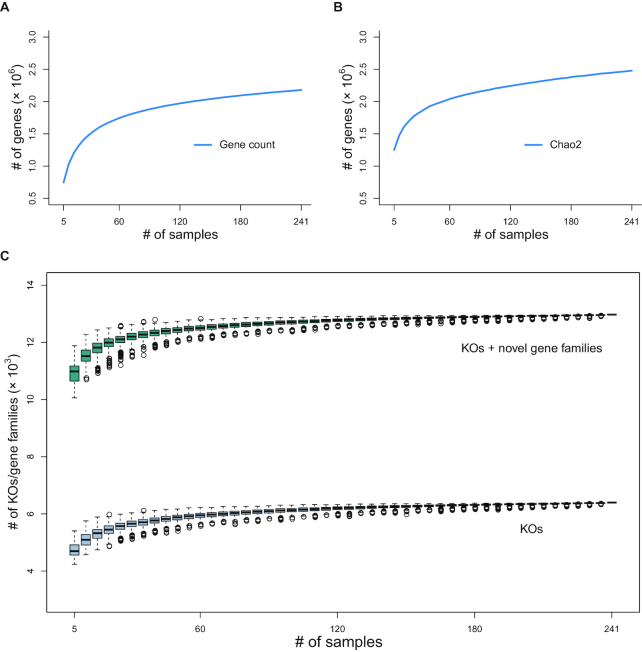
Rarefaction curves for genes and KEGG orthologous groups/gene families. **(a)** Rarefaction curve for gene count. **(b)** Rarefaction curve for Chao2. The Respiratory Microbial Gene Catalogue (RMGC) captured 90.52% of the prevalent genes. **(c)** Rarefaction curve for KEGG orthologous groups/gene families. Known functions saturate quickly to 6,346 groups. After including novel gene families, the rarefaction curve plateaus when 12,924 groups are detected. Boxes represent the interquartile ranges (IQRs) between the first and third quartiles, and the line inside the box represents the median value. Whiskers represent the lowest or highest values within values 1.5 times the IQR from the first or third quartiles. Circles represent outliers.

By applying metagenomics sequencing data from 247 children and 3 respiratory-related microbial genome/gene resources (Fig. 1), we constructed a comprehensive RMGC with 2,245,343 non-redundant open reading frames (ORFs). Data are freely accessible on our website [20]. The total length of ORFs in the RMGC was 1.71 Gb and the mean length was 760 nucleotides (nt) (range: 102–32,241 nt). We selected 241 samples with ≥650 Mb data to examine the coverage of the microbial genes in the RMGC. In accordance with the rarefaction curve, 90.52% of prevalent microbial genes were captured in the RMGC (Fig. 2a and b).

### Taxonomic assessment and functional annotation of the RMGC

Based on taxonomic profiling, 1,281,673 genes (57.08% of RMGC) were assigned to phyla and 1,143,382 genes (50.92% of RMGC) were assigned to genera, representing 56.58% and 51.75% of the sequencing reads, respectively. A total of 617,968 genes (25.92% of RMGC) were annotated to known bacterial species, representing 33.49% of the sequencing reads. The RMGC was dominated by the phyla Firmicutes, Bacteroides, Proteobacteria, Actinobacteria, and Fusobacteria, while the most prevalent microbial genera were *Staphylococcus, Streptococcus, Haemophilus, Corynebacterium, Dolosigranulum, Prevotella, Blautia, Rothia, Porphyromonas, Lactobacillus, Veillonella, Fusobacterium*, and *Leptotrichia*. Unknown microbial species accounted for 9.62–55.50% of the RMGC. Detailed taxonomic information of the RMGC is deposited on our website [20].

Metagenomic analysis revealed a genus-level microbial structure resembling the results of the 16S rRNA analysis (Supplementary Fig. 2). Notably, a greater proportion of microbial genera remained unclassified in the metagenomic analysis than in the 16S rRNA analysis; this might be attributed to the wide detection by metagenomics sequencing and limited reference microbial genomes.

By aligning the RMGC to the KEGG database, 6,408 KEGG KO groups were identified, including 853,446 genes representing 37.85% of the total sequencing data. As more samples were included, known microbial functions (annotated by KEGG) saturated quickly to 6,346 groups (Fig. 2c). Combining novel gene families, the rarefaction curve plateaued when 12,924 groups were detected (Fig. 2c). Upon alignment to the eggNOG database, 53.95% of genes in the RMGC were assigned to known functional categories.

### Core microbial species in the OP microbiota of healthy children

**Figure 3:**
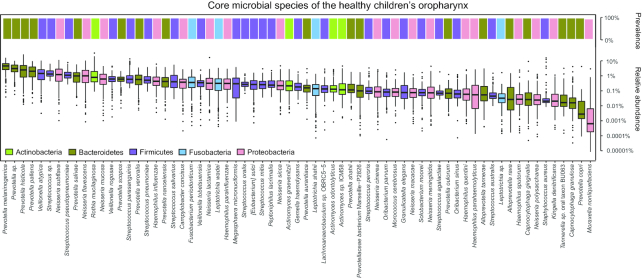
Core microbial species in healthy OP microbiota. Upper bar plot represents the prevalence of each core species; lower box plot shows the relative abundance of each core species. Different colours refer to different phyla. Boxes represent the interquartile ranges (IQRs) between the first and third quartiles, and the line inside the box represents the median value. Whiskers represent the lowest or highest values within values 1.5 times the IQR from the first or third quartiles. Dots represent outliers.

We acquired 67 core species across 5 dominant phyla: Bacteroidetes, Firmicutes, Proteobacteria, Actinobacteria, and Fusobacteria (Fig. 3). *Prevotella melaninogenica* (4.38 ± 2.91%, mean ± SD), *Prevotella sp*. (3.06 ± 1.92%), *Prevotella histicola* (3.23 ± 3.58%), *Prevotella pallens* (2.31 ± 1.88%), and *Veillonella atypical* (1.60 ± 1.44%) were the top 5 microbial species. In addition, *Streptococcus pseudopneumoniae* (1.26 ± 0.96%), *Haemophilus influenzae* (0.60 ± 0.68%), *Streptococcus pneumoniae* (0.60 ± 0.50%), *Haemophilus parainfluenzae* (0.42 ± 0.49%), and *Staphylococcus aureus* (0.27 ± 1.52%), which were generally defined as opportunistic pathogens, were also prevalent in the OP microbiota of healthy children (Fig. 3).

### Microbial structure and functions of the OP microbiome of children with MPP differed from those of healthy children

**Figure 4:**
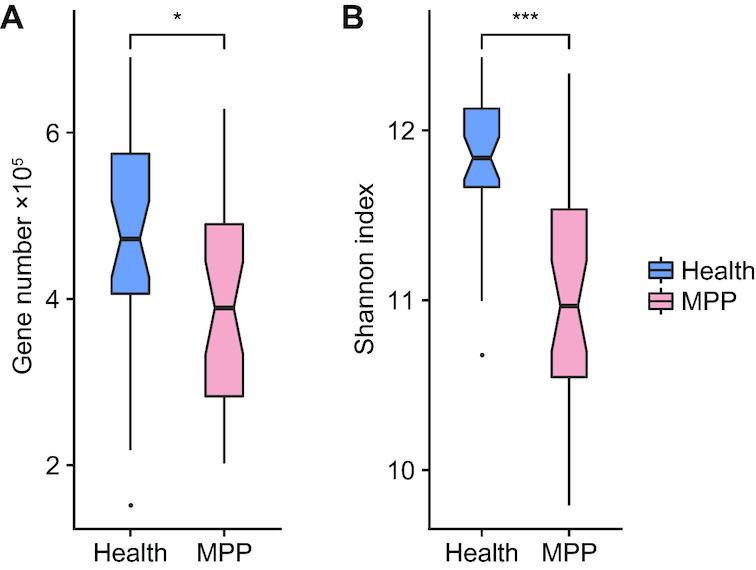
Differentiation of oropharyngeal (OP) microbial samples between healthy children and children with *Mycoplasma pneumoniae* pneumonia (MPP). **(a)** Gene counts in the OP microbiotas of healthy children and children MPP. (**b)** α Diversity of the OP microbiota in healthy children and children with MPP. Boxes represent the interquartile ranges (IQRs) between the first and third quartiles, and the line inside the box represents the median. Whiskers represent the lowest or highest values within values 1.5 times the IQR from the first or third quartiles. Dots represent outliers. *, *** represents *P* ≤ 0.05, ≤ 0.001.

**Figure 5:**
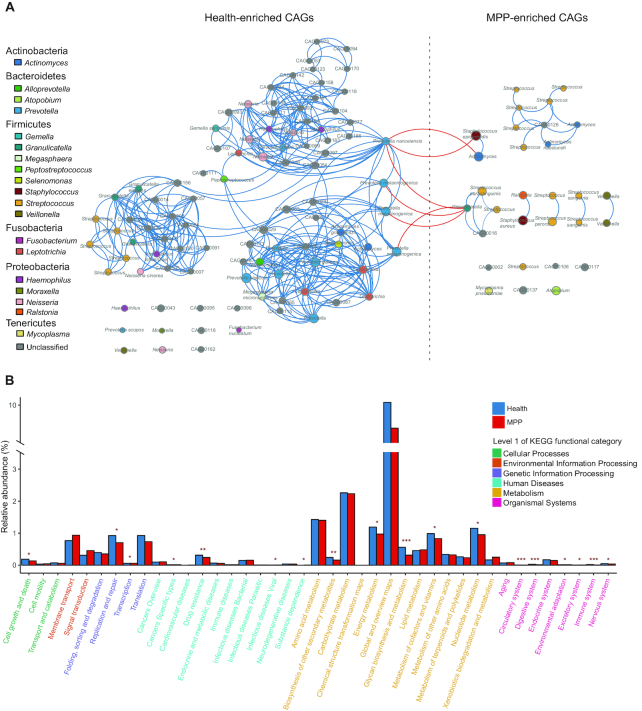
Phylogenetic and functional alterations in children with pneumonia. **(a)** Size of the circle represents the mean relative abundance of co-abundance gene groups (CAGs) in healthy children or children with *Mycoplasma pneumoniae* pneumonia (MPP). A line between 2 circles indicates a Spearman's rank correlation coefficient ≥0.6 and an adjusted *P*-value ≤0.05. The phylum and genus corresponding to each CAG are indicated in the key. **(b)** X-axis represents level 2 functional categories in the KEGG; colours of the characters represent level 1 functional categories (listed on the right); Y-axis shows the relative abundance of level 2 functional categories. Adjusted *P*-values at *, ≤0.05; **, ≤0.01; and ***, ≤0.001, respectively.

Based on permutational ANOVA, onset of pneumonia is the most significant factor (adjusted *P*-value <0.001) explaining the variations observed in OP microbial samples. This is followed by feeding pattern (adjusted *P*-value = 0.037) and age (adjusted *P*-value = 0.048). Compared with healthy children, children with MPP exhibited significantly decreased microbial gene numbers and α diversity of the OP microbiota (Fig. 4a and b). Thirty co-abundance gene groups (CAGs) accumulated significantly in the OP microbiota of children with MPP, comprising 6 unknown and 24 known microbial species. These species were primary respiratory pathogens such as *M. pneumoniae, Staphylococcus epidermidis*, and *S. aureus* (Fig. 5a). Ninety-five CAGs were enriched in the OP microbiota of healthy children, including prevalent colonizers such as *Prevotella* species (Fig. 5a). The microbial co-occurrence networks of children with MPP were simpler than those of healthy children; negative correlations were only identified between healthy-enriched and MPP-enriched CAGs (Fig. 5a). For example, healthy-enriched *Prevotella spp*. negatively correlated with MPP-enriched *S. epidermidis* (*r* < –0.60, adjusted *P*-value ≤0.05, Fig. 5a).

By comparing functional KEGG annotations (Supplementary Table 2), we assessed the functional alterations of the OP microbiota in children with MPP. Microbial functions relating to lipid metabolism, membrane transport, and signal transduction were slightly enriched in children with MPP (Fig. 5b). In contrast, the OP microbiota of healthy children was significantly enriched in orthologues involved in glycan biosynthesis and metabolism, biosynthesis of secondary metabolites, and cell growth and death (Fig. 5b and Supplementary Table 2). Host homeostasis-associated functions, such as the immune system, digestive system, circulatory system, and environmental adaptation, were also significantly abundant in the OP microbiota of healthy children (Fig. 5b and Supplementary Table 2).

### Characterization of the *M. pneumoniae* genome and 13 other reconstructed microbial genomes

**Figure 6:**
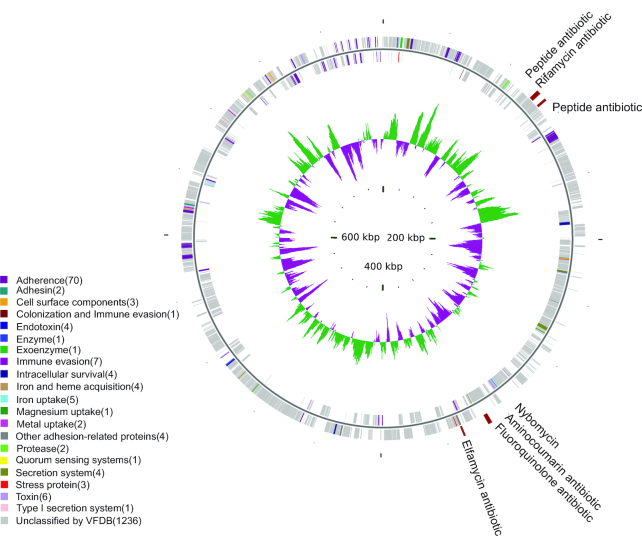
Virulence factor genes (VFGs) and antibiotic resistance genes (ARGs) on the *Mycoplasma pneumoniae* genome. Tracks from outside to inside represent ARGs, genes on the plus strand, genes on the negative strand, and guanine-cytosine skew, respectively. VFG colours indicate VFG type.

We re-assembled 14 qualified microbial CAGs (Supplementary Table 3), representing the *M. pneumoniae* genome (0.80 Mb) and the genomes of 13 other microorganisms (mean genome size: 2.30 Mb). The *M. pneumoniae* genome accumulated significantly in the OP microbiota of MPP patients and had high similarity with the reference genome (97.79% genome coverage; Supplementary Table 3). The *M. pneumoniae* genome included 4 antibiotic resistance genes (ARGs) against common antibiotics including peptide, rifamycin, and fluoroquinolone (Fig. 6, Supplementary Table 4). In 8 patients who had been given experimental macrolides or β-lactams (such as azithromycin, erythromycin, or sulbactam), a single-nucleotide polymorphism (SNP) mutation, A2063G, which is related to macrolide resistance, was identified in the 23S rRNA (Supplementary Table 1). In addition, 136 virulence factor genes (VFGs) were found along its reassembled genome sequence (Supplementary Table 5), as well as redundant *M. pneumoniae* VFGs enriched in the secretion of adhesin P1, cytadherence protein, and community-acquired respiratory distress syndrome (CARDS) toxin (Fig. 6 and Supplementary Table 5).

**Figure 7:**
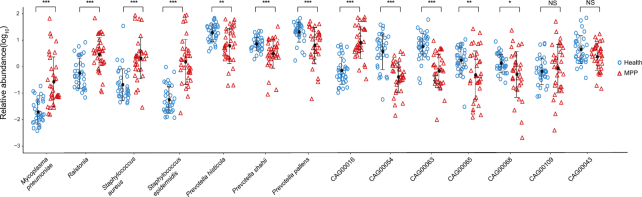
Comparison of relative abundance of 14 re-assembled genomes between healthy children and children with *Mycoplasma pneumoniae* pneumonia. Blue circles and red triangles represent the microbial relative abundance of healthy children and children with *Mycoplasma pneumoniae* pneumonia (MPP). Solid dot and paired whiskers represent the mean and SD of each microbial relative abundance. *P*-values at *, ≤0.05; **, ≤0.01; and ***, ≤0.001, respectively. NS, not significant.

Of the 13 other microbial genomes included in our study, 5 could be annotated as specific species, 1 was annotated at the genus level (*Ralstonia*), and the remaining 7 were novel microbial genomes (mean genome size 1.74 Mb) (Supplementary Table 3). For the 5 annotated microbial species, *S. aureus* and *S. epidermidis* increased significantly in MPP patients, while the other 3 *Prevotella spp*. mainly accumulated in healthy children (Fig. 7, Supplementary Table 3). The largest reassembled *Ralstonia* genome (5.89 Mb) carried numerous ARGs, including 13 β-lactam ARGs, 21 tetracycline ARGs, and 11 macrolide ARGs. *P. histicola, Prevotella shahii*, and CAG00068 all had 1 copy each of a macrolide resistance gene and a β-lactam ARG. These genomes also harboured abundant VFG resources, ranging from 105 to 808 copies of relative genes. Correlation analysis revealed no significant correlation between these 14 reassembled microbial genomes and 5 clinical indices (Supplementary Table 6).

## Discussion

Morbidity and mortality related to MPP is increasing in Chinese children. The development of RM studies has improved our understanding of the microbial aetiology of MPP by revealing infection-associated RM imbalances [13, 14]. However, microbial functions and host–microbiota interactions in the RM of patients with MPP remain to be explored, particularly for novel microbial species.

In recent years, several reference gut microbial catalogues have been constructed to promote understanding of host–microorganism interactions. Qin et al. built a global view of the human gut microbiome (GM) and revealed a comprehensive functional potential of the prevalent gut microbial genes [21]. Li et al. upgraded the gut gene catalogue in 2014 [22], enabling studies to associate microbial genes with human health conditions. These frameworks have allowed researchers to deepen their understanding of the correlations between GM and various diseases, such as gastrointestinal and cardiovascular diseases [23, 24].

Like the reference gene catalogues that have been developed for the GM, our RMGC will further understanding of microbial aetiology in respiratory diseases. The development of a well-established RMGC is crucial for the functional metagenomics analysis needed to improve our knowledge of host–microbiota interactions in MPP. By aligning metagenomics data directly with the RMGC we have established, researchers will be able to profile all microbial species, as well as explore microbial functions in both known and unknown microbial species. The fact that microbial assignment using the RMGC proved similar to that by 16S rRNA analysis also suggests that the RMGC may be promising for taxonomic studies using our constructed gene sets. Our data on the core microbial species of the OP microbiota in healthy children will provide a reference for exploring microbial and host–microorganism interactions in RM studies [25]. More generally, the RMGC provides a comprehensive respiratory-associated microbial profile to further microbiota analysis at the species level; functional profiling will facilitate further, more in-depth multi-omics analyses [26, 27], such as associations between protein products and metabolites with known and novel microbial genomes. This capability would help to clarify interactions between the host and alterations to the RM during the progression of disease in MPP.

The OP microbiota of children with MPP has a simpler structure than that of healthy children. Previous studies have revealed that the bacteria-like *M. pneumoniae* is able to deplete levels of other bacteria through direct competition, and activates bacterial clearance factors in host responses [28, 29]. This leads to decreased numbers of colonizing *Prevotella spp*. [30] and reduced proliferation of pathogens such as *S. aureus* and *S. epidermidis*. MPP patient–enriched genes have functions involved in membrane transport and the metabolism of various nutrients, which may partly explain the reduced tight junction proteins and increased respiratory mucosa permeability after infection [31]. Several studies have also identified increased glucose concentration in airway surface liquids [32–34] and associated pathogen proliferation [35]. This also corroborates the existence of enriched nutrient uptake pathways in the OP microbiota of children with MPP. Although the mechanism of *M. pneumoniae* clearance in the respiratory system remains unclear, these findings may provide new insights into host–microbiota interactions in MPP infection.

As well as a variety of well-known microorganisms, respiratory tracts also harbour numerous undiscovered microbial species [36]. Moreover, recent reports have demonstrated that a single bacterial genome can be recovered via reference gene sets and metagenomics data [37, 38]. It is difficult to culture *M. pneumoniae*; thus, it is rarely done in clinical diagnosis, and this limits our understanding of antibiotic resistance and virulence [39] in this species. Reconstruction of the *M. pneumoniae* genome using the RMGC and metagenomics data has indicated the existence of various ARGs related to RNA transcription [40], DNA replication [41], and protein synthesis [42]. According to clinical practice guidelines [43–45], most children with MPP are treated with azithromycin, erythromycin, or sulbactam; none of these were associated with ARGs identified in the *M. pneumoniae* genome. Increasingly, reports demonstrate that specific dominant bacteria are associated with severe acute respiratory infections [6, 15, 46], but there have been no meaningful correlations identified between disease severity and *M. pneumonia*—or indeed between the other species of reassembled bacteria found in the OP microbiota of children with MPP. In our previous studies, we confirmed the succession of *M. pneumoniae* infection in NP to OP and lung, and determined an association between *M. pneumoniae* load in the lung microbiota with disease severity [14].

Although no macrolide/β-lactam resistance genes were discovered in the *M. pneumoniae* genome, 1 SNP mutation (23S RNA, 2063A→G) associated with macrolide resistance was identified in children with MPP. Meanwhile, in patient-enriched microbial genomes such as *Ralstonia*, plenty of ARGs related to macrolide, β-lactam, and tetracycline resistance were found. Given rigorous antibiotic selective pressure and complex microbial interactions, environmentally redundant genetic components can be rapidly transferred into the pathogen genome by horizontal gene transfer [47, 48], causing the emergence of several diseases, such as happened in the European enterohemorrhagic *Escherichia coli* breakout [49] and the emergence of scarlet fever caused by *Streptococcus pyogenes* in Hong Kong [50]. In consideration of these findings, it should be recognized that current regimens for the treatment of *M. pneumoniae* hold the potential to trigger emerging drug-resistant strains, whether in *M. pneumoniae* or other novel microbial species. Indeed, macrolide resistance has already been reported in *M. pneumoniae*-PCR-positive children [51–53].

In OP microbiota samples from healthy children, several bacterial genomes were recovered, including the key player *Prevotella spp*. [54, 55], and several other novel microorganisms, such as *Vampirovibrio*, which might function as pathogen competitors [56]. Microbial genomes recovered from respiratory tracts hold the potential to improve our understanding of the microbial aetiology in MPP pneumonia.

There are several limitations of this study. Given that there are currently no effective medicines for MPP, the patients in our study received empirical treatments that might have slightly shifted their airway ecology [57]. Despite promising applications for the RMGC, unclassified CAGs and novel gene families in the RMGC must be annotated and further explored. The copy numbers of several genes require further assessment because of the potential for inaccuracies caused by the low respiratory bacterial biomass, next-generation sequencing, and assembly methods. In the present study, the respiratory microbial samples were obtained from Chinese children; therefore, like the continual updates made to the GM reference genes, metagenomics data from a more diverse sample will be incorporated into the RMGC in the future to more broadly characterize microbial components and functions [21, 22, 58]. This will incrementally improve studies of the imbalanced RM in patients with respiratory diseases. Alterations in the OP microbiota of Chinese patients with MPP will also provide extensive insights into the microbial aetiology of acute respiratory infection.

## Potential Implications

The RMGC established here will help to deepen respiratory micro-ecology research and has the potential to elucidate respiratory microbial communities at the microbial species level. In addition, by aligning metagenomics data with the reference catalogue, our work will facilitate assembly of the genomes of novel microbial genera or species, allow exploration of microbial functions and their associated microbial components, and allow the construction of whole microbial networks within the respiratory microbial community. Established reference gene sets can be used to deepen multi-omics analysis, which will further our understanding of host–microorganism interactions in acute respiratory infection. One example of how these gene sets might be used is in the comparison of the OP microbiota of healthy and diseased children.

## Methods

### Clinical detection of infectious pathogens

Broncho-alveolar lavage fluid was used to identify common microorganisms of the microbiota. Culturing was conducted to detect *Streptococcus pneumoniae, H. influenzae, Moraxella catarrhalis, S. aureus*, and *Staphylococcus haemolyticus*. The D3 Ultra DFA Respiratory Virus Screening and ID Kit (Diagnostic Hybrids, Inc., Athens, OH, USA) was used to detect common viruses, including adenovirus, respiratory syncytial virus, influenza virus, and parainfluenza virus. Cytomegalovirus (CMV) and Epstein-Barr virus (EBV) were detected using the Diagnostic Kit for Quantification of Human CMV DNA and EBV PCR Fluorescence Quantitative Diagnostic Kit, respectively (DaAnGene, Guangzhou, China). *M. pneumoniae* and *Chlamydia pneumonia* were diagnosed using a diagnostic kit for *M. pneumoniae* DNA (PCR Fluorescence Probing; DaAnGene) and Anti *C. pneumoniae* ELISA (IgM) (EUROIMMUN AG, Lübeck, Germany), respectively.

### Construction and annotation of the RMGC

Sequencing data were filtered using a previously reported method [59]. Samples with ≥650 Mb data (Fig. 1, Supplementary Fig. 3) were selected for genome assembly using SOAPdenovo [60] (SOAPdenovo, RRID:SCR_010752, v2.07, –F –K 39 –M 3 –d 1). For samples with <650 Mb data, data from the same respiratory site were mixed and assembled (Fig. 1). Assembled contigs with ≥500 bp were selected for gene prediction with MetaGeneMark [61] (v3.26, default parameters). We applied Glimmer3.02 [62] (Glimmer, RRID:SCR_011931, default parameters) to predict genes from the 1,384 respiratory bacterial genomes in the Integrated Microbial Genomes and Microbiomes (IMG) database (21 December 2016 [63]). Gene sequences were also retrieved from the genomes of 73 respiratory bacteria in the Pathosystems Resource Integration Center (PATRIC) database (25 March 2017 [64]) and 450,204 ORFs of respiratory bacteria in the Human Microbiome Project (HMP) (20 October 2016 [65]). Genes with a length ≥100 bp and without Ns (unidentified nucleotides) were selected to construct non-redundant gene sets using CD-HIT [66] (CD-HIT, RRID:SCR_007105, v4.66, –c 0.95 –aS 0.9). Genes with ≥2 mapped reads were retained in the established RMGC.

Taxonomic annotation of genes was conducted as follows:

- Bacterial and viral genome sequences were retrieved from IMG (21 December 2016), PATRIC (25 March 2017), and NCBI NT databases (9 August 2016 [67]). The genome sequence with the longest N50 was selected as the representative genome for each bacterial species. Non-redundant viral genomes were produced by CD-HIT (v4.66, -aS 0.95 -aL 0.9 -M 0). Gene sets in the RMGC were aligned to 6,869 representative bacterial genomes and 18,916 non-redundant viral DNA genomes using BLASTN (BLASTN, RRID:SCR_001598, v2.5.0, default parameters except –e 0.01);
- The top 10% highest scoring alignments of each gene were retained, with ≥65% identity and ≥80% gene length coverage;
- Assignment of each gene was determined on the basis of ≥50% consensus above the similarity threshold for a specific rank: ≥65% for phylum, ≥85% for genus, and ≥95% for species.

The functional annotation of each gene was determined by searching protein sequences in the KEGG (v78.1) database and eggNOG (version 4.0) with BLASTP (BLASTP, RRID:SCR_001010, v2.5.0, default parameters, except -e 1e-5). The best-hit alignment (identity ≥30% and coverage ≥70%) was selected as the functional annotation for the gene. Genes without annotations in KEGG were identified as novel gene families by the Markov cluster algorithm [68] (inflation factor = 1.1, bit-score cut-off = 60).

### Comparing taxonomic assessments of 16S rRNA gene analysis and metagenomic analysis

Seventy-two OP microbial samples with ≥650 Mb metagenomic sequencing data were aligned to establish the RMGC to determine taxonomic assignments. The same samples were also sequenced via the V3–V4 region of the 16S rRNA gene [13]. Microbial compositions obtained by these 2 methods were compared to assess the accuracy of taxonomic assignments via metagenomic analysis.

### Rarefaction analysis

We downsized the number of mapped reads to 3 million per sample to eliminate variable influence caused by the quantity of sequencing data. Total gene richness was estimated by randomly sampling 5 individuals 1,000 times using gene counting and the Chao2 richness estimator [69].

To produce rarefaction curves of KEGG orthologous groups (KOs) and novel gene families, saturation was evaluated by randomly sampling 5 individuals 1,000 times. Relative rarefaction curves were visualized using R software (v3.3.2).

### Calculation of gene relative abundance in RMGC

Filtered reads of metagenomics data from each sample were aligned to the RMGC using BWA (BWA, RRID:SCR_010910, v0.7.13, default parameters, except for the mem and identity ≥95%). Alignments meeting the following 2 criteria were accepted: (i) paired-end reads mapped onto the same gene with the correct insert size; and (ii) 1 end of a paired-end read was mapped onto the end of a gene, while the other was located outside of the gene.

If the number of genes in a given sample was *n*, the relative abundance was calculated using the following steps:

1. The copy number of the gene *i* (*c*(*i*)) was calculated as:
  \begin{equation*}
c(i) = \frac{{t(i)}}{{l(i)}},
\end{equation*}
  where *t*(*i*) is the total number of mapped reads of gene *i* in a given sample, and *l*(*i*) is the length of the gene *i*.
2. The relative abundance of gene *i*(Ab*_g*(*i*)) was defined as:
  \begin{equation*}
\mathrm{Ab}\_g(i) = \frac{{c(i)}}{{\sum\nolimits_1^n {c(i)} }}.
\end{equation*}
3. If *m* genes can be assigned to the phylogenetic assignment *s*, the abundance of this phylogenetic assignment (Ab*_p*(*s*)) was calculated using the following equation:
  \begin{equation*}
\mathrm{Ab}\_p(s) = \sum\nolimits_1^m {\mathrm{Ab}\_g(j)}.
\end{equation*}

### Phylogenetic and functional profile of the OP microbiome

All filtered reads of the OP microbiota were aligned to the RMGC using BWA with same parameters as described above. The relative abundance of each phylogenetic assignment was calculated as shown above, while the abundance of KOs in the functional profiling table was determined as described in a previous report [21].

### Identification of core OP microbial species in healthy children

Microbial species were selected as “core” species if they existed in >50% of healthy children and had >1% relative abundance in 1 OP microbial sample. Distributions of core microbial species in the OP of healthy children were described using ggplot2 in R.

### Comparison of the OP microbiota between healthy children and children with MPP

Thirty-three healthy children were chosen, with a similar age distribution to that of the 34 children with MPP (data size ≥650 Mb). Genes in the OP microbiota of selected microbial samples were clustered into CAGs via Capony-based algorithms [70] (default parameters). Selected CAGs containing >700 genes were regarded as being derived from the same bacterial genome and were selected to construct a correlation network using Spearman's rank coefficient (≤–0.6 or ≥0.6). The co-occurrence network was visualized using Cytoscape (v3.4.0) [71]. If ≥50% of the included genes had consensus phylogenetic annotations, the corresponding CAG was assigned to a related microbial taxonomic assignment.

The relative abundance of each CAG in our microbial samples was calculated as previously reported [58]. Intergroup comparisons between CAGs and KEGG functions were performed using the 2-tailed Wilcoxon rank-sum test and corrected via the Benjamini-Hochberg method (adjusted *P*-value ≤0.05). Confounding factors including pneumonia, sex, age, delivery mode, and feeding pattern were also assessed using permutational ANOVA by the vegan package (v2.3–4) in R.

### Single microbial genome assembly from OP metagenomic data

OP metagenomic data were aligned to the filtered CAGs (those containing ≥700 genes) by BWA (v0.7.13, identity ≥95%). The mapped reads of each CAG were extracted for microbial genome assembly with Velvet [72] (Velvet, RRID:SCR_010755, kmer: from 45 to 75, cov_cutoff: auto, exp_cov: auto). The assembled sequences with the longest contig N50 were selected as representative draft genomes. Assembly quality was assessed using the following 6 criteria [24]: (i) 90% of the genome assembly included in contigs >500 bp, (ii) 90% of the assembled bases at >5× read coverage, (iii) contig N50 >5 kb, (iv) scaffold N50 >20 kb, (v) mean contig length >5 kb, and (vi) >90% of core genes present in the assembly. Fourteen draft microbial genomes passed 5 or 6 of these criteria (Supplementary Table 3). The assembly quality estimation standard published by the Genomic Standards Consortium (GSC) was then applied (Supplementary Table 3) [73]. The microbial species designation of 14 assembled genome sequences followed these standards: (i) concordance with taxonomical assignment of CAGs [70]; (ii) aligned to the genome sequences published by IMG, NCBI, and PATRIC via BLASTN (v2.5.0, default parameters except –e 0.01), with ≥95% nucleotide identity and ≥95% genome coverage; and (iii) assigned by CheckM (CheckM, RRID:SCR_016646, v1.0.12, default parameters) from the Genome Taxonomy database [74]. Furthermore, gene prediction was executed with Glimmer3.02 (Glimmer, RRID:SCR_011931), while related annotations of antibiotic resistance and virulence genes were acquired through CARD [75] and Virulence Factor Database (VFDB) [76]. The SNP mutation associated with macrolide resistance of *M. pneumoniae* was identified by mapping sequencing reads against 23S rRNA genes [77] using BWA.

### Correlations between reassembled microbial genomes and disease severity in patients with MPP

The correlation between reconstructed microbial genomes and hospitalization duration and fever peak was assessed using R software, a well as serum C-response protein, procalcitonin, and eosinophil levels at 24 hours after hospitalization. The distributions of relative abundance of 14 reassembled genomes in children with MPP and healthy children were showed via scatter plot.

## Availability of supporting data and materials

The BioProject ID for this study is PRJNA413615. The sequencing data supporting the results of this article are available in the GenBank repository under accession number SRP119571. The gene profiles are freely accessible in the RMGC database [20].

All supporting data and materials are available in the *GigaScience* GigaDB database [78].

## Additional files

**Supplementary Figure 1**. DNA gel electrophoresis results. M1: Marker 1 (Trans 2k plus); M2: Marker 2 (Trans 15k plus). The lightest bands are highlighted with red fonts. S: Human DNA, as standard sample. 1, 2, 3: Unused nasopharyngeal swabs; 4, 5, 6: Unused oropharyngeal swabs; 7, 8, 9: Enveloped DNA extraction kits.

**Supplementary Figure 2**. Comparison between taxonomic annotation by aligning metagenomics data with RMGC and 16S rRNA analysis. The number on the left of the horizontal histogram represents sample ID. Upper histograms mean top 20 genera in 16S rRNA analysis results, and lower histograms represent microbial structure based on metagenomics analysis.

**Supplementary Figure 3**. Estimation of sequencing data abundance after filtering human sequence. The curve sharply decreased as the data size became <650 Mb. X-axis means samples sorted by the usable sequencing data with descending order; Y-axis means the data size of sample.

**Supplementary Table 1**. Sample characteristics.

**Supplementary Table 2**. Enriched gene function (based on KEGG) between healthy children and patients with *Mycoplasma pneumoniae* pneumonia.

**Supplementary Table 3**. Evaluation of 14 assembled genomes derived from CAGs.

**Supplementary Table 4**. Antibiotic-resistance genes (ARGs) annotation in 14 re-assembled genomes.

**Supplementary Table 5**. Virulence-factor genes (VFGs) annotation in 14 re-assembled genomes.

**Supplementary Table 6**. Correlation between 14 genomes and clinical characteristics.

giz093_GIGA-D-19-00029_Original_Submission

giz093_GIGA-D-19-00029_Revision_1

giz093_GIGA-D-19-00029_Revision_2

giz093_GIGA-D-19-00029_Revision_3

giz093_Response_to_Reviewer_Comments_Original_Submission

giz093_Response_to_Reviewer_Comments_Revision_1

giz093_Response_to_Reviewer_Comments_Revision_2

giz093_Reviewer_1_Report_Original_Submission Xiao Li -- 4/10/2019 Reviewed

giz093_Reviewer_2_Report_Original_Submission Eduardo Castro -- 4/20/2019 Reviewed

giz093_Supplemental_Files

## Abbreviations

ANOVA: analysis of variance; ARG: antibiotic resistance gene; bp: base pairs; BWA: Burrows-Wheeler Aligner; CAG: co-abundance gene group; CARD: community-acquired respiratory distress syndrome; CMV: cytomegalovirus; EBV: Epstein–Barr virus; ELISA: enzyme-linked immunosorbent assay; Gb: gigabase pairs; GM: gut microbiome; HMP: Human Microbiome Project; IMG: Integrated Microbial Genomes and Microbiomes; IQR: interquartile range; KEGG: Kyoto Encyclopedia of Genes and Genomes; KO: KEGG orthologous group; Mb: megabase pairs; MPP: *Mycoplasma pneumoniae* pneumonia; NCBI: National Center for Biotechnology Information; NP: nasopharynx; nt: nucleotides; OP: oropharynx; ORF: open reading frame; PATRIC: Pathosystems Resource Integration Center; PP: paediatric pneumonia; RM: respiratory microbiome; RMGC: respiratory microbial gene catalogue; SNP: single-nucleotide polymorphism; VFDB: Virulence Factor Database; VFG: virulence factor gene.

## Ethics approval

Ethical approval for this study was obtained from the Ethical Committee of Shenzhen Children's Hospital (Shenzhen, Guangdong Province, China) under registration number 2,016,013. All experiments were performed under the relevant guidelines and regulations. Guardians of all children included in this study provided their informed consent to participate.

## Competing interests

The authors declare that they have no competing interests.

## Funding

This study was supported by grants from the Key Medical Disciplines Building Project of Shenzhen (grant number SZXJ2017005), the Sanming Project of Medicine in Shenzhen (grant number SZSM201512030), the Shenzhen Science and Technology Project (grant numbers JCYJ20170303155012371 and JCYJ20170816170527583), and the Guangdong Medical Research Fund (grant number A2017213).

## Authors' contributions

Y.Z., Y.Y., and K.Z. managed the project. Z.L., G.X., and Y.B. collected samples and information. W.W. and Q.Z. prepared the DNA extraction. D.L., Q.Z., X.F., and Z.Y. performed the bioinformatics analysis. C.Q., Y. Li, and Y. Liu optimized the graphs. X.X. and M.L. optimized the data curation. S.L. and Y.Y. guided data interpretation. X.F. developed the website. H.W. and W.D. mined the data and wrote the manuscript. K.S. and K.Y. polished the manuscript. All authors read and approved the final version of the manuscript.

## Author information

Y.Y. is a Russian academician of paediatric and vaccine research. Y.Z. is Director of the Respiratory Disease Department at Shenzhen Children's Hospital, China. S.L. is a professor in the Department of Computer Science at the City University of Computer Science, Hong Kong. K.Z. is a professor at Wuhan National Laboratory for Optoelectronics, Huazhong University of Science and Technology, China.
