# Supplementary material for: An integrated respiratory microbial gene catalogue to better understand the microbial aetiology of Mycoplasma pneumoniae pneumonia
Source: Gigascience. 2019 Jul 31;8(8):giz093. doi: 10.1093/gigascience/giz093 (PMC6669060; doi:10.1093/gigascience/giz093)
Supplement: giz093_GIGA-D-19-00029_Revision_1 [file giz093_giga-d-19-00029_revision_1.pdf]

GigaScience

The integrated respiratory microbial gene catalogue facilitate the understanding of microbial aetiology in Mycoplasma pneumoniae pneumonia

--Manuscript Draft--

**Manuscript Number:**	GIGA-D-19-00029R1
**Full Title:**	The integrated respiratory microbial gene catalogue facilitate the understanding of microbial aetiology in Mycoplasma pneumoniae pneumonia
**Article Type:**	Research
**Funding Information:**	Key Medical Disciplines Building Project of Shenzhen (SZXJ2017005) — Mr Zhiwei Lu<br>Sanming Project of Medicine in Shenzhen (SZSM201512030) — Dr Yuejie Zheng<br>Shenzhen Science and Technology Project (JCYJ20170303155012371) — Mr Heping Wang<br>Shenzhen Science and Technology Project (JCYJ20170816170527583) — Mr Heping Wang
**Abstract:**	Background<br>An imbalanced respiratory microbiota has been observed in pneumonia which caused high morbidity and mortality in childhood. Respiratory metagenomic analysis demands a comprehensive microbial gene catalogue which will significantly advance our understanding of host-microbiota interactions.<br><br>Results<br>In this study, we collected 334 respiratory microbial samples from 171 healthy children and 76 pneumonia children. The established respiratory microbial gene catalogue (RMGC) comprised 2.25 million non-redundant microbial genes covering 90.52% prevalent genes. The core microbial species in the oropharynx (OP) of the healthy children mainly comprised Prevotella and Streptococcus. The OP microbial diversity and gene number in children with Mycoplasma pneumoniae pneumonia (MPP) decreased compared to that in healthy children, and the concurrence network of OP microbiota in patients is featured by Staphylococcus spp. and M. pneumoniae. Functional orthologues, which are associated with the metabolism of various lipids, membrane transport and signal transduction, accumulated in the OP microbiome of sick children. Several antibiotics-resistance genes (ARGs) and virulence-factor genes (VFGs) were identified in M. pneumoniae as well as other 13 microbial draft genomes, which were reconstructed via metagenomic data. Though the common macrolides/beta-lactam-resistance genes were not identified in assembled M. pneumoniae genome, a SNP mutation (A2063G) related with macrolides resistance was identified in 23S rRNA gene.<br><br>Conclusions<br>This study will facilitate exploring unknown microbial components and host-microbiota interaction in respiratory microbiome studies as well as render further insights into the microbial aetiology of MPP.
**Corresponding Author:**	Yuejie Zheng<br>Shenzhen Children's Hospital<br>Shenzhen, Guangdong CHINA
**Corresponding Author Secondary Information:**	
**Corresponding Author's Institution:**	Shenzhen Children's Hospital
**Corresponding Author's Secondary Institution:**	
**First Author:**	Wenkui Dai

Powered by Editorial Manager® and ProduXion Manager® from Aries Systems Corporation

**First Author Secondary Information:**	
**Order of Authors:**	Wenkui Dai
	Heping Wang
	Dongfang Li
	Qian Zhou
	Xin Feng
	Zhenyu Yang
	Wenjian Wang
	Chuangzhao Qiu
	Zhiwei Lu
	Ximing Xu
	Mengxuan Lyu
	Gan Xie
	Yinhu Li
	Yanmin Bao
	Yanhong Liu
	Kunling Shen
	Kaihu Yao
	Xikang Feng
	Yonghong Yang
	Shuaicheng Li
	Ke Zhou
	Yuejie Zheng
**Order of Authors Secondary Information:**	
**Response to Reviewers:**	Dear Editors and Reviewers,<br>Many thanks for your professional comments, please kindly check the responses below. As for the author list, we did adjustment based on manuscript revision, please kindly check the updated list in the manuscript.<br>Reviewer #1:<br>The authors constructed a respiratory microbial gene catalogue (RMGC) by metagenomics analysis on respiratory microbial samples from 171 healthy and 76 pneumonia children and compared the difference of gene set and microbiome between the healthy and pneumonia children. Data showed obvious difference between the two groups. I have some questions below:<br>1. Patient inclusion. This study focused on the etiology of Mycoplasma pneumoniae pneumonia (MPP), however, the enrollment criteria for the 76 patients was based on pneumonia and no specific requirement for Mpn positive test. Actually, there were only 52 patients listed in Supplemental Table 1 were Mpn positive in BALF. Then how did the comparison performed in the subsequent analysis? Were all 76 patients included or only the 52 Mpn-positive patients considered?<br>1. Response: Thanks for your questions. We aimed to construct a non-redundant integrated gene catalogue of respiratory microbes and applied the gene set to deepen the understanding of imbalanced microbial components as well as functions in Mycoplasma pneumoniae-associated pneumonia. Given different respiratory microbiota imbalance when infected by bacterial, Mycoplasma or viral pathogens, we included pneumonia children with infections of different pathogens and public respiratory microbial genomes to construct the integrated gene set. Then we focused on the imbalanced respiratory microbiome analysis for 34 patients, who were diagnosed as M. pneumoniae infection by BALF detection, compared to randomly

	selected 33 age-matched healthy children.<br><br>2. Line 146. The website is not accessible.<br>2. Response: We have updated the website of the RMGC and the URL also been changed (https://rmgc.deepomics.org), please kindly check the new URL link.<br><br>3. Line 169. Spell out "KOs" since it appeared first here in the text, not in the Methods part. Same as "CAGs" in line 193.<br>3. Response: Thanks for your kind reminding. The full names of aberrations (KOs and CAGs) were mentioned in the first appearance. KOs, KEGG Orthology; CAGs, Co-Abundance Gene Groups.<br><br>4. Line 72-73, 205-206 and 267-269. In the abstract, the authors stated "orthologues, which are associated with the metabolism of various lipids, membrane transport and signal transduction, accumulated significantly in the OP microbiome of sick children". However, in the text and Figure 5b, the enrichment is "slightly". In the discussion, the authors stated these gene functions could partly explain the reduced tight junction proteins and increased respiratory mucosa permeability after infection. Please discuss with more details and supporting references.<br>4. Response: Thanks for your careful review. We have checked the analysis results, and removed the inaccurate word “significantly” in the abstract. We also rewrote the related description in the manuscript. Please kindly check the revised manuscript.<br><br>5. Line 220-221. Please list the names of the 4 antibiotic-resistance genes.<br>5. Response: Thanks for your comment. We totally identified 4 homological antibiotic resistance genes via aligning the assembled genome to CARD (The Comprehensive Antibiotic Resistance Database), named “Mycobacterium tuberculosis gyrA conferring resistance to fluoroquinolones”, “Staphylococcus aureus rpoC conferring resistance to daptomycin”, “Staphylococcus aureus rpoB mutants conferring resistance to rifampicin” and “Escherichia coli EF-Tu mutants conferring resistance to kirromycin”. These gene names were also mentioned in Supplementary Table 4, please kindly check the revision.<br><br>6. Line 224-228. Please explain why the there are multiple copies of CARDS toxin genes (MPN372 in M129 genome) in the virulence-factor genes list. In all published Mpn genomes, CARDS toxin is a single copy gene. P1 adhesin is also a single copy gene, although it contains a repetitive element (repMp2/3) which other copies are spread across the genome. Could there be any annotation errors for the assembled Mpn genome?<br>6. Response: Thanks for your professional comments. We have reassessed the genome assembly through the published international standard developed by the Genomic Standards Consortium (GSC), and the criterion of Mpn genome is “Medium-quality draft”. Though the assembled genome size of Mycoplasma pneumonia (0.80Mb) is close to the reference genome(0.82Mb), 49 genomic fragments were not assembled. Due to the limitations of respiratory metagenomic sequencing, such as low bacterial biomass in airway samples, short paired-end reads and assembly methods, the genome sequences would be separated by homologous or repetitive sequences, and multiple gene segments could be found in different contigs. We have also rechecked the gene prediction results, it showed that multiple copies of CARDS toxin genes and P1 adhesion genes existed in different contigs. This limitation was also listed in the discussion, please kindly check the revision.<br><br>7. Line 292. "no macrolide/beta-lactam resistance genes were identified in M. pneumoniae genome". Macrolide resistance in Mpn is due to the point mutations in 23S rRNA gene. I believe 23S rRNA gene must present in the assembled Mpn genome. Please check if there are any mutations conferring macrolide resistance in 23S rRNA gene.<br>7. Response: Thanks for your professional suggestion. The RMGC was composed of coding sequences(CDS) gene rather than non-CDS gene (rRNA, tRNA). As you suggested, the sequencing data of M. pneumoniae-positive samples were further mapped against M. pneumoniae 23S rRNA gene, and we analyzed the samples with read-mapping coverage on the 23S rRNA more than 10x depths. We finally verified that 8 samples contained reliable 23S rRNA SNP mutations (A2063G), which was correlated to macrolide resistance. We have rewritten the manuscript about this SNP

description. Please kindly check the new submission.

Reviewer #2:
This is an observational metagenomic/microbiome study that examines differences in taxonomic composition and gene content between children with pneumonia and controls. It compiles a rather large sample dataset, and the authors make available a gene catalog specific to the upper respiratory tract. It seems that the respiratory microbiome was well sampled, as evidenced by the rarefaction results.
1. I think the authors need to discuss their results in light of findings from other respiratory microbiome studies (which they partially do), and more importantly, of other gene catalog efforts such as:

Qin, J., Li, R., Raes, J., Arumugam, M., Burgdorf, K.S., Manichanh, C., Nielsen, T., Pons, N., Levenez, F., Yamada, T. and Mende, D.R., 2010. A human gut microbial gene catalogue established by metagenomic sequencing. nature, 464(7285), p.59.

Li, J., Jia, H., Cai, X., Zhong, H., Feng, Q., Sunagawa, S., Arumugam, M., Kultima, J.R., Prifti, E., Nielsen, T. and Juncker, A.S., 2014. An integrated catalog of reference genes in the human gut microbiome. Nature biotechnology, 32(8), p.834.

1. Response: Thanks for your suggestion. Refer to your suggested publications, we have rewritten the discussion about the contribution and potential influence of integrated respiratory microbial gene catalogue to respiratory microbiome study. Please kindly check the revision.

General Comments
2. Is it OK to compare NP, OP, and lung microbiota from pneumonia patients with OP from controls? How do you control for site specific microbiota composition?
2. Response: Thanks for your comment. We combined the data from NP, OP and lung microbiome in both diseased and healthy children to establish the respiratory microbial gene set. Given enough data depth in OP microbiota, we applied the reference gene set to analyze microbial components, genes and functions in each OP microbial sample, and then compared the OP microbiome differences between diseased and healthy children. We did not conduct comparison between NP, OP and lung microbiome in patients with OP microbiome in healthy children. This study design has been clarified in the revision, please kindly check it.

3. Did the authors control for the differences in gender and age? Those seem to be significant as judging by the proportion of females 31% and 50% for example. Did you perform any test to check whether these differences are significant?
3. Response: Thanks for your comments. We compared the OP microbiome differences between 34 pneumonia patients (16 girls and 18 boys) and 33 age-matched random-selected healthy children (15 girls and 18 boys) with data size more than 650 Mbp. There are no significant difference of age (p-value =0.545, by t test) or gender (p-value=0.542, by Chi-square test) between two groups. We also checked other characteristics related to the microbial composition by PERMANOVA, and the “pneumonia onset” was the most significant factor (adjust p-value <0.001).

4. Assembly quality should be judge using published standards. The authors describe in detail how they obtained genomes from metagenomes. However, there are international standards regarding this, and it would be preferable that they use them so as to provide the readership with an objective point of comparison. See https://www.ncbi.nlm.nih.gov/pubmed/28787424
4. Response: Really appreciated your professional suggestions. We firstly assessed the assembly quality via 6 criteria at the “Single microbial genome assembling from OP metagenomic data” (referring to https://www.ncbi.nlm.nih.gov/pmc/articles/pmid/26425705/). As you suggested, we updated the assembly quality estimation following the published international standard developed by the Genomic Standards Consortium (GSC). The more comprehensive objective metrics of assembly quality were added in the Supplementary Table 3. Please kindly check the updated manuscript and Supplementary Table 3.

Minor Comments
5. Abstract

	Line 62 - Define RMGC<br>Line 70 - What do you mean by "simple co-occurrence network"? What's so simple and what are complex co-occurrence networks<br>Line 74 - Please do not use apostrophes<br>5. Response:<br>Line 62: RMGC is the abbreviation for respiratory microbial gene catalogue, which comprised non-redundant respiratory microbial genes.<br>Line 70: Many thanks for your professional comments and we optimized the expressions in the revision.<br>Line 74: Thanks for your reminding and we removed it as you suggested.<br><br>6. Background<br>First paragraph - Please add something related to pneumonia worldwide. The authors say it's relevant in China but I'm sure it's relevant elsewhere as well.<br>Line 87 - Is it not the etiology of MPP Mycoplasma?<br>Line 89 - Please replace "the present" with "current"<br>Line 91 - There are plenty of pipelines that do not use OTU clustering and can get taxonomic classifications down to species. For example, see https://f1000research.com/articles/5-1492/v2<br>Line 93 - The authors mention that strain level microbiome studies are needed in MPP but they do not perform any strain-level analysis<br>6. Response:<br>First paragraph: Thanks for your suggestion. The worldwide medical information of pneumonia has been added in Background paragraph, please kindly check it.<br>Line 87: Mycoplasma pneumoniae is the pathogenic agent of MPP. However, it remained to be fully understood for differed disease severity in MPP patients. We aimed to deepen the understanding of microbial contributions in MPP incidence as well as progression, as a series of reports indicated the association of respiratory microbiota with respiratory disease severity.<br>Line 89: We have replaced the "the present" with "current".<br>Line 91: Many thanks for your professional suggestions, it is really a great tool for microbial amplicon analysis to get taxonomic classifications. We will refer to these pipelines to update previous respiratory tract microbiota studies. In this study, we conducted metagenome sequencing instead of 16S rRNA analysis to build a respiratory microbial gene catalogue as well as identify microbial functions and gene elements from assembled genomes.<br>Line 93: Really appreciated for your careful and professional comment. We intended to conduct species instead of strain-level analysis, and we revised it in the revision.<br><br>7. Analyses<br>Line 136 - It is better to express this in "read numbers" instead of Gbps.<br>Line 144 - Why do authors say bacterial genes? Did you filter any non-bacterial DNA sequences/reads? If you do shotgun sequencing you will likely find fungi, viruses and others. Please explain.<br>Line 146 - The webpage https://deepomics.org/respiratory_microbial_gene_catalogue/ does not exist<br>Line 148 - Could you elaborate on why using samples with more than 650 Mb? Why not 600 or 700?<br>Line 177 - Please replace "We totally acquired" by "We acquired 67 core species in total"<br>Line 193 - Please define CAGs as on this line the acronym appears for the first time<br>Line 217 - 2.30 Mb is very low for a bacterial genome. Please elaborate on why this might be. Comment on genome completeness<br>Line 229 - What's the evidence regarding the designation of a new species? Nucleotide identity, gene content? Please discuss in the manuscript and consider some objective metric such as the Genome Taxonomy DB https://www.nature.com/articles/nbt.4229<br>Line 254 - Please revise this sentence as it is grammatically incorrect<br>7. Response:<br>Line 136: Thanks for your suggestion. We have replaced the “Gbps” by “read numbers”.<br>Line 144: We replaced this inadequate word “bacterial” by “microbial”.<br>Line 146: This website was updated recently, and moved to a new website (https://rmgc.deepomics.org). We declaimed that this website will be permanent. Please kindly check the revised manuscript.

Powered by Editorial Manager® and ProduXion Manager® from Aries Systems Corporation

	Line 148: We have drawn the curve of sequencing data size (See Supplementary Figure 3), indicating sharp decrease after 0.65 Gb data size. In Method section, each sample with ≥650 Mbp data was selected for genome assembly, and the data of other samples were mixed for genome assembly. To better assess the RMGC, we then keep the consistent criterion (more than 650 Mb in Line 148).<br>Line 177: We modified the sentence as your suggestion, thanks for your kind comment.<br>Line 193: The full words of aberrations CAGs were Co-Abundance Gene Groups, please kindly check the revised manuscript.<br>Line 217: The average bacterial genome size ranged from 1Mb to 10Mb. The completeness of assembled microbial genomes was evaluated through the international standards (https://www.ncbi.nlm.nih.gov/pubmed/28787424), and the assembled genomes were classified as "Medium-quality draft genome". Given the draft genome, several homologous or repetitive sequences would be removed in the assembly process. The detailed assessment of all 14 microbial genomes could refer to the update Table S3.<br>Line 229: Thanks for your suggestions. The species designation of 14 assembled genomes were following three evidences, including 1) concordance with taxonomic assignment of CAGs (https://www.nature.com/articles/nbt.2939); 2) aligned to the published genome sequences from IMG, NCBI and PATRIC via BLASTN (parameter: –e 0.01), with ≥95% nucleotide identity and ≥95% genome coverage; 3) assigned by CheckM from the Genome Taxonomy DB. Combined three evidences, the genome which could not be assigned to any known microbial species were defined as novel species. The detailed species designation method is added in the "Single microbial genome assembling from OP metagenomic data" paragraph of the Method section, and the updated taxonomy information could refer to Supplementary Table 3.<br>Line 254: We have revised the sentence as following: "The core microbial species of OP microbiota in healthy children will provide a reference for exploring microbial as well as host-microbe interactions in RM study."<br><br>8. Please add the BioProject instead of the SRP accession<br>8. Response: We added the BioProject ID (PRJNA413615) in the revision, please kindly check it.
**Additional Information:**	
**Question**	**Response**
Are you submitting this manuscript to a special series or article collection?	No
**Experimental design and statistics**<br><br>Full details of the experimental design and statistical methods used should be given in the Methods section, as detailed in our Minimum Standards Reporting Checklist. Information essential to interpreting the data presented should be made available in the figure legends.<br><br>Have you included all the information requested in your manuscript?	Yes
**Resources**<br><br>A description of all resources used,	Yes

including antibodies, cell lines, animals and software tools, with enough information to allow them to be uniquely identified, should be included in the Methods section. Authors are strongly encouraged to cite Research Resource Identifiers (RRIDs) for antibodies, model organisms and tools, where possible.<br><br>Have you included the information requested as detailed in our Minimum Standards Reporting Checklist?	
**Availability of data and materials**<br><br>All datasets and code on which the conclusions of the paper rely must be either included in your submission or deposited in publicly available repositories (where available and ethically appropriate), referencing such data using a unique identifier in the references and in the "Availability of Data and Materials" section of your manuscript.<br><br>Have you have met the above requirement as detailed in our Minimum Standards Reporting Checklist?	Yes

Powered by Editorial Manager® and ProduXion Manager® from Aries Systems Corporation

Manuscript-revision

Click here to access/download;Manuscript;Manuscript-revison.docx

Click here to view linked References

The integrated respiratory microbial gene catalogue facilitate the understanding of microbial aetiology in *Mycoplasma pneumoniae* pneumonia

Running Title: Airway microbial gene set and altered microbiome

~~Heping Wang*, Department of Respiratory Diseases, Shenzhen Children's Hospital, Shenzhen 518026, China; szetgmy@163.com~~

Wenkui Dai*, Department of Computer Science, City University of Hong Kong, Hong Kong 999077, China; daiwenkui84@gmail.com

Heping Wang*, Department of Respiratory Diseases, Shenzhen Children's Hospital, Shenzhen 518026, China; szetgmy@163.com

Dongfang Li*, Wuhan National Laboratory for Optoelectronics, Huazhong University of Science and Technology, No. 1037 Luoyu Road, Wuhan 430074, China; loveli_biocc@163.com

Qian Zhou~~*;~~, Department of Microbial Research, WeHealthGene Institute, Shenzhen 518000, China; zhouqian@wehealthgene.com

Xin Feng, Department of Microbial Research, WeHealthGene Institute, Shenzhen 518000, China; fengxin@wehealthgene.com

Zhenyu Yang, Department of Microbial Research, WeHealthGene Institute, Shenzhen 518000, China; yangzhy@wehealthgene.com

Wenjian Wang, Department of Respiratory Diseases, Shenzhen Children's Hospital, Shenzhen 518026, China; dhbk2005@163.com

Chuangzhao Qiu, Department of Microbial Research, WeHealthGene Institute, Shenzhen 518000, China; qiuchzh@wehealthgene.com

Zhiwei Lu, Department of Respiratory Diseases, Shenzhen Children's Hospital, Shenzhen 518026, China; luzhiwei1950@163.com

Ximing Xu, Institute of Statistics, Nankai University, No. 94 Weijin Road, Tianjin 300071, China; ximing@nankai.edu.cn

Mengxuan Lyu, Department of Computer Science, City University of Hong Kong, Hong Kong 999077, China; mengxualv2-c@my.cityu.edu.hk

Gan Xie, Department of Respiratory Diseases, Shenzhen Children's Hospital, Shenzhen 518026, China; xiegan1987@163.com

Yinhu Li, Department of Microbial Research, WeHealthGene Institute, Shenzhen 518000, China; liyh@wehealthgene.com

Yanmin Bao, Department of Respiratory Diseases, Shenzhen Children’s Hospital, Shenzhen 518026, China; baoyanming1978@163.com

Yanhong Liu, Department of Microbial Research, WeHealthGene Institute, Shenzhen 518000, China; liuyanhong@wehealthgene.com

~~Qin Yang, Department of Respiratory Diseases, Shenzhen Children’s Hospital, Shenzhen 518026, China; 6468633@qq.com~~

Kunling Shen, Department of Respiratory Diseases, Beijing Children’s Hospital, Beijing 100045, China; Department of Respiratory Diseases, Shenzhen Children’s Hospital, Shenzhen 518026, China; kunlingshen1717@163.com

Kaihu Yao, Department of Respiratory Diseases, Beijing Children’s Hospital, Beijing 100045, China; Department of Respiratory Diseases, Shenzhen Children’s Hospital, Shenzhen 518026, China; jiuhu2655@sina.com

~~Yongshun Shen~~Xikang Feng, Department of ~~Pediatrics, Shenzhen Dapeng District Maternity&Child Healthcare Hospital, No.149 Xindong Road, Shenzhen 518116~~Computer Science, City University of Hong Kong, Hong Kong 999077, China; ~~shenyongshun@sina.com~~xikangfeng2-c@my.cityu.edu.hk

Ke Zhou[#], Wuhan National Laboratory for Optoelectronics, Huazhong University of Science and Technology, No. 1037 Luoyu Road, Wuhan 430074, China; k.zhou@hust.edu.cn

Yonghong Yang[#], Department of Respiratory Diseases, Beijing Children’s Hospital, Beijing 100045, China; Department of Respiratory Diseases, Shenzhen Children’s Hospital, Shenzhen 518026, China; Department of Microbial Research, WeHealthGene Institute, Shenzhen 518000, China; yyh628628@sina.com

Shuaicheng Li[#], Department of Computer Science, City University of Hong Kong, Hong Kong 999077, China; shuaicli@cityu.edu.hk

Ke Zhou[#], Wuhan National Laboratory for Optoelectronics, Huazhong University of Science and Technology, No. 1037 Luoyu Road, Wuhan 430074, China; k.zhou@hust.edu.cn

Yuejie Zheng[#] Department of Respiratory Diseases, Shenzhen Children’s Hospital, Shenzhen 518026, China; shine1990@sina.com

*These authors contributed equally to this work.

[#]Corresponding authors

Abstract

Background: An imbalanced respiratory microbiota has been observed in pneumonia which caused high morbidity and mortality in childhood. Respiratory metagenomic analysis demands a comprehensive microbial gene catalogue which will significantly advance our understanding of host-microbiota interactions. **Results:** In this study, we collected 334 respiratory microbial samples from 171 healthy children and 76 pneumonia children. The established respiratory microbial gene catalogue (RMGC) comprised 2.25 million non-redundant microbial genes covering 90.52% prevalent genes. The core microbial species in the oropharynx (OP) of the healthy children mainly comprised *Prevotella* and *Streptococcus*. The OP microbial diversity and gene number in children with *Mycoplasma pneumoniae* pneumonia (MPP) decreased compared to that in healthy children, and the concurrence network of OP microbiota in patients is featured by simple concurrence network mediated by *Staphylococcus spp.* and *M. pneumoniae*. Functional orthologues, which are associated with the metabolism of various lipids, membrane transport and signal transduction, accumulated significantly in the OP microbiome of sick children. Among fourteen

~~reconstructed microbial genomes, *M. pneumoniae* didn’t contain macrolides/beta-lactam antibiotic~~Several antibiotics-resistance genes (ARGs) ~~which correlated with clinical medication, but these ARGs~~and virulence-factor genes (VFGs) were identified in *M. pneumoniae* as well as other 13 microbial ~~genomes.~~draft genomes, which were reconstructed via metagenomic data. Though the common macrolides/beta-lactam-resistance genes were not identified in assembled *M. pneumoniae* genome, a SNP mutation (A2063G) related with macrolides resistance was identified in 23S rRNA gene. **Conclusions:** This study will facilitate exploring unknown microbial components and host-microbiota interaction in respiratory microbiome studies as well as render further insights into the microbial aetiology of MPP.

Keywords

Pneumonia; *Mycoplasma pneumoniae*; Oropharynx; Microbiome; Respiratory microbial gene catalogue

Background

Studies have identified the indispensable respiratory microbiota[1-5] and its imbalance

Formatted

Formatted

Formatted

in pneumonia[6, 7], which is a leading cause of high morbidity and mortality in Chinese children. leading cause of high morbidity and mortality[8, 9] worldwide, especially in children under 5 years[10, 11]. In recent years, *Mycoplasma pneumoniae* pneumonia (MPP) represents increasing cases in Chinese children[8][12] and microbial aetiology remains to be explored. Our previous studies unravelled altered respiratory microbiota in children with MPP[9, 10][13, 14].

However, the presentcurrent respiratory microbiome (RM) studies have mainly focused on 16S rRNA analysis[6, 7, 11, 12]15, 16] which merely provides cues about known bacterial components at the genus level. Emerging studies that applied a 16S rRNA analysis have revealed the imbalanced microbial structure in the respiratory tracts of children with pneumonia[7, 13, 14]17, 18], but changes in the microbial functions and strainspecies-level microbial components in the RM of patients with MPP remain unexplored. In addition, current multi-omics studies are limited to explorations of known bacterial genomes in the RM[11][15]. Nevertheless, the RM includes a high proportion of unknown microbial species[1-3, 5, 6] which require further exploration.

A comprehensive catalogue of reference genes is crucial for in-depth functional

metagenomic analysis such as species/gene profiling, microbial biomarkers and functional annotation. Given that the RM varies with the environment~~[15]~~[19], age[1, 2, 4] and disease[6, 7, ~~11, 12~~][15, 16], we selected the nasopharynx (NP), oropharynx (OP) and lung samples from 76 children with pneumonia and OP samples from 171 healthy children in China to establish an integrated ~~respiratory microbial gene catalogue~~ ~~(~~RMGC~~)~~ and study the imbalanced RM in Chinese children with MPP. Using this catalogue, we assessed the microbial components and functions in the OP microbiome of healthy and MPP children as well as the characteristics of recovered microbial genomes.

Data Description

From 3 July to 27 August 2016, patients were recruited from the hospitalization zone in the Department of Respiratory Diseases of Shenzhen Children's Hospital. Inclusion criteria for patients consisted of characteristic chest radiographic abnormalities consistent with pneumonia, the exclusion of asthma, and the clearance of respiratory infections or exposure to antibiotics within one month prior to sampling (Table 1). We collected NP (25-800-A-50, Puritan, Guilford, ME, USA) and OP (155C, COPAN,

Murrieta, CA, USA) swabs from 76 hospitalized patients within 24 hours after hospitalization and before the administration of antibiotics. Bronchoalveolar lavage fluids (BALFs) were collected 2 to 15 days after hospitalization (Supplementary Table 1).

Healthy children were recruited during physical examination in summer of 2016 (from July to August) in Shenzhen. OP swabs were collected from 171 healthy children who met the following inclusion criteria: no diagnosis of asthma or a family history of allergy; no history of pneumonia; a lack of wheezing, fever, cough or other respiratory/allergic symptoms at sampling one month prior to the study and one week after sampling; no exposure to antibiotics one month prior to sampling.

All samples were collected by an experienced clinician. Samples were stored at −80°C within 20 minutes after collection and DNA was extracted within 10 days of the sampling. A TGuide S32 Magnetic Swab Genomic DNA Kit (DP603-T2, TIANGEN Biotech (Beijing) Co., Ltd., Beijing, China, http://www.tiangen.com/en/) was utilized to extract the DNA and metagenomic sequencing was performed on the Illumina Hi-Seq platform (San Diego, USA) in terms of the manufacturer's

instructions. Unused swabs and DNA extraction kits from the same batch served as negative controls to assess DNA contamination.

Analyses

Sample information and data output

Two hundred forty-seven children aged <13 years were enrolled in this study (Table 1 and Supplementary Table 1). After removing host contamination and low-quality data, metagenomic sequencing produced ~~476.62 Gbp data~~4,765,288,986 read numbers with an average of ~~1.43 Gbp~~14,267,332 read numbers per sample. DNA concentration of unused sampling swabs and DNA extraction kits was lower than 0.01 ng/µl, whereas the DNA concentration was higher than 80 ng/µl in sampling swabs and BALF. Furthermore, the DNA amplification results of extracted bacterial DNA were less than 0.01 nmol/l for the enveloped sampling or extraction materials (Supplementary Figure 1)

Construction of the RMGC

By applying metagenomics sequencing data from 247 children and three resources of respiratory related ~~bacteria~~microbial genomes/genes (Figure 1), we constructed a

comprehensive RMGC with 2,245,343 non-redundant ORFs and it was freely accessible through our website (https://rmgc.deepomics.org~~/respiratory_microbial_gene_catalogue/).~~). The total length of the ORFs in the RMGC was 1.71 Gbp and the average length was 760 nt, ranging from 102 to 32,241 nt. We selected 241 samples with ⩾650 Mb data to examine the coverage of the microbial genes in the RMGC. In accordance with the rarefaction curve, 90.52% of prevalent microbial genes were captured in the RMGC (Figure 2a and b).

Taxonomic assessment and functional annotation of the RMGC

Based on taxonomic profiling, 1,281,673 genes (57.08% of RMGC) were assigned to phyla and 1,143,382 genes (50.92% of RMGC) were assigned to genera, representing 56.58% and 51.75% of the sequencing reads respectively. A total of 617,968 genes (25.92% of RMGC) were annotated to known bacterial species, representing 33.49% of the sequencing reads. The phyla Firmicutes, Bacteroides, Proteobacteria, Actinobacteria and Fusobacteria dominated the RMGC while the prevalent microbial genera included *Staphylococcus*, *Streptococcus*, *Haemophilus*, *Corynebacterium*,

Dolosigranulum, *Prevotella*, *Blautia*, *Rothia*, *Porphyromonas*, *Lactobacillus*, *Veillonella*, *Fusobacterium* and *Leptotrichia*. Unknown microbial species accounted for 9.62% to 55.50% of the RMGC and the detailed taxonomic information of RMGC was deposited on our website.

The genus-level microbial structure revealed by metagenomic analysis resembled the results of the 16S rRNA analysis (Supplementary Figure 2). Notably, a greater proportion of microbial genera remained unclassified in the metagenomic analysis than in the 16S rRNA analysis, which might be attributed to the wide detection by metagenomics sequencing and limited reference microbial genomes.

By aligning RMGC to KEGG database, a total number of 6,408 ~~KOs~~KEGG Orthology (KO) were identified, including 853,446 genes representing 37.85% of the total sequencing data. Known microbial functions (annotated by KEGG) saturated quickly to 6,346 groups as more samples were included (Figure 2c). Combined novel gene families, the rarefaction curve plateaued when 12,924 groups were detected (Figure 2c). Upon alignment to the eggNOG database, 53.95% of the genes in the RMGC were assigned to known functional categories.

Core microbial species in OP microbiome of healthy children

We ~~totally~~ acquired 67 core species in total in 5 dominant phyla Bacteroidetes, Firmicutes, Proteobacteria, Actinobacteria and Fusobacteria (Figure 3). *Prevotella melaninogenica* (4.38±2.91%, mean±sd), *Prevotella sp.* (3.06±1.92%), *Prevotella histicola* (3.23±3.58%), *Prevotella pallens* (2.31±1.88%) and *Veillonella atypical* (1.60±1.44%) were the top 5 microbial species. In addition, *Streptococcus pseudopneumoniae* (1.26±0.96%), ~~*H.*~~ *Haemophilus influenzae* (0.60±0.68%), ~~*S.*~~*Streptococcus pneumoniae* (0.60±0.50%), *Haemophilus parainfluenzae* (0.42±0.49%) and ~~*S.*~~*Staphylococcus aureus* (0.27±1.52%), which were generally defined as opportunistic pathogens, were also prevalent in OP microbiome of healthy children (Figure 3).

Microbial structure and functions in OP microbiome of MPP patients differed from that in healthy children

Based on the PERMANOVA, pneumonia onset is the most significant factor (adjust *p*-value <0.001) explaining the variations in OP microbial samples, followed by feed pattern (adjust *p*-value = 0.037) and age (adjust *p*-value = 0.048). Compared with

healthy children, MPP patients exhibited significantly decreased microbial gene number and alpha diversity of the OP microbiome (Figure 4a and b). Moreover, thirty Co-Abundance gene Groups (CAGs) accumulated significantly in the OP microbiome of MPP patients, comprising 6 unknown and 24 known microbial species which were primary respiratory pathogens such as *M. pneumoniae*, *Staphylococcus epidermidis* and *S. aureus* (Figure 5a). Ninety-five CAGs were enriched in the OP microbiome of healthy children including prevalent colonizers such as *Prevotella* species (Figure 5a). The microbial co-occurrence networks in MPP patients were simpler than that in healthy children and negative correlations were only identified between health-enriched and MPP-enriched CAGs (Figure 5a). For example, health-enriched *Prevotella spp.* were negatively correlated with MPP-enriched *S. epidermidis* ($r<-0.60$, adjust *p*-value $\leqslant 0.05$, Figure 5a).

By comparing functional annotations via KEGG annotation (Supplementary Table 2), we assessed the functional alterations of the OP microbiome in patients with MPP. Microbial functions which related to lipid metabolism, membrane transport and signal transduction were slightly enriched in MPP patients (Figure 5b). In contrast, the

OP microbiome of healthy children was significantly enriched in ~~orthologues~~orthologous involved in glycan biosynthesis and metabolism, biosynthesis of secondary metabolites, and cell growth and death (Figure 5b and Supplementary Table 2). Host homeostatic associated functions, such as immune system, digestive system, circulatory system and environmental adaptation were also significantly abundant in the OP microbiome of healthy children (Figure 5b and Supplementary Table 2).

Characterization of the *M. pneumoniae* genome and other 13 re-constructed microbial genomes

We re-assembled 14 qualified microbial CAGs (Supplementary Table 3) which represented *M. pneumoniae* genome (0.80 Mbp) and 13 other microbial genomes (genome sizes averaged 2.30 Mbp). The *M. pneumoniae* genome accumulated significantly in OP microbiome of MPP patients and exhibited high similarity with reference genome (97.79% of genome coverage) (Supplementary Table 3). *M. pneumoniae* genome consisted of 4 antibiotic-resistance genes (ARGs) with common antibiotics, including peptide, rifamycin and fluoroquinolone antibiotics (Figure 6,

Supplementary Table 4~~) while~~). On the other hand, SNP mutation A2063G related to macrolides-resistance was identified in 23S rRNA gene in 8 MPP ~~children~~patients, who were given experimental macrolides or beta-lactams such as azithromycin, erythromycin or sulbactam (Supplementary Table 1). In addition, there were 136 virulence-factor genes (VFGs) along its reassembled genome sequence (Supplementary Table 5) and the redundant VFGs of *M. pneumoniae* enriched in the secretion of adhesin P1, cytadherence protein and community-acquired respiratory distress syndrome (CARDS) toxin (Figure 6 and Supplementary Table 5).

Among other 13 microbial genomes, 5 of them can be designated specific species, one just be annotated at genus level (*Ralstonia*) and the rest 7 were novel microbial genomes (averaged 1.74 Mbp) (Supplementary Table 3). For the 5 annotated microbial species, *S. aureus* and *S. epidermidis* increased significantly in MPP patients while the other 3 *Prevotella spp.* mainly accumulated in healthy children (Figure 7, Supplementary Table 3). The largest reassembled *Ralstonia* genome (5.89Mbp) carried numerous ARGs, including 13 beta-lactam antibiotic genes, 21 tetracycline antibiotic genes, and 11 macrolide antibiotic genes. *P. histicola*, *P. shahii*

and CAG00068 all had one copy of macrolide antibiotic resistance and beta-lactam antibiotic resistance gene. These genomes also harboured abundant resources of VFGs which ranged from 105 to 808 copies of relative genes. According to the correlation analysis, we didn't identify the significant correlation between 14 reassembled microbial genomes and 5 clinical indexes (Supplementary Table 6).

Discussion

MPP has been causing the increasing morbidity and mortality in Chinese children. The development of RM studies has improved our understanding of the microbial aetiology of MPP by revealing infection-associated RM imbalances~~[9, 10]~~[13, 14]. However, microbial functions and host-microbiota interactions in the RM of patients with MPP remain to be explored, particularly those from novel microbial ~~strains.~~ species.

In recent years, several reference gut microbial catalogues were constructed to promote understanding of the host-microbe interaction. Qin *et al.* built a global view of the human gut microbiome (GM) and revealed a comprehensive functional potential of the prevalent gut microbial genes[20]. Li *et al.* upgraded the gut gene

catalogue in 2014[21], enabling the studies of association of the microbial genes with human health. Based on these frameworks, researchers could deepen the understanding of the correlation between GM and various diseases, such as gastrointestinal and cardiovascular diseases[22, 23].

Similar to reference gene catalogues of the gut microbiome (GM)[16-18] theGM, RMGC will further understanding of microbial aetiology in respiratory diseases. The development of a well-established RMGC in this study is crucial for the functional metagenomics analysis to improve our understanding ofknowledge about host-microbiota interactions in MPP. By aligning metagenomics data directly with the established RMGC, we profiled researchers could profile all microbial species as well as explore microbial functions in both known and unknown microbial species. The similar microbial species to that identified in theassignment between RMGC-based and 16S rRNA analysis, suggesting also suggested promising taxonomic assignments based on thesevia our constructed gene sets. The core microbial species of OP microbiota in healthy children founded a reliable reference towill provide a standard control database reference for exploring microbial as well as host-microbe

interactions in RM study[19][24] and mine the potential beneficial bacteria[20].. In general, RMGC furnishes a comprehensive respiratory associated microbial profile to forward the microbiome analysis at species/strain level and the functional profiling will facilitate in-depth multi-omics analyses[21, 22][25, 26], such as associations of produced proteins or metabolites with known and novel microbial genomes. This capability would clarify the interactions between the host and the RM alteration during MPP progression.

——The OP microbiome of MPP children changed to be simpler structure compared to that of healthy children. Previous studies revealed that bacteria-like *M. pneumoniae* could deplete bacteria through direct competition and activate the bacterial clearance factors in host responses[23, 24][27, 28], which led to decreased colonizer *Prevotella spp.*[25][29] and pathogens proliferation such as *S. aureus* and *S. epidermidis*. The MPP patient-enriched gene functions involved in membrane transport and various nutrients metabolism which could partly explain reduced tight junction proteins and increased respiratory mucosa permeability after infection[26][30]. In addition, a number of studies have identified an increased glucose concentration in

Formatted: Indent: First line: 0.29"

airway surface liquids~~[27-29]~~[31-33] and associated pathogen proliferation~~[30]~~[34], which also corroborate the enriched nutrients uptake pathways in OP microbiome of MPP patients. Though the mechanism of *M. pneumoniae* clearance in respiratory system remains unclear, these findings would render a new insight into host-microbiota interactions in MPP infection.

~~Except for well-known microbes, respiratory tracts also harboured a variety of undiscovered microbial species[31]. Moreover, recent reports had proved that single bacterial genome could be well recovered via reference gene sets and metagenomics data~~Except for well-known microbes, respiratory tracts also harboured a variety of undiscovered microbial species[35]. Moreover, recent reports had proved that single bacterial genome could be well recovered via reference gene sets and metagenomics data~~[32, 33]~~[36, 37]. Culturing of *M. pneumoniae* is rarely and difficultly used in clinical diagnosis, limiting the understanding of antibiotics resistance and virulence~~[34]~~[38] in *M. pneumoniae*. Re-construction of~~ a high quality *M. pneumoniae* genome by employing RMGC and metagenomic data indicated various ARGs which were related to RNA transcription[35], DNA replication[36] and protein synthesis[37].~~ the *M. pneumoniae*

genome by employing RMGC and metagenomic data indicated various ARGs which were related to RNA transcription[39], DNA replication[40] and protein synthesis[41]. According to clinical practice guidelines[38-40][42-44] and ARGs existence, most of MPP children were treated with azithromycin, erythromycin or sulbactam which were not associated with identified ARGs in *M. pneumoniae* genome. Increasing reports demonstrated that the specific dominated bacteria associated with severe acute respiratory infections (ARIs)[6, 41, 42]45, 46], but no meaningful correlations were identified between disease severity and *M. pneumoniae*, as well as other reassembled bacteria in OP microbiome of MPP patients. This was also identified by our previous studies which confirmed the succession of *M. pneumoniae* infection in NP to OP and lung as well as the association of *M. pneumoniae* load in the lung microbiota with disease severity[10][14].

Though no macrolide/beta-lactam resistance genes were identifieddiscovered in *M. pneumoniae* genome, one SNP mutation (23S RNA, 2063A->G) correlated to macrolide resistance were identified in MPP patients. Meanwhile, the patient-enriched microbial genomes such as *Ralstonia*, consisted plenty of ARGs related to the

resistance to macrolide, beta-lactam and tetracycline. Given rigorous antibiotic selective pressure and complex microbial interaction, the environmental redundant genetic components would rapidly transferred into the pathogen genome by horizontal gene transfer~~[43, 44]~~[47, 48] and caused several emergence diseases, such as European enterohemorrhagic *Escherichia coli* breakout~~[45]~~[49] and emergence of scarlet fever *Streptococcus pyogenes* in Hong Kong~~[46]~~[50]. Considering above-mentioned researches, we should recognize that current medications for the *M. pneumoniae* treatment hold the potential to trigger emerging drug-resistance microbial ~~strains~~species in *M. pneumoniae* or other novel microbial ~~strains~~species, such as reported macrolide~~-~~ resistance~~-~~ in *M. pneumoniae*-PCR-positive children~~[47-49]~~[51-53]. The OP microbiome also recovered several healthy enriched bacterial genomes, among which *Prevotella spp.* played as key players in OP microbiome of healthy children~~[50, 51]~~[54, 55] and other novel microbes might function as pathogen competitors such as *Vampirovibrio*~~[52]~~[56]. In general, recovered microbial genomes in respiratory tracts hold the potential to improve the understanding of microbial aetiology in MPP pneumonia.

There are several limitations to be clarified in this study. Given no efficient medicines for MPP, the inpatients accepted empirical treatments and might shift the airway ecology slightly[53][57]. Despite the promising application of the RMGC, unclassified CAGs and novel gene families in RMGC must be annotated and further explored. The copy numbers of several genes need further assessment due to potential inaccuracy caused by the low respiratory bacterial biomass, NGS sequencing and assembly methods. The respiratory microbial samples were obtained from Chinese children in this study, and more metagenomics data will be incorporated into the RMGC in the future to construct a broader characterization of microbial components and functions, as the continual updates of the GM reference genes[16-18][21, 58, 59]. This procedure will incrementally improve studies of the imbalanced RM in patients with respiratory diseases. Alterations in the OP microbiome in Chinese patients with MPP will also provide extensive insights into the microbial aetiology of acute respiratory infection.

Potential implications

Established respiratory microbial gene catalogue will ensure deepen respiratory micro-ecology research, which holds the promise to elucidate respiratory microbial

community at microbial species ~~or even strain~~ level. In addition, genomes of novel microbial genera or species can be assembled through aligning metagenomics data with the reference catalogue. Exploring microbial functions and associated microbial components can construct the microbial network in respiratory microbial community. Established reference gene sets can be employed to deepen multi-omics analysis, which will further the understanding of host-microbiota interactions in acute respiratory infection. Comparing oropharynx microbiome between healthy and diseased children also provides an example for the utilization of the gene sets.

Methods

Ethics statement

We obtained approval for this study from the Ethical Committee of Shenzhen Children's Hospital (Shenzhen, Guangdong Province, China) under registration number 2016013 and performed experiments under the relevant guidelines and regulations. All guardians of selected children provided the informed consents.

Clinical detection of infectious pathogens

BALF was employed to establish the common clinical microbial diagnosis. Culturing was conducted to detect ~~*Streptococcus*~~*S. pneumoniae*, ~~*Haemophilus influenzae*~~*H. influenzae*, *Moraxella catarrhalis*, ~~*Staphylococcus*~~*S. aureus* and *Staphylococcus*

haemolyticus. The D3 Ultra DFA Respiratory Virus Screening & ID Kit (Diagnostic Hybrids, Inc., Athens, OH, USA) was employed to detect common viruses, including adenovirus (AdV), respiratory syncytial virus (RSV), influenza virus and parainfluenza virus. Cytomegalovirus (CMV) and Epstein-Barr virus (EBV) were detected via the Diagnostic Kit for Quantification of Human CMV DNA and EBV Polymerase Chain Reaction (PCR) Fluorescence Quantitative Diagnostic Kit, respectively (DaAnGene, Guangzhou, China, http://daan.joomcn.com/). *M. pneumoniae* and *Chlamydia* ~~*pneumoniae*~~ *pneumonia* were diagnosed via the diagnostic kit for *M. pneumoniae* DNA (PCR Fluorescence Probing) (DaAnGene) and Anti *C. pneumoniae* ELISA (IgM) (EUROIMMUN AG, Lübeck, Germany) respectively.

Construction and annotation of the RMGC

Sequencing data were filtered using a previously reported method~~[54]~~[60] and each sample with ≥650 Mbp data (Figure 1, Supplementary Figure 3) was selected for genome assembly by SOAPdenovo ~~[55]~~[61] (v2.07, –F –K 39 –M 3 –d 1). For samples with <650 Mbp data, the data from the same respiratory site were mixed and

assembled (Figure 1). Assembled contigs with ⩾500 bps were selected for gene prediction with MetaGeneMark[62] (v3.26, default parameters). We applied Glimmer3.02[63] (default parameters) to predict genes from the 1,384 respiratory bacterial genomes in the IMG database (2016-12-21, https://img.jgi.doe.gov/). Gene sequences were also retrieved from the genomes of 73 respiratory bacteria in PATRIC database (2017-3-25, https://www.patricbrc.org/) and 450,204 open reading frames (ORFs) of respiratory bacteria in Human Microbiome Project (HMP). Genes with ⩾100 bp length and without Ns were selected to construct non-redundant gene sets using CD-HIT[64] (v4.66, -c 0.95 –aS 0.9). Genes with ⩾2 mapped reads were retained in the established RMGC.

The taxonomic annotation of genes was conducted in the light of the following steps: i) we retrieved bacterial and viral genome sequences from IMG (2016-12-21), NCBI (2016-08-09) and PATRIC (2017-03-25) databases. We selected the genome sequence with the longest N50 as the representative genome for each bacterial species. Non-redundant viral genomes were produced by CD-HIT (v4.66, -aS 0.95 -AL 0.9 -aL 0.9 -AS 0.95 M 0). We aligned the gene sets in the RMGC to 6,869

representative bacterial genomes and 18,916 non-redundant viral DNA genomes using BLASTN (v2.5.0, default parameters except –e 0.01); ii) we retained the top 10% highest-scoring alignments of each gene, with ⩾65% identity and ⩾80% coverage of gene length; and iii). The assignment of each gene was determined based on ⩾50% consensus above the similarity threshold for a specific rank: ⩾65% for phylum, ⩾ 85% for genus and ⩾95% for species.

The functional annotation of each gene was determined by searching protein sequences in Kyoto Encyclopedia of Genes and Genomes (KEGG) (v78.1) and eggNOG (version 4.0) with BLASTP (v2.5.0, default parameters, except ~~for evalue 1e-5). The best-hit alignment (identity ⩾30% and coverage ⩾70%) was selected as the functional annotation for the gene. Genes without annotations in KEGG were identified as novel gene families by the Markov Cluster Algorithm (MCL)[59]~~ -e 1e-5). The best-hit alignment (identity ⩾30% and coverage ⩾70%) was selected as the functional annotation for the gene. Genes without annotations in KEGG were identified as novel gene families by the Markov Cluster Algorithm (MCL)[65] (inflation factor=1.1, bit-score cut-off=60).

Comparing the taxonomic assessment between 16S rRNA gene analysis and metagenomic analysis

We selected 72 OP microbial samples with ≥650 Mb metagenomic sequencing data and aligned the sequencing data to establish RMGC to determine taxonomic assignments. The same samples were also sequenced via V3-V4 region of the 16S rRNA gene[9][13]. Microbial compositions were compared between two methods to assess the accuracy of taxonomic assignments via metagenomic analysis.

Rarefaction analysis

We downsized the number of mapped reads to 3 million for each sample to eliminate the variable influence caused by the amount of sequencing data. Estimation of total gene richness was done by randomly sampling five individuals 1,000 times with gene counting and Chao2 richness estimator[60].[66].

For the rarefaction curve of KEGG orthologous groups (KOs) and novel gene families, random sampling of five individuals for 1,000 times was used to evaluate saturation. Relative rarefaction curves were visualized using R software (v3.3.2).

Calculation of gene relative abundance in RMGC

All filtered reads of metagenomics data from each sample were aligned to the established RMGC using BWA (v0.7.13, default parameters, except for the mem and identity ⩾95%). Alignments that met the following two criteria were accepted: i) paired-end reads were mapped onto a same gene with the correct insert size; and ii) one end of a paired-reads was mapped onto the end of a gene, while the other was located outside of the gene.

If the number of genes in a given sample was n, the relative abundance was calculated using the following steps:

Step 1. The copy number of the gene *i* ($c(i)$) was calculated as:

$$c(i) = \frac{t(i)}{l(i)}$$

$t(i)$: The total number of mapped reads of gene *i* in a given sample.

$l(i)$: The length of the gene *i*.

Step 2. The relative abundance of gene *i* ($Ab\_g(i)$) was defined as:

$$Ab\_g(i) = \frac{c(i)}{\sum_{1}^{n} c(i)}$$

Step 3. If m genes can be assigned to the phylogenetic assignment s, the abundance of this phylogenetic assignment ($Ab\_p(s)$) was calculated using the

following equation:

$$Ab\_p(s) = \sum_{1}^{m} Ab\_g(j)$$

Phylogenetic and functional profile of the OP microbiome

All filtered reads of the OP microbiome were aligned to the established RMGC using BWA with same parameter as above. The relative abundance of each phylogenetic assignment was calculated as showed above while the abundance of KOs in the functional profiling table was determined as described in a previous report[16][58].

Identification of OP core microbial species in healthy children

The microbial species was selected as core species if it existed in over 50% of healthy children and represented more than 1% relative abundance in one OP microbial sample. The distributions of core microbial species in OP of healthy children were described using ggplot2 in R.

Comparison of the OP microbiome between healthy children and MPP patients

According to the age distributions of 34 MPP patients (data size ≥650 Mbp), 33 randomized healthy children with similar age were chosen. Genes in the OP microbiome of selected microbial samples were clustered into co-abundance gene

groups (CAGs) via Capony-based algorithms[61][67] (default parameters). The selected CAGs which contained more than 700 genes were regarded as deriving from the same bacterial genome and selected to construct a correlation network using Spearman's rank coefficient (⩽−0.6 or ⩾0.6). The co-occurrence network was visualized using Cytoscape (v3.4.0)[62][68]. If ⩾50% of the included genes had consensus phylogenetic annotations, corresponding CAG was assigned to a related microbial taxonomic assignments.

The relative abundance of each CAG in microbial samples was calculated as previously reported[18][59]. Inter-group comparisons of CAGs and KEGG functions were performed using the two-tailed Wilcoxon rank-sum test and corrected via the Benjamini-Hochberg method (adjusted *p*-value ⩽ 0.05). Confounding factors including pneumonia, sex, age, delivery mode and feed pattern were also assessed using PERMANOVA by vegan package (v2.3-4) in R software.

Single microbial genome assembling from OP metagenomic data

OP metagenomic data were aligned to the filtered CAGs (containing ⩾700 genes) by BWA (v0.7.13, identity ⩾95%). The mapped reads of each CAG were extracted for

microbial genomes assembling with Velvet~~[63]~~[69] (kmer: from 45 to 75, cov_cutoff: auto, exp_cov: auto). The assembled sequences with the longest contig N50 were selected as representative draft genomes. Assembly quality was assessed following six criteria~~[64]~~[23]: (i) 90% of the genome assembly should be included in contigs >500 bp; (ii) 90% of the assembled bases are at >5× read coverage; (iii) contig N50 >5 kb; (iv) scaffold N50 >20 kb; (v) average contig length is >5 kb; (vi) >90% of core genes are present in the assembly. A total of 14 draft microbial genomes passed five or six criteria finally~~. And~~ (Supplementary Table 3). We then applied the ~~selected~~assembly quality estimation standard published by the Genomic Standards Consortium (GSC) (Supplementary Table 3)[70]. The microbial species designation of 14 assembed genome sequences ~~were aligned to NCBI database to obtain their taxonomic information via MUMmer (v3.0)[64, 65]. Furthermore, gene prediction was executed for the assembled genomes~~followed these standards: 1) concordance with ~~Glimmer3.02 while related annotations of antibiotic resistance and virulence were acquired through CARD~~taxonomical assignment of CAGs ~~[66]~~[67] ~~and VFDB[67].~~; 2) aligned to the published genome sequences from IMG, NCBI and PATRIC via BLASTN (v2.5.0,

Formatted

Formatted

default parameters except –e 0.01), with ≥95% nucleotide identity and ≥95% genome coverage; 3) assigned by the CheckM(v1.0.12, default parameters) from the Genome Taxonomy DB[71]. Furthermore, gene prediction was executed with Glimmer3.02, while related annotations of antibiotic resistance and virulence genes were acquired through CARD[72] and VFDB[73]. The SNP mutation associated with macrolide resistance of *M. pneumoniae* was identified by mapping sequencing reads against 23S rRNA genes[74] using BWA.

Correlations between reassembled microbial genomes and disease severity in MPP patients

The correlation between reconstructed microbial genomes with the hospitalization duration and fever peak was assessed. In addition, serum CRP, PCT and eosinophil in 24 hours after hospitalization were also selected to assess the correlation with reassembled microbial genomes via R software. The distributions of relative abundance of 14 reassembled genomes in MPP and healthy children were showed via scatter plot.

Availability of supporting data and materials

The BioProject ID is PRJNA413615. The sequencing data supporting the results of this article are available in the GenBank repository under accession number: SRP119571. The RMGC data set is available- in the GigaScience.

Declaration

List of abbreviations

AdV: adenovirus; ARI: acute respiratory infection; BALF: broncho-alveolar lavage fluid; CAGs: co-abundance gene groups; CMV: Cytomegalovirus; EBV: Epstein-Barr virus; GM: gut microbiome; KEGG: Kyoto Encyclopedia of Genes and Genomes; KOs: KEGG orthologous groups; MCL: Markov Cluster Algorithm; NP: nasopharynx; OP: oropharynx; ORFs: open reading frames; PCA: principal component analysis; PCR: Polymerase Chain Reaction; PERMANOVA: Permutational multivariate analysis of variance analysis; PP: pediatric pneumonia; RM: respiratory microbiome; RMGC: respiratory microbial gene catalogue; RSV: respiratory syncytial virus;

Consent for publication

All the guardians of participates consent to publish

Competing Interests

The authors declare no competing financial interests.

Funding

This study was supported by ~~Guangdong~~Key Medical ~~Research Fund (A2016501~~Disciplines Building Project of Shenzhen (SZXJ2017005), Sanming Project of Medicine in Shenzhen (SZSM201512030), and Shenzhen Science and Technology Project (JCYJ20170303155012371 and JCYJ20170816170527583~~) and Key Medical Disciplines Building Project of Shenzhen (SZXJ2017005). ~~).

Authors' contributions

Y.Z., Y.Y. and K.Z. managed the project. Z.L., G.X., Y.B. and Y.S. performed the sampling and information collection. W.W. and Q.Y. prepared the DNA extraction. D.L., Q.Z., X.F. and Z.Y. performed the bioinformatics analysis in this work. C.Q., Y.L. and Y.L. optimized the graphs. X.X. and M.L. optimized the data curation. S.L. and Y.Y. guided data interpretation. H.W. and W.D. dealt the data mining and wrote the paper. K.S. and K.Y. polished the article. All authors reviewed this manuscript.

Acknowledge

We thank suggestions from members in Collaborating Group of Pediatric Respiratory

Microbiome, Chinese Pediatric Society and Chinese Medical Association. We also thank Mr. Xiaofeng Lin from EasyPub for polishing language when preparing this submission.

Authors' information

Y.Y. is a Russian academician on pediatric and vaccine research. Y.Z is the director of respiratory disease department in Shenzhen Children’s Hospital. S.L is a professor of department of computer science in the City University of Computer Science. K. Z is a professor of Wuhan National Laboratory for Optoelectronics, Huazhong University of Science and Technology.

References

Formatted: Font: Not Bold

1. Stearns JC, Davidson CJ, McKeon S, Whelan FJ, Fontes ME, Schryvers AB, *et al.* Culture and molecular-based profiles show shifts in bacterial communities of the upper respiratory tract that occur with age. ISME J. 2015; 9**:** 1268.

2. Biesbroek G, Tsivtsivadze E, Sanders EA, Montijn R, Veenhoven RH, Keijser BJ, *et al.* Early respiratory microbiota composition determines bacterial

succession patterns and respiratory health in children. Am J Respir Crit Care Med. 2014; 190**:** 1283-92.

3. Biesbroek G, Bosch AA, Wang X, Keijser BJ, Veenhoven RH, Sanders EA*, et al.* The impact of breastfeeding on nasopharyngeal microbial communities in infants. Am J Respir Crit Care Med. 2014; 190**:** 298-308.

4. Bosch AA, de Steenhuijsen Piters WA, van Houten MA, Chu M, Biesbroek G, Kool J*, et al.* Maturation of the infant respiratory microbiota, environmental drivers and health consequences: a prospective cohort study. Am J Respir Crit Care Med. 2017; 196**:** 1582-90.

5. Charlson ES, Bittinger K, Haas AR, Fitzgerald AS, Frank I, Yadav A*, et al.* Topographical continuity of bacterial populations in the healthy human respiratory tract. Am J Respir Crit Care Med. 2011; 184**:** 957-63.

6. de Steenhuijsen Piters WA, Huijskens EG, Wyllie AL, Biesbroek G, van den Bergh MR, Veenhoven RH*, et al.* Dysbiosis of upper respiratory tract microbiota in elderly pneumonia patients. ISME J. 2016; 10**:** 97-108.

7. Sakwinska O, Bastic Schmid V, Berger B, Bruttin A, Keitel K, Lepage M*, et al.*

Nasopharyngeal microbiota in healthy children and pneumonia patients. J Clin Microbiol. 2014; 52**:** 1590-4.

88. Prina E, Ranzani OT, Torres A. Community-acquired pneumonia. Lancet. 2015; 386**:** 1097-108.

9. Musher DM, Thorner AR. Community-acquired pneumonia. N Engl J Med. 2014; 371**:** 1619-28.

10. Liu L, Oza S, Hogan D, Perin J, Rudan I, Lawn JE, *et al.* Global, regional, and national causes of child mortality in 2000-13, with projections to inform post-2015 priorities: an updated systematic analysis. Lancet. 2015; 385**:** 430-40.

11. Dagan R, Bhutta ZA, de Quadros CA, Garau J, Klugman KP, Khuri-Bulos N, *et al.* The remaining challenge of pneumonia: the leading killer of children. Pediatr Infect Dis J. 2011; 30**:** 1-2.

12. Qin Q, Baoping Xu, Liu X, Shen K. Status of Mycoplasma pneumoniae pneumonia in chinese children: a systematic review. Advances in Microbiology. 2014; 4**:** 704-11.

Formatted: Font: Not Italic

~~9~~13. Lu Z, Dai W, Liu Y, Zhou Q, Wang H, Li D, *et al.* The alteration of nasopharyngeal and oropharyngeal microbiota in children with MPP and non-MPP. Genes (Basel). 2017; 8.

~~10~~14. Dai W, Wang H, Zhou Q, Feng X, Lu Z, Li D, *et al.* The concordance between upper and lower respiratory microbiota in children with Mycoplasma pneumoniae pneumonia. Emerg Microbes Infect. 2018; 7**:** 92.

~~11~~15. Hasegawa K, Mansbach JM, Ajami NJ, Espinola JA, Henke DM, Petrosino JF, *et al.* Association of nasopharyngeal microbiota profiles with bronchiolitis severity in infants hospitalised for bronchiolitis. Eur Respir J. 2016; 48**:** 1329-39.

~~12~~16. de Steenhuijsen Piters WA, Heinonen S, Hasrat R, Bunsow E, Smith B, Suarez-Arrabal MC, *et al.* Nasopharyngeal microbiota, host transcriptome, and disease severity in children with respiratory syncytial virus infection. Am J Respir Crit Care Med. 2016; 194**:** 1104-15.

~~13~~17. Pettigrew MM, Gent JF, Kong Y, Wade M, Gansebom S, Bramley AM, *et al.* Association of sputum microbiota profiles with severity of

Formatted: Font: Not Italic

community-acquired pneumonia in children. BMC Infect Dis. 2016; 16**:** 317.

~~14~~18. Vissing NH, Chawes BL, Bisgaard H. Increased risk of pneumonia and bronchiolitis after bacterial colonization of the airways as neonates. Am J Respir Crit Care Med. 2013; 188**:** 1246-52.

~~15~~19. Mika M, Mack I, Korten I, Qi W, Aebi S, Frey U, *et al.* Dynamics of the nasal microbiota in infancy: a prospective cohort study. J Allergy Clin Immunol. 2015; 135**:** 905-12.e11.

~~16~~20. Zhang R, Wang H, Deng J. A 4-Year-Old Girl With Progressive Cough and Abnormal Blood Smear. Clinical Infectious Diseases. 2017; 64**:** 1630–31.

21~~. Qin J, Li R, Raes J, Arumugam M, Burgdorf KS, Manichanh C, *et al.* A human gut microbial gene catalogue established by metagenomic sequencing. Nature. 2010; 464**:** 59-65.~~

~~17~~. Li J, Jia H, Cai X, Zhong H, Feng Q, Sunagawa S, *et al.* An integrated catalog of reference genes in the human gut microbiome. Nat Biotechnol. 2014; 32**:** 834-41.

~~18~~22. Qin J, Li Y, Cai Z, Li S, Zhu J, Zhang F, *et al.* A metagenome-wide association

study of gut microbiota in type 2 diabetes. Nature. 2012; 490**:** 55-60.

23. Zhang C, Yin A, Li H, Wang R, Wu G, Shen J, *et al.* Dietary modulation of gut microbiota contributes to alleviation of both genetic and simple obesity in children. EBioMedicine. 2015; 2**:** 968-84.

24. ~~Lloyd-Price J, Mahurkar A, Rahnavard G, Crabtree J, Orvis J, Hall AB, *et al.* Strains, functions and dynamics in the expanded Human Microbiome Project. Nature. 2017; 550**:** 61-66.~~

~~19~~. Rosas-Salazar C, Shilts MH, Tovchigrechko A, Schobel S, Chappell JD, Larkin EK, *et al.* Differences in the nasopharyngeal microbiome during acute respiratory tract infection with human rhinovirus and respiratory syncytial virus in infancy. J Infect Dis. 2016; 214**:** 1924-28.

~~20. Olson CA, Vuong HE, Yano JM, Liang QY, Nusbaum DJ, Hsiao EY. The gut microbiota mediates the anti-seizure effects of the ketogenic diet. Cell. 2018; 173: 1728-41.~~

~~21~~25. Stewart CJ, Mansbach JM, Wong MC, Ajami NJ, Petrosino JF, Camargo CAJ, *et al.* Associations of nasopharyngeal metabolome and microbiome with

severity among infants with bronchiolitis: a multi-omic analysis. Am J Respir Crit Care Med. 2017; 196**:** 882-91.

~~22~~26. Quinn RA. Integrating microbiome and metabolome data to understand infectious airway disease. Am J Respir Crit Care Med. 2017; 196**:** 806-07.

~~23~~27. Yang J, Hooper WC, Phillips DJ, Talkington DF. Cytokines in Mycoplasma pneumoniae infections. Cytokine Growth Factor Rev. 2004; 15**:** 157-68.

~~24~~28. Peteranderl C, Sznajder JI, Herold S, Lecuona E. Inflammatory responses regulating alveolar ion transport during pulmonary infections. Front Immunol. 2017; 8**:** 446.

~~25~~29. Miller SI, Ernst RK, Bader MW. LPS, TLR4 and infectious disease diversity. Nat Rev Microbiol. 2005; 3**:** 36-46.

~~26~~30. Patkee WR, Carr G, Baker EH, Baines DL, Garnett JP. Metformin prevents the effects of Pseudomonas aeruginosa on airway epithelial tight junctions and restricts hyperglycaemia-induced bacterial growth. J Cell Mol Med. 2016; 20**:** 758-64.

~~27~~31. Hewitt R, Webber J, Farne H, Trujillo-Torralbo M-B, Footitt J, Molyneaux PL,

Formatted: Font: Not Italic

Formatted: Font: Not Italic

et al. Airway glucose in virus-induced COPD exacerbations. Am J Respir Crit Care Med. 2016; 192**:** A6323.

~~28~~32. Garnett JP, Nguyen TT, Moffatt JD, Pelham ER, Kalsi KK, Baker EH, *et al.* Proinflammatory mediators disrupt glucose homeostasis in airway surface liquid. J Immunol. 2012; 189**:** 373-80.

~~29~~33. Kalsi KK, Baker EH, Fraser O, Chung YL, Mace OJ, Tarelli E, *et al.* Glucose homeostasis across human airway epithelial cell monolayers: role of diffusion, transport and metabolism. Pflugers Arch. 2009; 457**:** 1061-70.

~~30~~34. Philips BJ, Redman J, Brennan A, Wood D, Holliman R, Baines D, *et al.* Glucose in bronchial aspirates increases the risk of respiratory MRSA in intubated patients. Thorax. 2005; 60**:** 761-4.

~~31~~35. Man WH, de Steenhuijsen Piters WA, Bogaert D. The microbiota of the respiratory tract: gatekeeper to respiratory health. Nat Rev Microbiol. 2017; 15**:** 259-70.

~~32~~36. Ji P, Zhang Y, Wang J, Zhao F. MetaSort untangles metagenome assembly by reducing microbial community complexity. Nat Commun. 2017; 8**:** 14306.

~~33~~37. Olm MR, Brown CA-O, Brooks B, Banfield JF. dRep: a tool for fast and accurate genomic comparisons that enables improved genome recovery from metagenomes through de-replication. ISME J. 2017; 11**:** 2864-68.

~~34~~38. Saraya T, Kurai D, Nakagaki K, Sasaki Y, Niwa S, Tsukagoshi H, *et al.* Novel aspects on the pathogenesis of Mycoplasma pneumoniae pneumonia and therapeutic implications. Front Microbiol. 2014; 5**:** 410.

~~35~~39. Floss HG, Yu TW. Rifamycin-mode of action, resistance, and biosynthesis. Chem Rev. 2005; 105**:** 621-32.

~~36~~40. Nesar S, MH. S, Rahim N, Rehman R. Emergence of resistance to fluoroquinolones among gram positive and gram negative clinical isolates. Pak J Pharm Sci. 2012; 25**:** 877-81.

~~37~~41. Axelsen PH. A chaotic pore model of polypeptide antibiotic action. Biophys J. 2008; 94**:** 1549-50.

~~38~~42. Harris M, Clark J, Coote N, Fletcher P, Harnden A, McKean M, *et al.* British Thoracic Society guidelines for the management of community acquired pneumonia in children: update 2011. Thorax. 2011; 66 Suppl 2**:** ii1-23.

Formatted: Font: Not Italic

~~39~~43. Bradley JS, Byington CL, Shah SS, Alverson B, Carter ER, Harrison C, *et al.* The management of community-acquired pneumonia in infants and children older than 3 months of age: clinical practice guidelines by the Pediatric Infectious Diseases Society and the Infectious Diseases Society of America. Clin Infect Dis. 2011; 53**:** e25-76.

~~40~~44. Lee H, Yun KW, Lee HJ, Choi EH. Antimicrobial therapy of macrolide-resistant Mycoplasma pneumoniae pneumonia in children. Expert Rev Anti Infect Ther. 2018; 16**:** 23-34.

~~41~~45. Hasegawa K, Mansbach JM, Ajami NJ, Espinola JA, Henke DM, Petrosino JF, *et al.* Association of nasopharyngeal microbiota profiles with bronchiolitis severity in infants hospitalised for bronchiolitis. Eur Respir J. 2016; 48**:** 1329-39.

~~42~~46. Hasegawa K, Linnemann RW, Mansbach JM, Ajami NJ, Espinola JA, Petrosino JF, *et al.* Nasal airway microbiota profile and severe bronchiolitis in infants: a case-control study. Pediatr Infect Dis J. 2017; 36**:** 1044-51.

~~43~~47. Citti C, Dordet-Frisoni E, Nouvel LX, Kuo CH, Baranowski E. Horizontal

Formatted: Font: Not Italic

gene transfers in Mycoplasmas (Mollicutes). Curr Issues Mol Biol. 2018; 29**:** 3-22.

~~44~~48. Xiao L, Ptacek T, Osborne JD, Crabb DM, Simmons WL, Lefkowitz EJ, *et al.* Comparative genome analysis of Mycoplasma pneumoniae. BMC Genomics. 2015; 16**:** 610.

~~45~~49. Rohde H, Qin J, Cui Y, Li D, Loman NJ, Hentschke M, *et al.* Open-source genomic analysis of Shiga-toxin-producing E. coli O104:H4. N Engl J Med. 2011; 365**:** 718-24.

~~46~~50. Davies MR, Holden MT, Coupland P, Chen JH, Venturini C, Barnett TC, *et al.* Emergence of scarlet fever Streptococcus pyogenes emm12 clones in Hong Kong is associated with toxin acquisition and multidrug resistance. Nat Genet. 2015; 47**:** 84-7.

~~47~~51. Kutty PK, Jain S, Taylor TH, Bramley AM, Diaz MH, Ampofo K, *et al.* Mycoplasma pneumoniae among children hospitalized with community-acquired pneumonia. Clin Infect Dis. 2019; 68**:** 5-12.

~~48~~52. Blyth CC, Gerber JS. Macrolides in children with community-acquired

Formatted: Font: Not Italic

Formatted: Font: Not Italic

Formatted: Font: Not Italic

Formatted: Font: Not Italic

Formatted: Font: Not Italic

pneumonia: panacea or placebo? J Pediatric Infect Dis Soc. 2018; 7**:** 71-77.

~~49~~53. Yang D, Chen L, Chen ZA-O. The timing of azithromycin treatment is not associated with the clinical prognosis of childhood Mycoplasma pneumoniae pneumonia in high macrolide-resistant prevalence settings. PLoS One. 2018; 13**:** e0191951.

~~50~~54. Larsen JM, Musavian HS, Butt TM, Ingvorsen C, Thysen AH, Brix S. Chronic obstructive pulmonary disease and asthma-associated Proteobacteria, but not commensal Prevotella spp., promote Toll-like receptor 2-independent lung inflammation and pathology. Immunology. 2015; 144**:** 333-42.

~~51~~55. Segal LN, Clemente JC, Tsay JC, Koralov SB, Keller BC, Wu BG, *et al.* Enrichment of the lung microbiome with oral taxa is associated with lung inflammation of a Th17 phenotype. Nat Microbiol. 2016; 1**:** 16031.

~~52~~56. de Dios Caballero J, Vida R, Cobo M, Maiz L, Suarez L, Galeano J, *et al.* Individual patterns of complexity in cystic fibrosis lung microbiota, including predator bacteria, over a 1-year period. MBio. 2017; 8**:** e00959-17.

~~53~~57. Maier L, Pruteanu M, Kuhn M, Zeller G, Telzerow A, Anderson EE, *et al.*

Formatted: Font: Not Italic

Formatted: Font: Not Italic

Extensive impact of non-antibiotic drugs on human gut bacteria. Nature. 2018; 555**:** 623-28.

~~54~~58. Qin J, Li R, Raes J, Arumugam M, Burgdorf KS, Manichanh C, *et al.* A human gut microbial gene catalogue established by metagenomic sequencing. Nature. 2010; 464**:** 59-65.

59. Lloyd-Price J, Mahurkar A, Rahnavard G, Crabtree J, Orvis J, Hall AB, *et al.* Strains, functions and dynamics in the expanded Human Microbiome Project. Nature. 2017; 550**:** 61-66.

60. Yan L, Yang M, Guo H, Yang L, Wu J, Li R, *et al.* Single-cell RNA-Seq profiling of human preimplantation embryos and embryonic stem cells. Nat Struct Mol Biol. 2013; 20**:** 1131-9.

~~55~~61. Luo R, Liu B, Xie Y, Li Z, Huang W, Yuan J, *et al.* SOAPdenovo2: an empirically improved memory-efficient short-read de novo assembler. Gigascience. 2012; 1**:** 18.

~~56~~62. Zhu W, Lomsadze A, Borodovsky M. Ab initio gene identification in metagenomic sequences. Nucleic Acids Res. 2010; 38**:** e132.

Formatted: Font: Not Italic

~~57~~63. Delcher AL, Bratke KA, Powers EC, Salzberg SL. Identifying bacterial genes and endosymbiont DNA with Glimmer. Bioinformatics. 2007; 23**:** 673-9.

~~58~~64. Li W, Godzik A. Cd-hit: a fast program for clustering and comparing large sets of protein or nucleotide sequences. Bioinformatics. 2006; 22**:** 1658-9.

~~59~~65. Enright AJ, Van Dongen S, Ouzounis CA. An efficient algorithm for large-scale detection of protein families. Nucleic Acids Res. 2002; 30**:** 1575-84.

~~60~~66. Chao A. Estimating the population size for capture-recapture data with unequal catchability. Biometrics. 1987; 43**:** 783-91.

~~61~~67. Nielsen HB, Almeida M, Juncker AS, Rasmussen S, Li J, Sunagawa S*, et al.* Identification and assembly of genomes and genetic elements in complex metagenomic samples without using reference genomes. Nat Biotechnol. 2014; 32**:** 822-8.

~~62~~68. Shannon P, Markiel A, Ozier O, Baliga NS, Wang JT, Ramage D, *et al.* Cytoscape: a software environment for integrated models of biomolecular interaction networks. Genome Res. 2003; 13**:** 2498-504.

6369. Zerbino DR, Birney E. Velvet: algorithms for de novo short read assembly using de Bruijn graphs. Genome Res. 2008; 18**:** 821-9.

6470. Bowers RM, Kyrpides NC, Stepanauskas R. Minimum information about a single amplified genome (MISAG) and a metagenome-assembled genome (MIMAG) of bacteria and archaea. 2017; 35**:** 725-31.

71. Parks DH, Chuvochina M, Waite DW, Rinke C, Skarshewski A, Chaumeil PA, *et al.* A standardized bacterial taxonomy based on genome phylogeny substantially revises the tree of life. Nat Biotechnol. 2018; 36**:** 996-1004.

72. Zhang C, Yin A, Li H, Wang R, Wu G, Shen J, *et al.* Dietary modulation of gut microbiota contributes to alleviation of both genetic and simple obesity in children. EBioMedicine. 2015; 2**:** 968-84.

65. Kurtz S, Phillippy A, Delcher AL, Smoot M, Shumway M, Antonescu C, *et al.* Versatile and open software for comparing large genomes. Genome Biol. 2004; 5**:** R12.

66. Jia B, Raphenya AR, Alcock B, Waglechner N, Guo P, Tsang KK, *et al.* CARD 2017: expansion and model-centric curation of the comprehensive antibiotic

Formatted: Font: Not Italic

resistance database. Nucleic Acids Res. 2017; 45**:** D566-D73.

~~67~~73. Chen L, Zheng D, Liu B, Yang J, Jin Q. VFDB 2016: hierarchical and refined dataset for big data analysis--10 years on. Nucleic Acids Res. 2016; 44**:** D694-7.

74. Ji M, Lee NS, Oh JM, Jo JY, Choi EH, Yoo SJ, *et al.* Single-nucleotide polymorphism PCR for the detection of Mycoplasma pneumoniae and determination of macrolide resistance in respiratory samples. J Microbiol Methods. 2014; 102**:** 32-6.

Tables

Table 1. Sample information

—	**Pneumonia Patients (n=76)**	**Healthy Children (n=171)**
**Characteristics**	—	—
Gender		
Female	24	87
Male	52	84
Age (years)	2.9(0.2-12.7)	4.3(0.1-8.9)
Sampling Site		
OP	75	171
NP	42	-
Lung	46	-
Delivery Mode		

Vaginally born	46	102
Cesarean section	30	69
Feeding Pattern		
Breast	48	84
Breast+Milk	12	66
Milk feed	16	21
Family history of allergy	1	-
History of pneumonia	14	-
Asthma	-	-
**Clinical symptoms**	—	—
Lung consolidation, atelectasis, infiltration	76	NA
Fever	44	-
Cough	72	-
Wheezing	20	-
Hospitalization time (days)	9(2-37)	-
CRP(<0.499mg/l)	22	NA
PCT(<0.5ng/ml)	73	NA
Eosinophils(0.5–5%)	44	NA

"-" represents no detection result; "NA" represents not available; CRP, C-response protein; PCT, procalcitonin

Formatted: Centered

Figure Legends

Figure 1. **Construction of the human RMGC**. Genome assembly was performed for each sample with ⩾650 Mbp of data. For samples with <650 Mbp of data, the data from the same respiratory site (NP, OP or the lung) were mixed and assembled. Gene predictions were conducted for all assembled contigs with ⩾500 bp and respiratory bacterial genomes in IMG. Genes with ⩾100 bp were retained. Respiratory gene sets

in HMP and PARTIC were combined to construct the non-redundant RMGC containing 2,245,343 genes.

Figure 2. Rarefaction curves for genes and KOs/gene families. a, Rarefaction curve for the gene count. **b,** Rarefaction curve for Chao2. The RMGC captured 90.52% of the prevalent genes. **c,** Rarefaction curve for KOs/gene families. Known functions saturate quickly to 6,346 groups. After including novel gene families, the rarefaction curve plateaus when 12,924 groups are detected. Boxes represent the interquartile ranges (IQRs) between the first and third quartiles, and the line inside the box represents the median value. Whiskers represent the lowest or highest values within values 1.5 times the IQR from the first or third quartiles. Circles represent data points located outside of the whiskers.

Figure 3. Core microbial species in healthy OP microbiota. The barplot on the top represent the prevalence of each core species, boxplot beneath the barplot means the relative abundance of each core species. The specific color stands for different phylum.

Figure 4. Differentiation of OP microbial samples between healthy children and

MPP patients. a, Gene counts in the OP microbiomes of healthy children and children with pneumonia. **b,** Alpha diversity of the OP microbiome in healthy children and children with pneumonia. Boxes represent the IQRs between the first and third quartiles, and the line inside the box represents the median. Whiskers represent the lowest or highest values within values 1.5 times the IQR from the first or third quartiles. Points represent data located outside of the whiskers. *** represents p-value $\leqslant$0.001.

Figure 5. Phylogenetic and functional alterations in children with pneumonia. a, Size of the circle represents the average relative abundance of CAGs in healthy children or children with pneumonia. A line between two circles indicates a Spearman's rank correlation coefficient $\geqslant$0.6 and an adjusted p-value $\leqslant$0.05. The phylum and genus corresponding to each CAG are indicated by the information listed on the left. **b,** The X-axis represents level-2 functional categories in KEGG, and the colour of the characters represents level-1 functional categories, which are listed on the right. The Y-axis shows the relative abundance of level-2 functional categories. *,** and *** represent adjusted p-value $\leqslant$0.05, $\leqslant$0.01 and $\leqslant$0.001, respectively.

Figure 6. Virulence-factor genes (VFGs) and antibiotic-resistance genes (ARGs) on *Mycoplasma pneumoniae* genome. The tracks from outside to inside represent ARGs, genes on plus strand, genes on negative strand and GC skew, respectively. VFGs painted with different colours refer to the different types of VFGs.

Figure 7. Comparison of relative abundance of 14 re-assembled genomes between healthy children and MPP patients. The blue circles and red triangles represent the microbial relative abundance of healthy children and MPP patients**.** Solid dot and paired whiskers represent the mean and SD of each microbial relative abundance. *, ** and *** represents p-value $\leqslant 0.05$, $\leqslant 0.01$ and $\leqslant 0.001$, respectively. NS stands for no statistical significance.

Figure 1 Click here to access/download;Figure;Figure 1.pdf

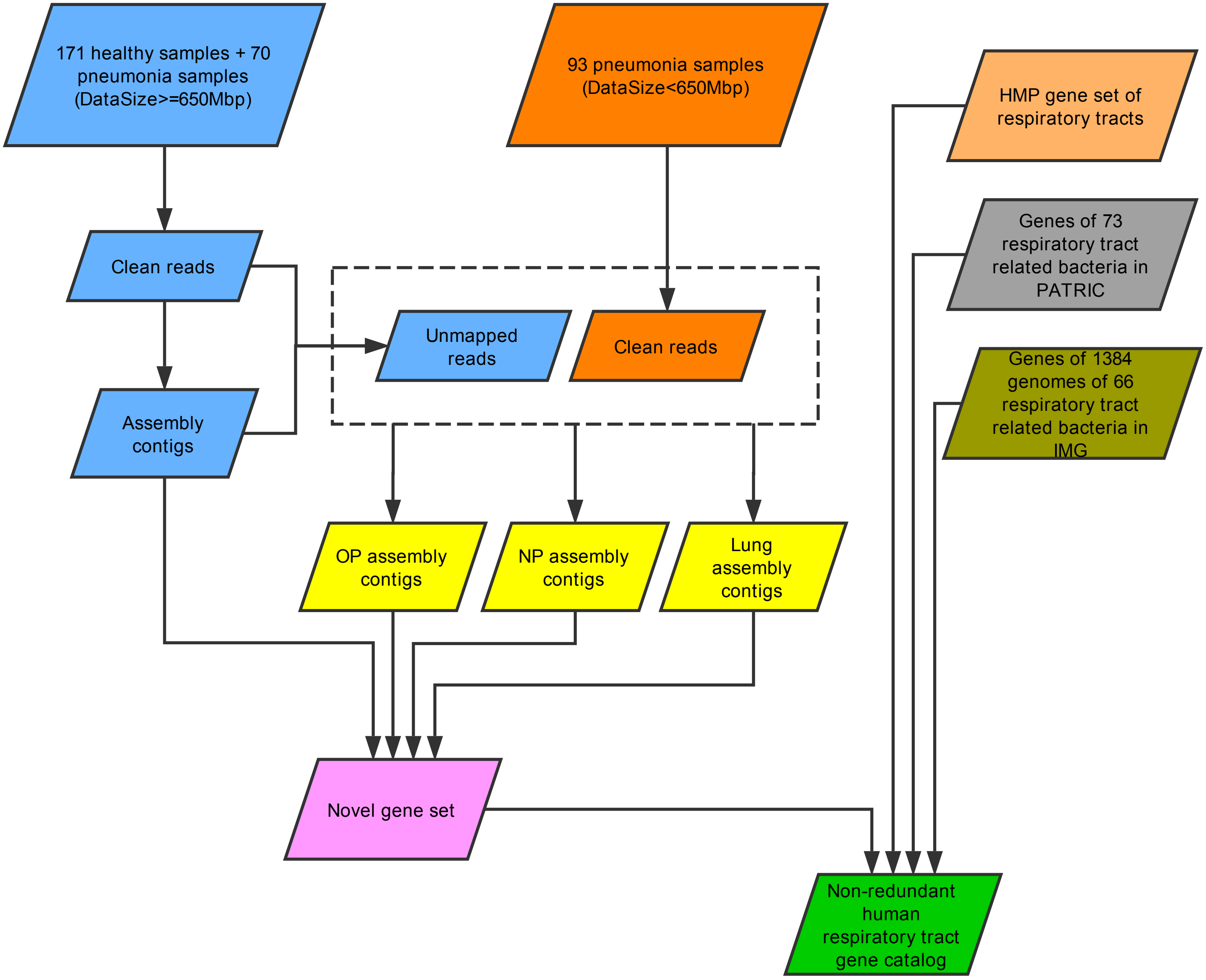

Figure 2 Click here to access/download;Figure;Figure 2.pdf

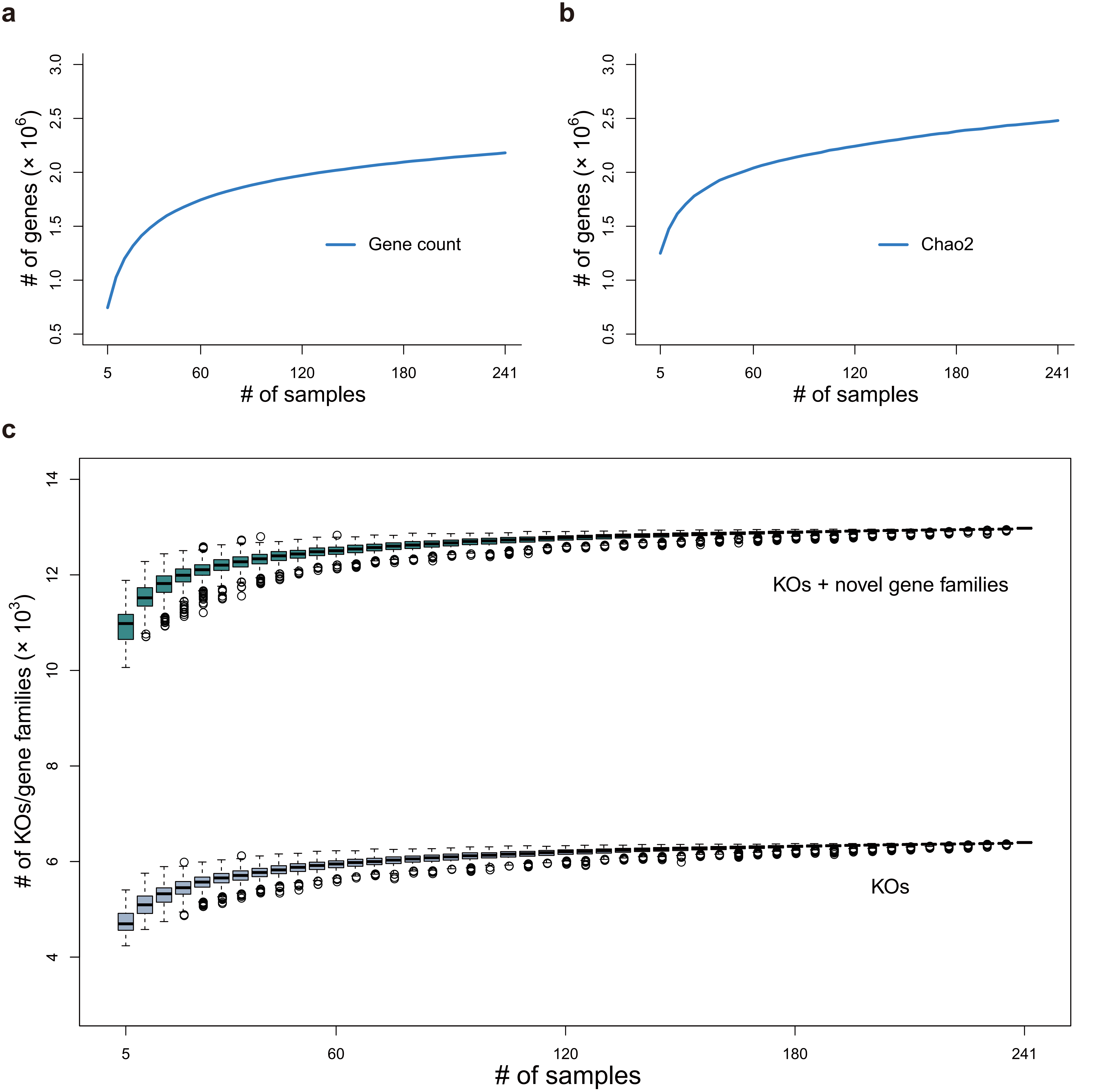

Figure 3

Click here to access/download;Figure;Figure 3.pdf

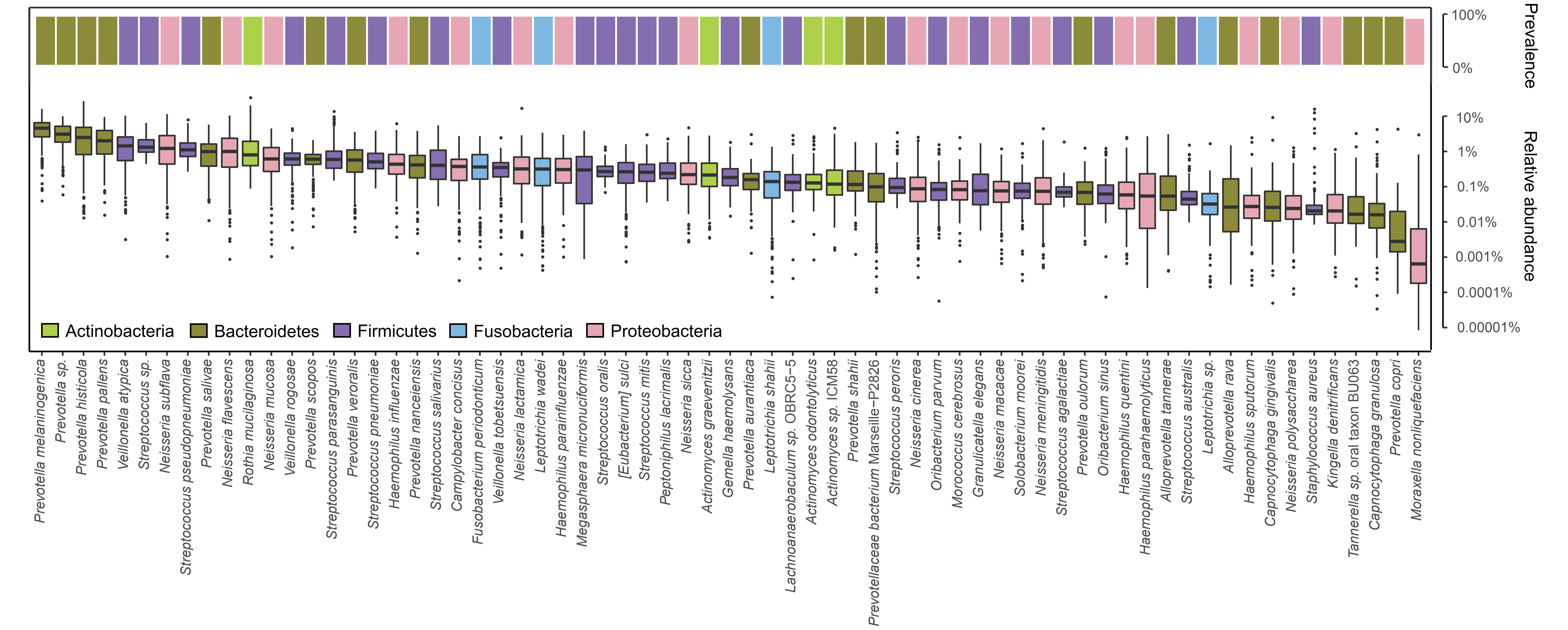

Figure 4

Click here to access/download;Figure;Figure 4.pdf

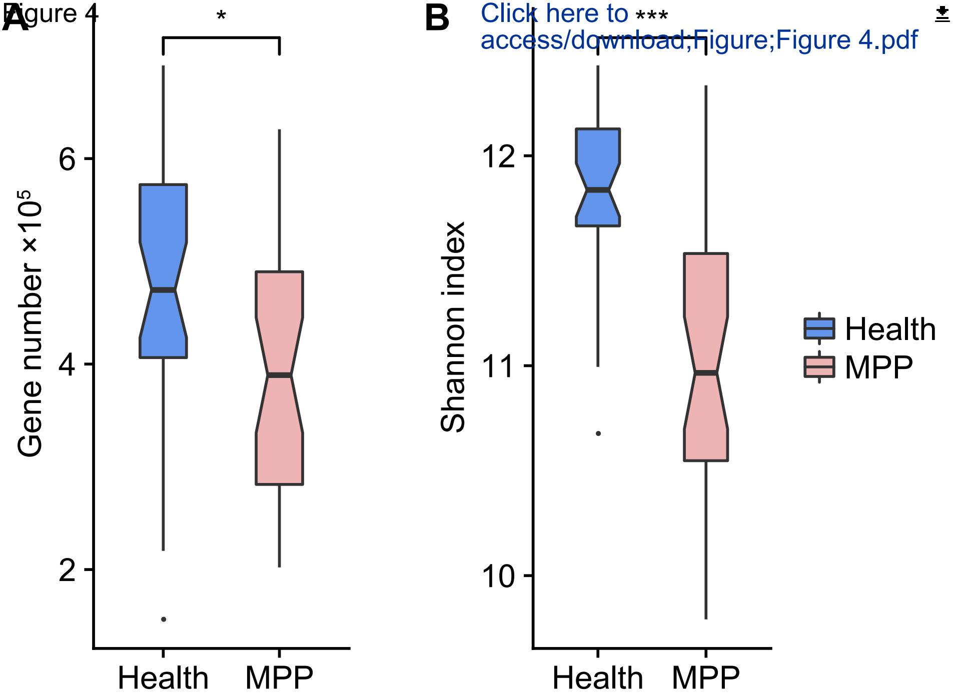

Figure 5

Click here to access/download;Figure;Figure 5.pdf

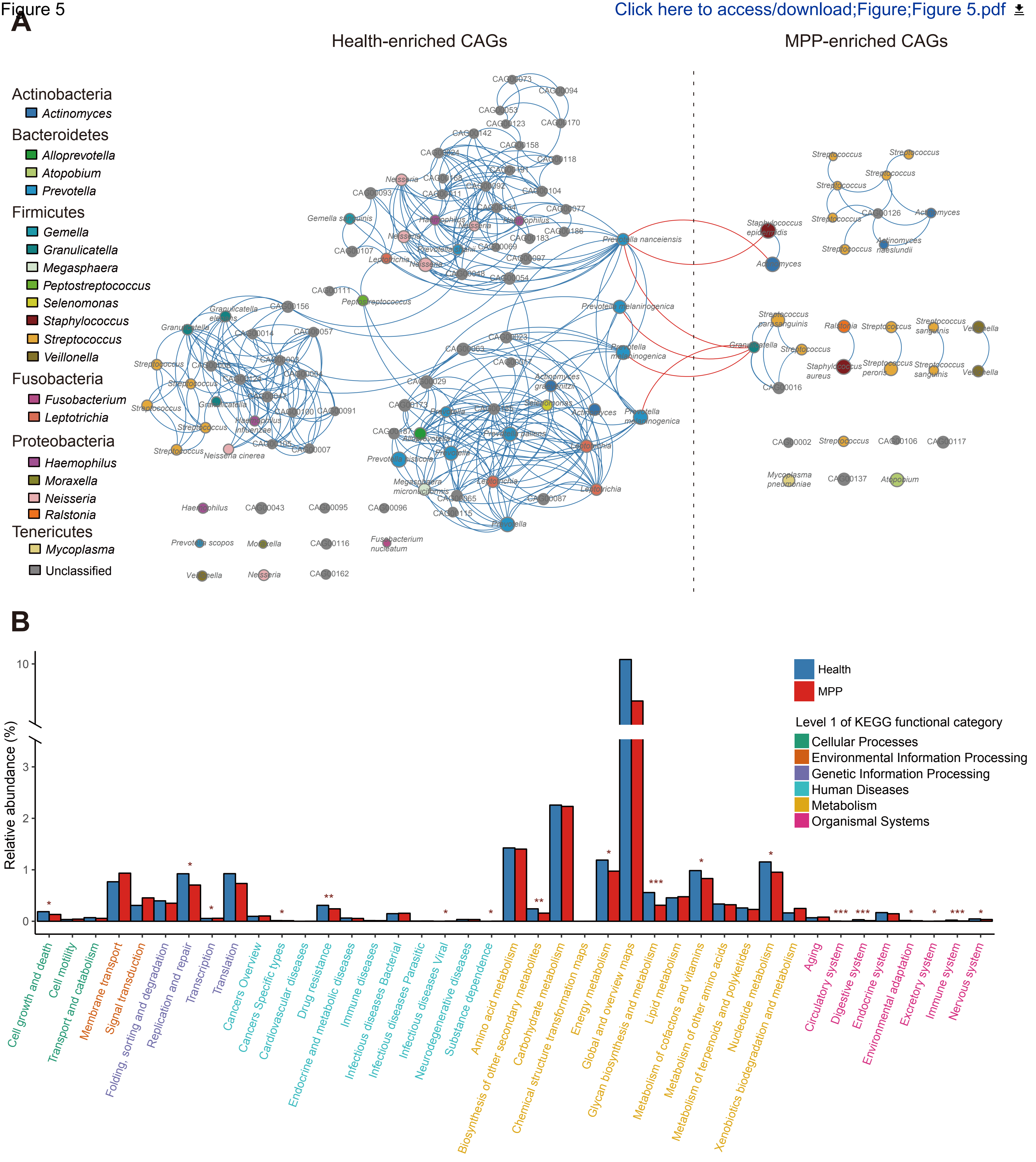

Figure 6

Click here to access/download;Figure;Figure 6.pdf

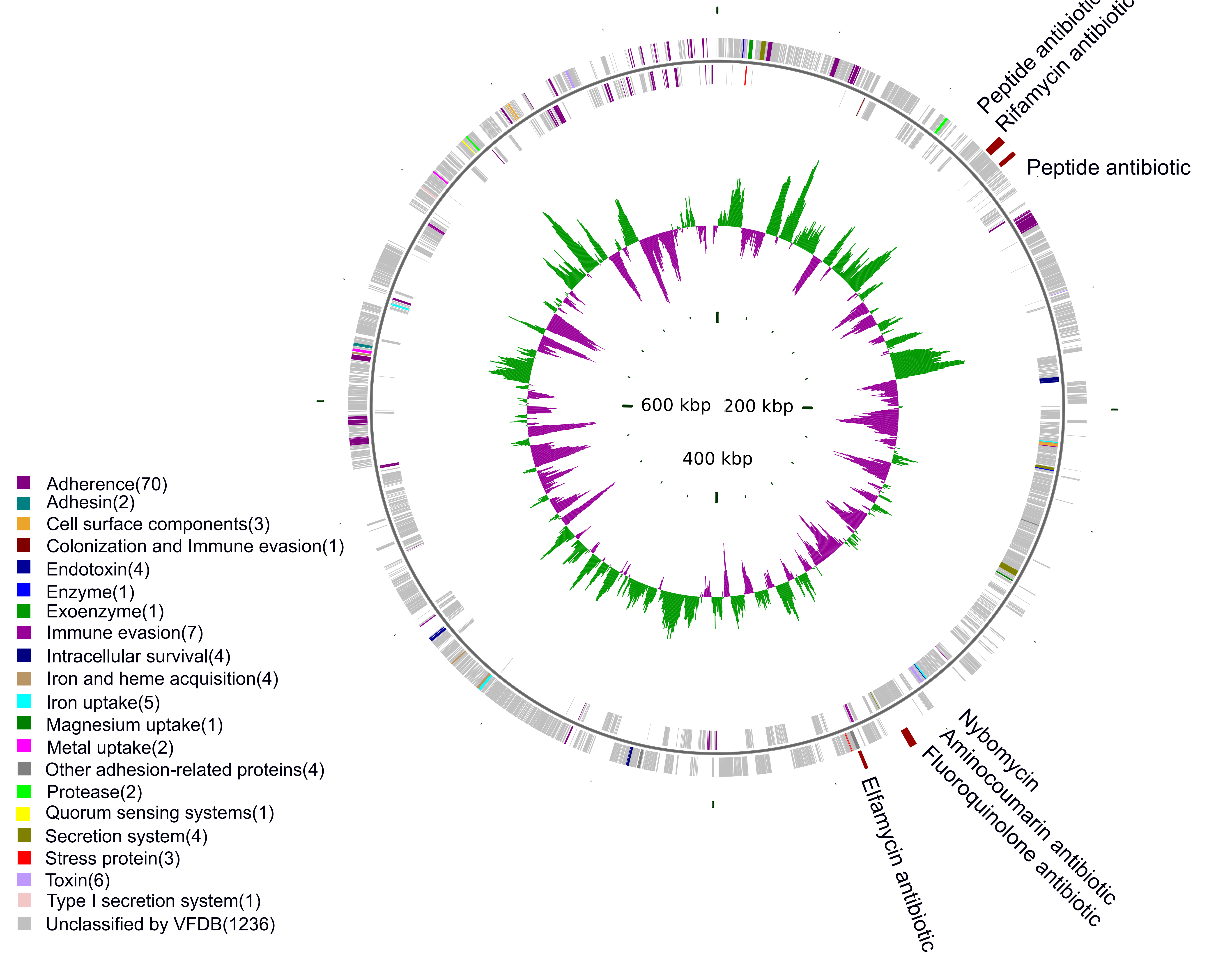

Figure 7

Click here to access/download;Figure;Figure 7.pdf

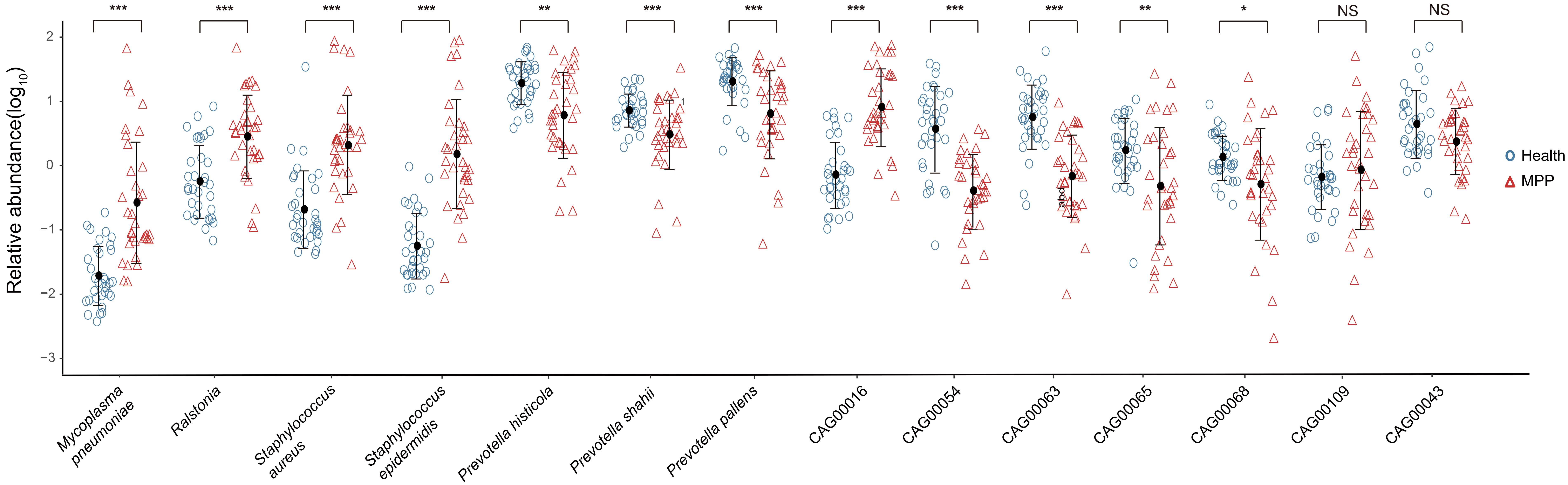

Supplemental Material Legends

Click here to access/download
Supplementary Material
Supplemental material legends.docx

Supplementary Figure 1

Click here to access/download
Supplementary Material
Supplementary Figure 1.pdf

Supplementary Figure 2

Click here to access/download
Supplementary Material
Supplementary Figure 2.pdf

Supplementary Figure 3

Click here to access/download
Supplementary Material
Supplementary Figure 3.pdf

Supplementary Table 1

Click here to access/download
Supplementary Material
Supplementary Table 1.xlsx

Supplementary Table 2

Click here to access/download
Supplementary Material
Supplementary Table 2.xlsx

Supplementary Table 3

Click here to access/download
Supplementary Material
Supplementary Table 3.xlsx

Supplementary Table 4

Click here to access/download
Supplementary Material
Supplementary Table 4.xlsx

Supplementary Table 5

Click here to access/download
Supplementary Material
Supplementary Table 5.xlsx

Supplementary Table 6

Click here to access/download
Supplementary Material
Supplementary Table 6.xlsx
