# Supplementary material for: An integrated respiratory microbial gene catalogue to better understand the microbial aetiology of Mycoplasma pneumoniae pneumonia
Source: Gigascience. 2019 Jul 31;8(8):giz093. doi: 10.1093/gigascience/giz093 (PMC6669060; doi:10.1093/gigascience/giz093)
Supplement: giz093_GIGA-D-19-00029_Revision_2 [file giz093_giga-d-19-00029_revision_2.pdf]

GigaScience

The integrated respiratory microbial gene catalogue facilitate the understanding of microbial aetiology in Mycoplasma pneumoniae pneumonia

--Manuscript Draft--

**Manuscript Number:**	GIGA-D-19-00029R2
**Full Title:**	The integrated respiratory microbial gene catalogue facilitate the understanding of microbial aetiology in Mycoplasma pneumoniae pneumonia
**Article Type:**	Research
**Funding Information:**	Sanming Project of Medicine in Shenzhen (SZSM201512030) — Dr Yuejie Zheng<br>Shenzhen Science and Technology Project (JCYJ20170303155012371) — Mr Heping Wang<br>Shenzhen Science and Technology Project (JCYJ20170816170527583) — Mr Heping Wang<br>Key Medical Disciplines Building Project of Shenzhen (SZXJ2017005) — Mr Zhiwei Lu

Abstract:

Background
An imbalanced respiratory microbiota has been observed in pneumonia which caused high morbidity and mortality in childhood. Respiratory metagenomic analysis demands a comprehensive microbial gene catalogue which will significantly advance our understanding of host-microbiota interactions.

Results
In this study, we collected 334 respiratory microbial samples from 171 healthy children and 76 pneumonia children. The established respiratory microbial gene catalogue (RMGC) comprised 2.25 million non-redundant microbial genes covering 90.52% prevalent genes. The core microbial species in the oropharynx (OP) of the healthy children mainly comprised Prevotella and Streptococcus. The OP microbial diversity and gene number in children with Mycoplasma pneumoniae pneumonia (MPP) decreased compared to that in healthy children, and the concurrence network of OP microbiota in patients is featured by Staphylococcus spp. and M. pneumoniae. Functional orthologues, which are associated with the metabolism of various lipids, membrane transport and signal transduction, accumulated in the OP microbiome of sick children. Several antibiotics-resistance genes (ARGs) and virulence-factor genes (VFGs) were identified in M. pneumoniae as well as other 13 microbial draft genomes, which were reconstructed via metagenomic data. Though the common macrolides/beta-lactam-resistance genes were not identified in assembled M. pneumoniae genome, a SNP mutation (A2063G) related with macrolides resistance was identified in 23S rRNA gene.

Conclusions
This study will facilitate exploring unknown microbial components and host-microbiota interaction in respiratory microbiome studies as well as render further insights into the microbial aetiology of MPP.

**Corresponding Author:**	Yuejie Zheng<br>Shenzhen Children's Hospital<br>Shenzhen, Guangdong CHINA
**Corresponding Author Secondary Information:**	
**Corresponding Author's Institution:**	Shenzhen Children's Hospital
**Corresponding Author's Secondary Institution:**	
**First Author:**	Wenkui Dai

Powered by Editorial Manager® and ProduXion Manager® from Aries Systems Corporation

**First Author Secondary Information:**	
**Order of Authors:**	Wenkui Dai
	Heping Wang
	Dongfang Li
	Qian Zhou
	Xin Feng
	Zhenyu Yang
	Wenjian Wang
	Chuangzhao Qiu
	Zhiwei Lu
	Ximing Xu
	Mengxuan Lyu
	Gan Xie
	Yinhu Li
	Yanmin Bao
	Yanhong Liu
	Kunling Shen
	Kaihu Yao
	Xikang Feng
	Yonghong Yang
	Shuaicheng Li
	Ke Zhou
	Yuejie Zheng
**Order of Authors Secondary Information:**	
**Response to Reviewers:**	Dear Editors and Reviewers,<br>Thanks for your comments. We have resubmitted the manuscript, please kindly check it.<br><br>1. Before we can proceed further you need to address the authorship issues we previously raised. i.e. that we only allow up to three joint-first authors and two corresponding authors. You can read more here:<br>https://doi.org/10.1093/gigascience/giy122<br>Please ensure all authors meet ICJME guidelines and have made an intellectual contribution to this paper, as if they haven't they should be moved to the acknowledgements:<br>http://www.icmje.org/recommendations/browse/roles-and-responsibilities/defining-the-role-of-authors-and-contributors.html<br>We require detailed authors contributions explaining all of this as well.<br><br>Response: We have resubmitted the revised manuscript without "Track Change" mode, and there are three joint-first authors and two corresponding authors in the revision. The authors who made an intellectual contribution were also mentioned in the "Authors' contributions" section. We hope this new submission could match your request.
**Additional Information:**	
**Question**	**Response**
Are you submitting this manuscript to a	No

Powered by Editorial Manager® and ProduXion Manager® from Aries Systems Corporation

special series or article collection?	
**Experimental design and statistics**<br><br>Full details of the experimental design and statistical methods used should be given in the Methods section, as detailed in our Minimum Standards Reporting Checklist. Information essential to interpreting the data presented should be made available in the figure legends.<br><br>Have you included all the information requested in your manuscript?	Yes
**Resources**<br><br>A description of all resources used, including antibodies, cell lines, animals and software tools, with enough information to allow them to be uniquely identified, should be included in the Methods section. Authors are strongly encouraged to cite Research Resource Identifiers (RRIDs) for antibodies, model organisms and tools, where possible.<br><br>Have you included the information requested as detailed in our Minimum Standards Reporting Checklist?	Yes
**Availability of data and materials**<br><br>All datasets and code on which the conclusions of the paper rely must be either included in your submission or deposited in publicly available repositories (where available and ethically appropriate), referencing such data using a unique identifier in the references and in the "Availability of Data and Materials" section of your manuscript.<br><br>Have you have met the above requirement as detailed in our Minimum Standards Reporting Checklist?	Yes

Powered by Editorial Manager® and ProduXion Manager® from Aries Systems Corporation

Manuscript

Click here to access/download;Manuscript;Manuscript-revison.docx

Click here to view linked References

The integrated respiratory microbial gene catalogue facilitate the understanding of microbial aetiology in *Mycoplasma pneumoniae* pneumonia

Running Title: Airway microbial gene set and altered microbiome

Wenkui Dai*, Department of Computer Science, City University of Hong Kong, Hong Kong 999077, China; daiwenkui84@gmail.com

Heping Wang*, Department of Respiratory Diseases, Shenzhen Children's Hospital, Shenzhen 518026, China; szetgmy@163.com

Dongfang Li*, Wuhan National Laboratory for Optoelectronics, Huazhong University of Science and Technology, No. 1037 Luoyu Road, Wuhan 430074, China; loveli_biocc@163.com

Qian Zhou, Department of Microbial Research, WeHealthGene Institute, Shenzhen 518000, China; zhouqian@wehealthgene.com

Xin Feng, Department of Microbial Research, WeHealthGene Institute, Shenzhen 518000, China; fengxin@wehealthgene.com

Zhenyu Yang, Department of Microbial Research, WeHealthGene Institute, Shenzhen 518000, China; yangzhy@wehealthgene.com

Wenjian Wang, Department of Respiratory Diseases, Shenzhen Children's Hospital, Shenzhen 518026, China; dhbk2005@163.com

Chuangzhao Qiu, Department of Microbial Research, WeHealthGene Institute, Shenzhen 518000, China; qiuchzh@wehealthgene.com

Zhiwei Lu, Department of Respiratory Diseases, Shenzhen Children's Hospital, Shenzhen 518026, China; luzhiwei1950@163.com

Ximing Xu, Institute of Statistics, Nankai University, No. 94 Weijin Road, Tianjin 300071, China; ximing@nankai.edu.cn

Mengxuan Lyu, Department of Computer Science, City University of Hong Kong, Hong Kong 999077, China; mengxualv2-c@my.cityu.edu.hk

Gan Xie, Department of Respiratory Diseases, Shenzhen Children's Hospital, Shenzhen 518026, China; xiegan1987@163.com

Yinhu Li, Department of Microbial Research, WeHealthGene Institute, Shenzhen 518000, China; liyh@wehealthgene.com

Yanmin Bao, Department of Respiratory Diseases, Shenzhen Children's Hospital, Shenzhen 518026, China; baoyanming1978@163.com

Yanhong Liu, Department of Microbial Research, WeHealthGene Institute, Shenzhen 518000, China; liuyanhong@wehealthgene.com

Kunling Shen, Department of Respiratory Diseases, Beijing Children's Hospital, Beijing 100045, China; Department of Respiratory Diseases, Shenzhen Children's Hospital, Shenzhen 518026, China; kunlingshen1717@163.com

Kaihu Yao, Department of Respiratory Diseases, Beijing Children's Hospital, Beijing 100045, China; Department of Respiratory Diseases, Shenzhen Children's Hospital, Shenzhen 518026, China; jiuhu2655@sina.com

Xikang Feng, Department of Computer Science, City University of Hong Kong, Hong Kong 999077, China; xikangfeng2-c@my.cityu.edu.hk

Yonghong Yang, Department of Respiratory Diseases, Beijing Children's Hospital, Beijing 100045, China; Department of Respiratory Diseases, Shenzhen Children's Hospital, Shenzhen 518026, China; Department of Microbial Research, WeHealthGene Institute, Shenzhen 518000, China; yyh628628@sina.com

Shuaicheng Li, Department of Computer Science, City University of Hong Kong, Hong Kong 999077, China; shuaicli@cityu.edu.hk

Ke Zhou#, Wuhan National Laboratory for Optoelectronics, Huazhong University of Science and Technology, No. 1037 Luoyu Road, Wuhan 430074, China; k.zhou@hust.edu.cn

Yuejie Zheng# Department of Respiratory Diseases, Shenzhen Children's Hospital, Shenzhen 518026, China; shine1990@sina.com

*These authors contributed equally to this work.

#Corresponding authors

Abstract

Background: An imbalanced respiratory microbiota has been observed in pneumonia which caused high morbidity and mortality in childhood. Respiratory metagenomic analysis demands a comprehensive microbial gene catalogue which will significantly advance our understanding of host-microbiota interactions. **Results:** In this study, we collected 334 respiratory microbial samples from 171 healthy children and 76 pneumonia children. The established respiratory microbial gene catalogue (RMGC) comprised 2.25 million non-redundant microbial genes covering 90.52% prevalent genes. The core microbial species in the oropharynx (OP) of the healthy children

mainly comprised *Prevotella* and *Streptococcus*. The OP microbial diversity and gene number in children with *Mycoplasma pneumoniae* pneumonia (MPP) decreased compared to that in healthy children, and the concurrence network of OP microbiota in patients is featured by *Staphylococcus spp.* and *M. pneumoniae*. Functional orthologues, which are associated with the metabolism of various lipids, membrane transport and signal transduction, accumulated in the OP microbiome of sick children. Several antibiotics-resistance genes (ARGs) and virulence-factor genes (VFGs) were identified in *M. pneumoniae* as well as other 13 microbial draft genomes, which were reconstructed via metagenomic data. Though the common macrolides/beta-lactam-resistance genes were not identified in assembled *M. pneumoniae* genome, a SNP mutation (A2063G) related with macrolides resistance was identified in 23S rRNA gene. **Conclusions:** This study will facilitate exploring unknown microbial components and host-microbiota interaction in respiratory microbiome studies as well as render further insights into the microbial aetiology of MPP.

Keywords

Pneumonia; *Mycoplasma pneumoniae*; Oropharynx; Microbiome; Respiratory microbial gene catalogue

Background

Studies have identified the indispensable respiratory microbiota[1-5] and its imbalance in pneumonia[6, 7], which is a leading cause of high morbidity and mortality[8, 9] worldwide, especially in children under 5 years[10, 11]. In recent years, *Mycoplasma pneumoniae* pneumonia (MPP) represents increasing cases in Chinese children[12] and microbial aetiology remains to be explored. Our previous studies unravelled altered respiratory microbiota in children with MPP[13, 14].

However, current respiratory microbiome (RM) studies have mainly focused on 16S rRNA analysis[6, 7, 15, 16] which merely provides cues about known bacterial components at the genus level. Emerging studies that applied a 16S rRNA analysis have revealed the imbalanced microbial structure in the respiratory tracts of children with pneumonia[7, 17, 18], but changes in the microbial functions and species-level microbial components in the RM of patients with MPP remain unexplored. In addition, current multi-omics studies are limited to explorations of known bacterial genomes in

the RM[15]. Nevertheless, the RM includes a high proportion of unknown microbial species[1-3, 5, 6] which require further exploration.

A comprehensive catalogue of reference genes is crucial for in-depth functional metagenomic analysis such as species/gene profiling, microbial biomarkers and functional annotation. Given that the RM varies with the environment[19], age[1, 2, 4] and disease[6, 7, 15, 16], we selected the nasopharynx (NP), oropharynx (OP) and lung samples from 76 children with pneumonia and OP samples from 171 healthy children in China to establish an integrated RMGC and study the imbalanced RM in Chinese children with MPP. Using this catalogue, we assessed the microbial components and functions in the OP microbiome of healthy and MPP children as well as the characteristics of recovered microbial genomes.

Data Description

From 3 July to 27 August 2016, patients were recruited from the hospitalization zone in the Department of Respiratory Diseases of Shenzhen Children's Hospital. Inclusion criteria for patients consisted of characteristic chest radiographic abnormalities consistent with pneumonia, the exclusion of asthma, and the clearance of respiratory

infections or exposure to antibiotics within one month prior to sampling (Table 1). We collected NP (25-800-A-50, Puritan, Guilford, ME, USA) and OP (155C, COPAN, Murrieta, CA, USA) swabs from 76 hospitalized patients within 24 hours after hospitalization and before the administration of antibiotics. Bronchoalveolar lavage fluids (BALFs) were collected 2 to 15 days after hospitalization (Supplementary Table 1).

Healthy children were recruited during physical examination in summer of 2016 (from July to August) in Shenzhen. OP swabs were collected from 171 healthy children who met the following inclusion criteria: no diagnosis of asthma or a family history of allergy; no history of pneumonia; a lack of wheezing, fever, cough or other respiratory/allergic symptoms at sampling one month prior to the study and one week after sampling; no exposure to antibiotics one month prior to sampling.

All samples were collected by an experienced clinician. Samples were stored at −80°C within 20 minutes after collection and DNA was extracted within 10 days of the sampling. A TGuide S32 Magnetic Swab Genomic DNA Kit (DP603-T2, TIANGEN Biotech (Beijing) Co., Ltd., Beijing, China, http://www.tiangen.com/en/)

was utilized to extract the DNA and metagenomic sequencing was performed on the Illumina Hi-Seq platform (San Diego, USA) in terms of the manufacturer's instructions. Unused swabs and DNA extraction kits from the same batch served as negative controls to assess DNA contamination.

Analyses

Sample information and data output

Two hundred forty-seven children aged <13 years were enrolled in this study (Table 1 and Supplementary Table 1). After removing host contamination and low-quality data, metagenomic sequencing produced 4,765,288,986 read numbers with an average of 14,267,332 read numbers per sample. DNA concentration of unused sampling swabs and DNA extraction kits was lower than 0.01 ng/μl, whereas the DNA concentration was higher than 80 ng/μl in sampling swabs and BALF. Furthermore, the DNA amplification results of extracted bacterial DNA were less than 0.01 nmol/l for the enveloped sampling or extraction materials (Supplementary Figure 1)

Construction of the RMGC

By applying metagenomics sequencing data from 247 children and three resources of respiratory related microbial genomes/genes (Figure 1), we constructed a comprehensive RMGC with 2,245,343 non-redundant ORFs and it was freely accessible through our website (https://rmgc.deepomics.org). The total length of the ORFs in the RMGC was 1.71 Gbp and the average length was 760 nt, ranging from 102 to 32,241 nt. We selected 241 samples with ⩾650 Mb data to examine the coverage of the microbial genes in the RMGC. In accordance with the rarefaction curve, 90.52% of prevalent microbial genes were captured in the RMGC (Figure 2a and b).

Taxonomic assessment and functional annotation of the RMGC

Based on taxonomic profiling, 1,281,673 genes (57.08% of RMGC) were assigned to phyla and 1,143,382 genes (50.92% of RMGC) were assigned to genera, representing 56.58% and 51.75% of the sequencing reads respectively. A total of 617,968 genes (25.92% of RMGC) were annotated to known bacterial species, representing 33.49% of the sequencing reads. The phyla Firmicutes, Bacteroides, Proteobacteria, Actinobacteria and Fusobacteria dominated the RMGC while the prevalent microbial

genera included *Staphylococcus*, *Streptococcus*, *Haemophilus*, *Corynebacterium*, *Dolosigranulum*, *Prevotella*, *Blautia*, *Rothia*, *Porphyromonas*, *Lactobacillus*, *Veillonella*, *Fusobacterium* and *Leptotrichia*. Unknown microbial species accounted for 9.62% to 55.50% of the RMGC and the detailed taxonomic information of RMGC was deposited on our website.

The genus-level microbial structure revealed by metagenomic analysis resembled the results of the 16S rRNA analysis (Supplementary Figure 2). Notably, a greater proportion of microbial genera remained unclassified in the metagenomic analysis than in the 16S rRNA analysis, which might be attributed to the wide detection by metagenomics sequencing and limited reference microbial genomes.

By aligning RMGC to KEGG database, a total number of 6,408 KEGG Orthology (KO) were identified, including 853,446 genes representing 37.85% of the total sequencing data. Known microbial functions (annotated by KEGG) saturated quickly to 6,346 groups as more samples were included (Figure 2c). Combined novel gene families, the rarefaction curve plateaued when 12,924 groups were detected (Figure 2c). Upon alignment to the eggNOG database, 53.95% of the genes in the

RMGC were assigned to known functional categories.

Core microbial species in OP microbiome of healthy children

We acquired 67 core species in total in 5 dominant phyla Bacteroidetes, Firmicutes, Proteobacteria, Actinobacteria and Fusobacteria (Figure 3). *Prevotella melaninogenica* (4.38±2.91%, mean±sd), *Prevotella sp.* (3.06±1.92%), *Prevotella histicola* (3.23±3.58%), *Prevotella pallens* (2.31±1.88%) and *Veillonella atypical* (1.60±1.44%) were the top 5 microbial species. In addition, *Streptococcus pseudopneumoniae* (1.26±0.96%), *Haemophilus influenzae* (0.60±0.68%), *Streptococcus pneumoniae* (0.60±0.50%), *Haemophilus parainfluenzae* (0.42±0.49%) and *Staphylococcus aureus* (0.27±1.52%), which were generally defined as opportunistic pathogens, were also prevalent in OP microbiome of healthy children (Figure 3).

Microbial structure and functions in OP microbiome of MPP patients differed from that in healthy children

Based on the PERMANOVA, pneumonia onset is the most significant factor (adjust *p*-value <0.001) explaining the variations in OP microbial samples, followed by feed

pattern (adjust *p*-value = 0.037) and age (adjust *p*-value = 0.048). Compared with healthy children, MPP patients exhibited significantly decreased microbial gene number and alpha diversity of the OP microbiome (Figure 4a and b). Moreover, thirty Co-Abundance gene Groups (CAGs) accumulated significantly in the OP microbiome of MPP patients, comprising 6 unknown and 24 known microbial species which were primary respiratory pathogens such as *M. pneumoniae*, *Staphylococcus epidermidis* and *S. aureus* (Figure 5a). Ninety-five CAGs were enriched in the OP microbiome of healthy children including prevalent colonizers such as *Prevotella* species (Figure 5a). The microbial co-occurrence networks in MPP patients were simpler than that in healthy children and negative correlations were only identified between health-enriched and MPP-enriched CAGs (Figure 5a). For example, health-enriched *Prevotella spp.* were negatively correlated with MPP-enriched *S. epidermidis* ($r<-0.60$, adjust p-value $\leqslant 0.05$, Figure 5a).

By comparing functional annotations via KEGG annotation (Supplementary Table 2), we assessed the functional alterations of the OP microbiome in patients with MPP. Microbial functions which related to lipid metabolism, membrane transport and

signal transduction were slightly enriched in MPP patients (Figure 5b). In contrast, the OP microbiome of healthy children was significantly enriched in orthologous involved in glycan biosynthesis and metabolism, biosynthesis of secondary metabolites, and cell growth and death (Figure 5b and Supplementary Table 2). Host homeostatic associated functions, such as immune system, digestive system, circulatory system and environmental adaptation were also significantly abundant in the OP microbiome of healthy children (Figure 5b and Supplementary Table 2).

Characterization of the *M. pneumoniae* genome and other 13 re-constructed microbial genomes

We re-assembled 14 qualified microbial CAGs (Supplementary Table 3) which represented *M. pneumoniae* genome (0.80 Mbp) and 13 other microbial genomes (genome sizes averaged 2.30 Mbp). The *M. pneumoniae* genome accumulated significantly in OP microbiome of MPP patients and exhibited high similarity with reference genome (97.79% of genome coverage) (Supplementary Table 3). *M. pneumoniae* genome consisted of 4 antibiotic-resistance genes (ARGs) with common antibiotics, including peptide, rifamycin and fluoroquinolone antibiotics (Figure 6,

Supplementary Table 4). On the other hand, SNP mutation A2063G related to macrolides-resistance was identified in 23S rRNA gene in 8 MPP patients, who were given experimental macrolides or beta-lactams such as azithromycin, erythromycin or sulbactam (Supplementary Table 1). In addition, there were 136 virulence-factor genes (VFGs) along its reassembled genome sequence (Supplementary Table 5) and the redundant VFGs of *M. pneumoniae* enriched in the secretion of adhesin P1, cytadherence protein and community-acquired respiratory distress syndrome (CARDS) toxin (Figure 6 and Supplementary Table 5).

Among other 13 microbial genomes, 5 of them can be designated specific species, one just be annotated at genus level (*Ralstonia*) and the rest 7 were novel microbial genomes (averaged 1.74 Mbp) (Supplementary Table 3). For the 5 annotated microbial species, *S. aureus* and *S. epidermidis* increased significantly in MPP patients while the other 3 *Prevotella spp.* mainly accumulated in healthy children (Figure 7, Supplementary Table 3). The largest reassembled *Ralstonia* genome (5.89Mbp) carried numerous ARGs, including 13 beta-lactam antibiotic genes, 21 tetracycline antibiotic genes, and 11 macrolide antibiotic genes. *P. histicola*, *P. shahii*

and CAG00068 all had one copy of macrolide antibiotic resistance and beta-lactam antibiotic resistance gene. These genomes also harboured abundant resources of VFGs which ranged from 105 to 808 copies of relative genes. According to the correlation analysis, we didn’t identify the significant correlation between 14 reassembled microbial genomes and 5 clinical indexes (Supplementary Table 6).

Discussion

MPP has been causing the increasing morbidity and mortality in Chinese children. The development of RM studies has improved our understanding of the microbial aetiology of MPP by revealing infection-associated RM imbalances[13, 14]. However, microbial functions and host-microbiota interactions in the RM of patients with MPP remain to be explored, particularly those from novel microbial species.

In recent years, several reference gut microbial catalogues were constructed to promote understanding of the host-microbe interaction. Qin *et al.* built a global view of the human gut microbiome (GM) and revealed a comprehensive functional potential of the prevalent gut microbial genes[20]. Li *et al.* upgraded the gut gene catalogue in 2014[21], enabling the studies of association of the microbial genes with

human health. Based on these frameworks, researchers could deepen the understanding of the correlation between GM and various diseases, such as gastrointestinal and cardiovascular diseases[22, 23].

Similar to reference gene catalogues of the GM, RMGC will further understanding of microbial aetiology in respiratory diseases. The development of a well-established RMGC in this study is crucial for the functional metagenomics analysis to improve our knowledge about host-microbiota interactions in MPP. By aligning metagenomics data directly with the established RMGC, researchers could profile all microbial species as well as explore microbial functions in both known and unknown microbial species. The similar microbial assignment between RMGC-based and 16S rRNA analysis also suggested promising taxonomic assignments via our constructed gene sets. The core microbial species of OP microbiota in healthy children will provide a reference for exploring microbial as well as host-microbe interactions in RM study[24]. In general, RMGC furnishes a comprehensive respiratory associated microbial profile to forward the microbiome analysis at species level and the functional profiling will facilitate in-depth multi-omics analyses[25, 26], such as

associations of produced proteins or metabolites with known and novel microbial genomes. This capability would clarify the interactions between the host and the RM alteration during MPP progression.

The OP microbiome of MPP children changed to be simpler structure compared to that of healthy children. Previous studies revealed that bacteria-like *M. pneumoniae* could deplete bacteria through direct competition and activate the bacterial clearance factors in host responses[27, 28], which led to decreased colonizer *Prevotella spp.*[29] and pathogens proliferation such as *S. aureus* and *S. epidermidis*. The MPP patient-enriched gene functions involved in membrane transport and various nutrients metabolism which could partly explain reduced tight junction proteins and increased respiratory mucosa permeability after infection[30]. In addition, a number of studies have identified an increased glucose concentration in airway surface liquids[31-33] and associated pathogen proliferation[34], which also corroborate the enriched nutrients uptake pathways in OP microbiome of MPP patients. Though the mechanism of *M. pneumoniae* clearance in respiratory system remains unclear, these findings would render a new insight into host-microbiota interactions in MPP infection.

Except for well-known microbes, respiratory tracts also harboured a variety of undiscovered microbial species[35]. Moreover, recent reports had proved that single bacterial genome could be well recovered via reference gene sets and metagenomics data[36, 37]. Culturing of *M. pneumoniae* is rarely and difficultly used in clinical diagnosis, limiting the understanding of antibiotics resistance and virulence[38]in *M. pneumoniae*. Re-construction of the *M. pneumoniae* genome by employing RMGC and metagenomic data indicated various ARGs which were related to RNA transcription[39], DNA replication[40] and protein synthesis[41]. According to clinical practice guidelines[42-44] and ARGs existence, most of MPP children were treated with azithromycin, erythromycin or sulbactam which were not associated with identified ARGs in *M. pneumoniae* genome. Increasing reports demonstrated that the specific dominated bacteria associated with severe acute respiratory infections (ARIs)[6, 45, 46], but no meaningful correlations were identified between disease severity and *M. pneumoniae,* as well as other reassembled bacteria in OP microbiome of MPP patients. This was also identified by our previous studies which confirmed the succession of *M. pneumoniae* infection in NP to OP and lung as well as the association of *M.*

pneumoniae load in the lung microbiota with disease severity[14].

Though no macrolide/beta-lactam resistance genes were discovered in *M. pneumoniae* genome, one SNP mutation (23S RNA, 2063A->G) correlated to macrolide resistance were identified in MPP patients. Meanwhile, the patient-enriched microbial genomes such as *Ralstonia,* consisted plenty of ARGs related to the resistance to macrolide, beta-lactam and tetracycline. Given rigorous antibiotic selective pressure and complex microbial interaction, the environmental redundant genetic components would rapidly transferred into the pathogen genome by horizontal gene transfer[47, 48] and caused several emergence diseases, such as European enterohemorrhagic *Escherichia coli* breakout[49] and emergence of scarlet fever *Streptococcus pyogenes* in Hong Kong[50]. Considering above-mentioned researches, we should recognize that current medications for the *M. pneumoniae* treatment hold the potential to trigger emerging drug-resistance microbial species in *M. pneumoniae* or other novel microbial species, such as reported macrolide resistance in *M. pneumoniae*-PCR-positive children[51-53]. The OP microbiome also recovered several healthy enriched bacterial genomes, among which *Prevotella spp.* played as key

players in OP microbiome of healthy children[54, 55] and other novel microbes might function as pathogen competitors such as *Vampirovibrio*[56]. In general, recovered microbial genomes in respiratory tracts hold the potential to improve the understanding of microbial aetiology in MPP pneumonia.

There are several limitations to be clarified in this study. Given no efficient medicines for MPP, the inpatients accepted empirical treatments and might shift the airway ecology slightly[57]. Despite the promising application of the RMGC, unclassified CAGs and novel gene families in RMGC must be annotated and further explored. The copy numbers of several genes need further assessment due to potential inaccuracy caused by the low respiratory bacterial biomass, NGS sequencing and assembly methods. The respiratory microbial samples were obtained from Chinese children in this study, and more metagenomics data will be incorporated into the RMGC in the future to construct a broader characterization of microbial components and functions, as the continual updates of the GM reference genes[21, 58, 59]. This procedure will incrementally improve studies of the imbalanced RM in patients with respiratory diseases. Alterations in the OP microbiome in Chinese patients with MPP

will also provide extensive insights into the microbial aetiology of acute respiratory infection.

Potential implications

Established respiratory microbial gene catalogue will ensure deepen respiratory micro-ecology research, which holds the promise to elucidate respiratory microbial community at microbial species level. In addition, genomes of novel microbial genera or species can be assembled through aligning metagenomics data with the reference catalogue. Exploring microbial functions and associated microbial components can construct the microbial network in respiratory microbial community. Established reference gene sets can be employed to deepen multi-omics analysis, which will further the understanding of host-microbiota interactions in acute respiratory infection. Comparing oropharynx microbiome between healthy and diseased children also provides an example for the utilization of the gene sets.

Methods

Ethics statement

We obtained approval for this study from the Ethical Committee of Shenzhen Children's Hospital (Shenzhen, Guangdong Province, China) under registration number 2016013 and performed experiments under the relevant guidelines and regulations. All guardians of selected children provided the informed consents.

Clinical detection of infectious pathogens

BALF was employed to establish the common clinical microbial diagnosis. Culturing was conducted to detect *S. pneumoniae, H. influenzae*, *Moraxella catarrhalis*, *S. aureus* and *Staphylococcus haemolyticus*. The D3 Ultra DFA Respiratory Virus Screening & ID Kit (Diagnostic Hybrids, Inc., Athens, OH, USA) was employed to detect common viruses, including adenovirus (AdV), respiratory syncytial virus (RSV), influenza virus and parainfluenza virus. Cytomegalovirus (CMV) and Epstein-Barr virus (EBV) were detected via the Diagnostic Kit for Quantification of Human CMV DNA and EBV Polymerase Chain Reaction (PCR) Fluorescence Quantitative Diagnostic Kit, respectively (DaAnGene, Guangzhou, China, http://daan.joomcn.com/). *M. pneumoniae* and *Chlamydia pneumonia* were diagnosed via the diagnostic kit for *M. pneumoniae* DNA (PCR Fluorescence Probing) (DaAnGene) and Anti *C. pneumoniae* ELISA (IgM) (EUROIMMUN AG, Lübeck, Germany) respectively.

Construction and annotation of the RMGC

Sequencing data were filtered using a previously reported method[60] and each sample

with ⩾650 Mbp data (Figure 1, Supplementary Figure 3) was selected for genome assembly by SOAPdenovo [61](v2.07, –F –K 39 –M 3 –d 1). For samples with <650 Mbp data, the data from the same respiratory site were mixed and assembled (Figure 1). Assembled contigs with ⩾500 bps were selected for gene prediction with MetaGeneMark[62] (v3.26, default parameters). We applied Glimmer3.02[63] (default parameters) to predict genes from the 1,384 respiratory bacterial genomes in the IMG database (2016-12-21, https://img.jgi.doe.gov/). Gene sequences were also retrieved from the genomes of 73 respiratory bacteria in PATRIC database (2017-3-25, https://www.patricbrc.org/) and 450,204 open reading frames (ORFs) of respiratory bacteria in Human Microbiome Project (HMP). Genes with ⩾100 bp length and without Ns were selected to construct non-redundant gene sets using CD-HIT[64] (v4.66, -c 0.95 –aS 0.9). Genes with ⩾2 mapped reads were retained in the established RMGC.

The taxonomic annotation of genes was conducted in the light of the following steps: i) we retrieved bacterial and viral genome sequences from IMG (2016-12-21), NCBI (2016-08-09) and PATRIC (2017-03-25) databases. We selected the genome

sequence with the longest N50 as the representative genome for each bacterial species. Non-redundant viral genomes were produced by CD-HIT (v4.66, -aS 0.95 -aL 0.9 -M 0). We aligned the gene sets in the RMGC to 6,869 representative bacterial genomes and 18,916 non-redundant viral DNA genomes using BLASTN (v2.5.0, default parameters except –e 0.01); ii) we retained the top 10% highest-scoring alignments of each gene, with ⩾65% identity and ⩾80% coverage of gene length; and iii). The assignment of each gene was determined based on ⩾50% consensus above the similarity threshold for a specific rank: ⩾65% for phylum, ⩾85% for genus and ⩾ 95% for species.

The functional annotation of each gene was determined by searching protein sequences in Kyoto Encyclopedia of Genes and Genomes (KEGG) (v78.1) and eggNOG (version 4.0) with BLASTP (v2.5.0, default parameters, except -e 1e-5). The best-hit alignment (identity ⩾30% and coverage ⩾70%) was selected as the functional annotation for the gene. Genes without annotations in KEGG were identified as novel gene families by the Markov Cluster Algorithm (MCL)[65] (inflation factor=1.1, bit-score cut-off=60).

Comparing the taxonomic assessment between 16S rRNA gene analysis and metagenomic analysis

We selected 72 OP microbial samples with ⩾650 Mb metagenomic sequencing data and aligned the sequencing data to establish RMGC to determine taxonomic assignments. The same samples were also sequenced via V3-V4 region of the 16S rRNA gene[13]. Microbial compositions were compared between two methods to assess the accuracy of taxonomic assignments via metagenomic analysis.

Rarefaction analysis

We downsized the number of mapped reads to 3 million for each sample to eliminate the variable influence caused by the amount of sequencing data. Estimation of total gene richness was done by randomly sampling five individuals 1,000 times with gene counting and Chao2 richness estimator[66].

For the rarefaction curve of KEGG orthologous groups (KOs) and novel gene families, random sampling of five individuals for 1,000 times was used to evaluate saturation. Relative rarefaction curves were visualized using R software (v3.3.2).

Calculation of gene relative abundance in RMGC

All filtered reads of metagenomics data from each sample were aligned to the established RMGC using BWA (v0.7.13, default parameters, except for the mem and identity ⩾95%). Alignments that met the following two criteria were accepted: i) paired-end reads were mapped onto a same gene with the correct insert size; and ii) one end of a paired-reads was mapped onto the end of a gene, while the other was located outside of the gene.

If the number of genes in a given sample was n, the relative abundance was calculated using the following steps:

Step 1. The copy number of the gene *i* ($c(i)$) was calculated as:

$$c(i) = \frac{t(i)}{l(i)}$$

$t(i)$: The total number of mapped reads of gene *i* in a given sample.

$l(i)$: The length of the gene *i*.

Step 2. The relative abundance of gene *i* ($Ab\_g(i)$) was defined as:

$$Ab\_g(i) = \frac{c(i)}{\sum_{1}^{n} c(i)}$$

Step 3. If m genes can be assigned to the phylogenetic assignment s, the abundance of this phylogenetic assignment ($Ab\_p(s)$) was calculated using the

following equation:

$$Ab\_p(s)=\sum_{1}^{m} Ab\_g(j)$$

Phylogenetic and functional profile of the OP microbiome

All filtered reads of the OP microbiome were aligned to the established RMGC using BWA with same parameter as above. The relative abundance of each phylogenetic assignment was calculated as showed above while the abundance of KOs in the functional profiling table was determined as described in a previous report[58].

Identification of OP core microbial species in healthy children

The microbial species was selected as core species if it existed in over 50% of healthy children and represented more than 1% relative abundance in one OP microbial sample. The distributions of core microbial species in OP of healthy children were described using ggplot2 in R.

Comparison of the OP microbiome between healthy children and MPP patients

According to the age distributions of 34 MPP patients (data size ⩾650 Mbp), 33 randomized healthy children with similar age were chosen. Genes in the OP microbiome of selected microbial samples were clustered into co-abundance gene

groups (CAGs) via Capony-based algorithms[67] (default parameters). The selected CAGs which contained more than 700 genes were regarded as deriving from the same bacterial genome and selected to construct a correlation network using Spearman's rank coefficient (⩽−0.6 or ⩾0.6). The co-occurrence network was visualized using Cytoscape (v3.4.0)[68]. If ⩾50% of the included genes had consensus phylogenetic annotations, corresponding CAG was assigned to a related microbial taxonomic assignments.

The relative abundance of each CAG in microbial samples was calculated as previously reported[59]. Inter-group comparisons of CAGs and KEGG functions were performed using the two-tailed Wilcoxon rank-sum test and corrected via the Benjamini-Hochberg method (adjusted *p*-value ⩽ 0.05). Confounding factors including pneumonia, sex, age, delivery mode and feed pattern were also assessed using PERMANOVA by vegan package (v2.3-4) in R software.

Single microbial genome assembling from OP metagenomic data

OP metagenomic data were aligned to the filtered CAGs (containing ⩾700 genes) by BWA (v0.7.13, identity ⩾95%). The mapped reads of each CAG were extracted for

microbial genomes assembling with Velvet[69] (kmer: from 45 to 75, cov_cutoff: auto, exp_cov: auto). The assembled sequences with the longest contig N50 were selected as representative draft genomes. Assembly quality was assessed following six criteria[23]: (i) 90% of the genome assembly should be included in contigs >500 bp; (ii) 90% of the assembled bases are at >5× read coverage; (iii) contig N50 >5 kb; (iv) scaffold N50 >20 kb; (v) average contig length is >5 kb; (vi) >90% of core genes are present in the assembly. A total of 14 draft microbial genomes passed five or six criteria finally (Supplementary Table 3). We then applied the assembly quality estimation standard published by the Genomic Standards Consortium (GSC) (Supplementary Table 3)[70]. The microbial species designation of 14 assembed genome sequences followed these standards: 1) concordance with taxonomical assignment of CAGs [67]; 2) aligned to the published genome sequences from IMG, NCBI and PATRIC via BLASTN (v2.5.0, default parameters except –e 0.01), with ⩾95% nucleotide identity and ⩾95% genome coverage; 3) assigned by the CheckM(v1.0.12, default parameters) from the Genome Taxonomy DB[71]. Furthermore, gene prediction was executed with Glimmer3.02, while related

annotations of antibiotic resistance and virulence genes were acquired through CARD[72] and VFDB[73]. The SNP mutation associated with macrolide resistance of *M. pneumoniae* was identified by mapping sequencing reads against 23S rRNA genes[74] using BWA.

Correlations between reassembled microbial genomes and disease severity in MPP patients

The correlation between reconstructed microbial genomes with the hospitalization duration and fever peak was assessed. In addition, serum CRP, PCT and eosinophil in 24 hours after hospitalization were also selected to assess the correlation with reassembled microbial genomes via R software. The distributions of relative abundance of 14 reassembled genomes in MPP and healthy children were showed via scatter plot.

Availability of supporting data and materials

The BioProject ID is PRJNA413615. The sequencing data supporting the results of this article are available in the GenBank repository under accession number: SRP119571. The RMGC data set is available in the GigaScience.

Declaration

List of abbreviations

AdV: adenovirus; ARI: acute respiratory infection; BALF: broncho-alveolar lavage fluid; CAGs: co-abundance gene groups; CMV: Cytomegalovirus; EBV: Epstein-Barr virus; GM: gut microbiome; KEGG: Kyoto Encyclopedia of Genes and Genomes; KOs: KEGG orthologous groups; MCL: Markov Cluster Algorithm; NP: nasopharynx; OP: oropharynx; ORFs: open reading frames; PCA: principal component analysis; PCR: Polymerase Chain Reaction; PERMANOVA: Permutational multivariate analysis of variance analysis; PP: pediatric pneumonia; RM: respiratory microbiome; RMGC: respiratory microbial gene catalogue; RSV: respiratory syncytial virus;

Consent for publication

All the guardians of participates consent to publish

Competing Interests

The authors declare no competing financial interests.

Funding

This study was supported by Key Medical Disciplines Building Project of Shenzhen

(SZXJ2017005), Sanming Project of Medicine in Shenzhen (SZSM201512030), and Shenzhen Science and Technology Project (JCYJ20170303155012371 and JCYJ20170816170527583).

Authors' contributions

Y.Z., Y.Y. and K.Z. managed the project. Z.L., G.X. and Y.B. performed the sampling and information collection. W.W. and Q.Y. prepared the DNA extraction. D.L., Q.Z., X.F. and Z.Y. performed the bioinformatics analysis in this work. C.Q., Y.L. and Y.L. optimized the graphs. X.X. and M.L. optimized the data curation. S.L. and Y.Y. guided data interpretation. X.F. developed the website. H.W. and W.D. dealt the data mining and wrote the paper. K.S. and K.Y. polished the article. All authors reviewed this manuscript.

Acknowledge

We thank suggestions from members in Collaborating Group of Pediatric Respiratory Microbiome, Chinese Pediatric Society and Chinese Medical Association. We also thank Mr. Xiaofeng Lin from EasyPub for polishing language when preparing this submission.

Authors' information

Y.Y. is a Russian academician on pediatric and vaccine research. Y.Z is the director of respiratory disease department in Shenzhen Children’s Hospital. S.L is a professor of department of computer science in the City University of Computer Science. K. Z is a professor of Wuhan National Laboratory for Optoelectronics, Huazhong University of Science and Technology.

References

1. Stearns JC, Davidson CJ, McKeon S, Whelan FJ, Fontes ME, Schryvers AB, *et al.* Culture and molecular-based profiles show shifts in bacterial communities of the upper respiratory tract that occur with age. ISME J. 2015; 9**:** 1268.
2. Biesbroek G, Tsivtsivadze E, Sanders EA, Montijn R, Veenhoven RH, Keijser BJ, *et al.* Early respiratory microbiota composition determines bacterial succession patterns and respiratory health in children. Am J Respir Crit Care Med. 2014; 190**:** 1283-92.
3. Biesbroek G, Bosch AA, Wang X, Keijser BJ, Veenhoven RH, Sanders EA, *et*

al. The impact of breastfeeding on nasopharyngeal microbial communities in infants. Am J Respir Crit Care Med. 2014; 190**:** 298-308.

4. Bosch AA, de Steenhuijsen Piters WA, van Houten MA, Chu M, Biesbroek G, Kool J, *et al.* Maturation of the infant respiratory microbiota, environmental drivers and health consequences: a prospective cohort study. Am J Respir Crit Care Med. 2017; 196**:** 1582-90.
5. Charlson ES, Bittinger K, Haas AR, Fitzgerald AS, Frank I, Yadav A, *et al.* Topographical continuity of bacterial populations in the healthy human respiratory tract. Am J Respir Crit Care Med. 2011; 184**:** 957-63.
6. de Steenhuijsen Piters WA, Huijskens EG, Wyllie AL, Biesbroek G, van den Bergh MR, Veenhoven RH, *et al.* Dysbiosis of upper respiratory tract microbiota in elderly pneumonia patients. ISME J. 2016; 10**:** 97-108.
7. Sakwinska O, Bastic Schmid V, Berger B, Bruttin A, Keitel K, Lepage M, *et al.* Nasopharyngeal microbiota in healthy children and pneumonia patients. J Clin Microbiol. 2014; 52**:** 1590-4.
8. Prina E, Ranzani OT, Torres A. Community-acquired pneumonia. Lancet.

2015; 386: 1097-108.

9. Musher DM, Thorner AR. Community-acquired pneumonia. N Engl J Med. 2014; 371: 1619-28.

10. Liu L, Oza S, Hogan D, Perin J, Rudan I, Lawn JE, *et al.* Global, regional, and national causes of child mortality in 2000-13, with projections to inform post-2015 priorities: an updated systematic analysis. Lancet. 2015; 385: 430-40.

11. Dagan R, Bhutta ZA, de Quadros CA, Garau J, Klugman KP, Khuri-Bulos N, *et al.* The remaining challenge of pneumonia: the leading killer of children. Pediatr Infect Dis J. 2011; 30: 1-2.

12. Qin Q, Baoping Xu, Liu X, Shen K. Status of Mycoplasma pneumoniae pneumonia in chinese children: a systematic review. Advances in Microbiology. 2014; 4: 704-11.

13. Lu Z, Dai W, Liu Y, Zhou Q, Wang H, Li D, *et al.* The alteration of nasopharyngeal and oropharyngeal microbiota in children with MPP and non-MPP. Genes (Basel). 2017; 8.

14. Dai W, Wang H, Zhou Q, Feng X, Lu Z, Li D, *et al.* The concordance between upper and lower respiratory microbiota in children with Mycoplasma pneumoniae pneumonia. Emerg Microbes Infect. 2018; 7**:** 92.

15. Hasegawa K, Mansbach JM, Ajami NJ, Espinola JA, Henke DM, Petrosino JF, *et al.* Association of nasopharyngeal microbiota profiles with bronchiolitis severity in infants hospitalised for bronchiolitis. Eur Respir J. 2016; 48**:** 1329-39.

16. de Steenhuijsen Piters WA, Heinonen S, Hasrat R, Bunsow E, Smith B, Suarez-Arrabal MC, *et al.* Nasopharyngeal microbiota, host transcriptome, and disease severity in children with respiratory syncytial virus infection. Am J Respir Crit Care Med. 2016; 194**:** 1104-15.

17. Pettigrew MM, Gent JF, Kong Y, Wade M, Gansebom S, Bramley AM, *et al.* Association of sputum microbiota profiles with severity of community-acquired pneumonia in children. BMC Infect Dis. 2016; 16**:** 317.

18. Vissing NH, Chawes BL, Bisgaard H. Increased risk of pneumonia and bronchiolitis after bacterial colonization of the airways as neonates. Am J

Respir Crit Care Med. 2013; 188**:** 1246-52.

19. Mika M, Mack I, Korten I, Qi W, Aebi S, Frey U, *et al.* Dynamics of the nasal microbiota in infancy: a prospective cohort study. J Allergy Clin Immunol. 2015; 135**:** 905-12.e11.

20. Zhang R, Wang H, Deng J. A 4-Year-Old Girl With Progressive Cough and Abnormal Blood Smear. Clinical Infectious Diseases. 2017; 64**:** 1630–31.

21. Li J, Jia H, Cai X, Zhong H, Feng Q, Sunagawa S, *et al.* An integrated catalog of reference genes in the human gut microbiome. Nat Biotechnol. 2014; 32**:** 834-41.

22. Qin J, Li Y, Cai Z, Li S, Zhu J, Zhang F, *et al.* A metagenome-wide association study of gut microbiota in type 2 diabetes. Nature. 2012; 490**:** 55-60.

23. Zhang C, Yin A, Li H, Wang R, Wu G, Shen J, *et al.* Dietary modulation of gut microbiota contributes to alleviation of both genetic and simple obesity in children. EBioMedicine. 2015; 2**:** 968-84.

24. Rosas-Salazar C, Shilts MH, Tovchigrechko A, Schobel S, Chappell JD, Larkin EK, *et al.* Differences in the nasopharyngeal microbiome during acute

respiratory tract infection with human rhinovirus and respiratory syncytial virus in infancy. J Infect Dis. 2016; 214**:** 1924-28.

25. Stewart CJ, Mansbach JM, Wong MC, Ajami NJ, Petrosino JF, Camargo CAJ, *et al.* Associations of nasopharyngeal metabolome and microbiome with severity among infants with bronchiolitis: a multi-omic analysis. Am J Respir Crit Care Med. 2017; 196**:** 882-91.

26. Quinn RA. Integrating microbiome and metabolome data to understand infectious airway disease. Am J Respir Crit Care Med. 2017; 196**:** 806-07.

27. Yang J, Hooper WC, Phillips DJ, Talkington DF. Cytokines in Mycoplasma pneumoniae infections. Cytokine Growth Factor Rev. 2004; 15**:** 157-68.

28. Peteranderl C, Sznajder JI, Herold S, Lecuona E. Inflammatory responses regulating alveolar ion transport during pulmonary infections. Front Immunol. 2017; 8**:** 446.

29. Miller SI, Ernst RK, Bader MW. LPS, TLR4 and infectious disease diversity. Nat Rev Microbiol. 2005; 3**:** 36-46.

30. Patkee WR, Carr G, Baker EH, Baines DL, Garnett JP. Metformin prevents the

effects of Pseudomonas aeruginosa on airway epithelial tight junctions and restricts hyperglycaemia-induced bacterial growth. J Cell Mol Med. 2016; 20**:** 758-64.

31. Hewitt R, Webber J, Farne H, Trujillo-Torralbo M-B, Footitt J, Molyneaux PL, *et al.* Airway glucose in virus-induced COPD exacerbations. Am J Respir Crit Care Med. 2016; 192**:** A6323.

32. Garnett JP, Nguyen TT, Moffatt JD, Pelham ER, Kalsi KK, Baker EH, *et al.* Proinflammatory mediators disrupt glucose homeostasis in airway surface liquid. J Immunol. 2012; 189**:** 373-80.

33. Kalsi KK, Baker EH, Fraser O, Chung YL, Mace OJ, Tarelli E, *et al.* Glucose homeostasis across human airway epithelial cell monolayers: role of diffusion, transport and metabolism. Pflugers Arch. 2009; 457**:** 1061-70.

34. Philips BJ, Redman J, Brennan A, Wood D, Holliman R, Baines D, *et al.* Glucose in bronchial aspirates increases the risk of respiratory MRSA in intubated patients. Thorax. 2005; 60**:** 761-4.

35. Man WH, de Steenhuijsen Piters WA, Bogaert D. The microbiota of the

respiratory tract: gatekeeper to respiratory health. Nat Rev Microbiol. 2017; 15: 259-70.

36. Ji P, Zhang Y, Wang J, Zhao F. MetaSort untangles metagenome assembly by reducing microbial community complexity. Nat Commun. 2017; 8: 14306.

37. Olm MR, Brown CA-O, Brooks B, Banfield JF. dRep: a tool for fast and accurate genomic comparisons that enables improved genome recovery from metagenomes through de-replication. ISME J. 2017; 11: 2864-68.

38. Saraya T, Kurai D, Nakagaki K, Sasaki Y, Niwa S, Tsukagoshi H, *et al.* Novel aspects on the pathogenesis of Mycoplasma pneumoniae pneumonia and therapeutic implications. Front Microbiol. 2014; 5: 410.

39. Floss HG, Yu TW. Rifamycin-mode of action, resistance, and biosynthesis. Chem Rev. 2005; 105: 621-32.

40. Nesar S, MH. S, Rahim N, Rehman R. Emergence of resistance to fluoroquinolones among gram positive and gram negative clinical isolates. Pak J Pharm Sci. 2012; 25: 877-81.

41. Axelsen PH. A chaotic pore model of polypeptide antibiotic action. Biophys J.

2008; 94**:** 1549-50.

42. Harris M, Clark J, Coote N, Fletcher P, Harnden A, McKean M, *et al.* British Thoracic Society guidelines for the management of community acquired pneumonia in children: update 2011. Thorax. 2011; 66 Suppl 2**:** ii1-23.

43. Bradley JS, Byington CL, Shah SS, Alverson B, Carter ER, Harrison C, *et al.* The management of community-acquired pneumonia in infants and children older than 3 months of age: clinical practice guidelines by the Pediatric Infectious Diseases Society and the Infectious Diseases Society of America. Clin Infect Dis. 2011; 53**:** e25-76.

44. Lee H, Yun KW, Lee HJ, Choi EH. Antimicrobial therapy of macrolide-resistant Mycoplasma pneumoniae pneumonia in children. Expert Rev Anti Infect Ther. 2018; 16**:** 23-34.

45. Hasegawa K, Mansbach JM, Ajami NJ, Espinola JA, Henke DM, Petrosino JF, *et al.* Association of nasopharyngeal microbiota profiles with bronchiolitis severity in infants hospitalised for bronchiolitis. Eur Respir J. 2016; 48**:** 1329-39.

46. Hasegawa K, Linnemann RW, Mansbach JM, Ajami NJ, Espinola JA, Petrosino JF, *et al.* Nasal airway microbiota profile and severe bronchiolitis in infants: a case-control study. Pediatr Infect Dis J. 2017; 36**:** 1044-51.

47. Citti C, Dordet-Frisoni E, Nouvel LX, Kuo CH, Baranowski E. Horizontal gene transfers in Mycoplasmas (Mollicutes). Curr Issues Mol Biol. 2018; 29**:** 3-22.

48. Xiao L, Ptacek T, Osborne JD, Crabb DM, Simmons WL, Lefkowitz EJ, *et al.* Comparative genome analysis of Mycoplasma pneumoniae. BMC Genomics. 2015; 16**:** 610.

49. Rohde H, Qin J, Cui Y, Li D, Loman NJ, Hentschke M, *et al.* Open-source genomic analysis of Shiga-toxin-producing E. coli O104:H4. N Engl J Med. 2011; 365**:** 718-24.

50. Davies MR, Holden MT, Coupland P, Chen JH, Venturini C, Barnett TC, *et al.* Emergence of scarlet fever Streptococcus pyogenes emm12 clones in Hong Kong is associated with toxin acquisition and multidrug resistance. Nat Genet. 2015; 47**:** 84-7.

51. Kutty PK, Jain S, Taylor TH, Bramley AM, Diaz MH, Ampofo K, *et al.* Mycoplasma pneumoniae among children hospitalized with community-acquired pneumonia. Clin Infect Dis. 2019; 68**:** 5-12.

52. Blyth CC, Gerber JS. Macrolides in children with community-acquired pneumonia: panacea or placebo? J Pediatric Infect Dis Soc. 2018; 7**:** 71-77.

53. Yang D, Chen L, Chen ZA-O. The timing of azithromycin treatment is not associated with the clinical prognosis of childhood Mycoplasma pneumoniae pneumonia in high macrolide-resistant prevalence settings. PLoS One. 2018; 13**:** e0191951.

54. Larsen JM, Musavian HS, Butt TM, Ingvorsen C, Thysen AH, Brix S. Chronic obstructive pulmonary disease and asthma-associated Proteobacteria, but not commensal Prevotella spp., promote Toll-like receptor 2-independent lung inflammation and pathology. Immunology. 2015; 144**:** 333-42.

55. Segal LN, Clemente JC, Tsay JC, Koralov SB, Keller BC, Wu BG, *et al.* Enrichment of the lung microbiome with oral taxa is associated with lung inflammation of a Th17 phenotype. Nat Microbiol. 2016; 1**:** 16031.

56. de Dios Caballero J, Vida R, Cobo M, Maiz L, Suarez L, Galeano J, *et al.* Individual patterns of complexity in cystic fibrosis lung microbiota, including predator bacteria, over a 1-year period. MBio. 2017; 8**:** e00959-17.

57. Maier L, Pruteanu M, Kuhn M, Zeller G, Telzerow A, Anderson EE, *et al.* Extensive impact of non-antibiotic drugs on human gut bacteria. Nature. 2018; 555**:** 623-28.

58. Qin J, Li R, Raes J, Arumugam M, Burgdorf KS, Manichanh C, *et al.* A human gut microbial gene catalogue established by metagenomic sequencing. Nature. 2010; 464**:** 59-65.

59. Lloyd-Price J, Mahurkar A, Rahnavard G, Crabtree J, Orvis J, Hall AB, *et al.* Strains, functions and dynamics in the expanded Human Microbiome Project. Nature. 2017; 550**:** 61-66.

60. Yan L, Yang M, Guo H, Yang L, Wu J, Li R, *et al.* Single-cell RNA-Seq profiling of human preimplantation embryos and embryonic stem cells. Nat Struct Mol Biol. 2013; 20**:** 1131-9.

61. Luo R, Liu B, Xie Y, Li Z, Huang W, Yuan J, *et al.* SOAPdenovo2: an

empirically improved memory-efficient short-read de novo assembler. Gigascience. 2012; 1: 18.

62. Zhu W, Lomsadze A, Borodovsky M. Ab initio gene identification in metagenomic sequences. Nucleic Acids Res. 2010; 38: e132.

63. Delcher AL, Bratke KA, Powers EC, Salzberg SL. Identifying bacterial genes and endosymbiont DNA with Glimmer. Bioinformatics. 2007; 23: 673-9.

64. Li W, Godzik A. Cd-hit: a fast program for clustering and comparing large sets of protein or nucleotide sequences. Bioinformatics. 2006; 22: 1658-9.

65. Enright AJ, Van Dongen S, Ouzounis CA. An efficient algorithm for large-scale detection of protein families. Nucleic Acids Res. 2002; 30: 1575-84.

66. Chao A. Estimating the population size for capture-recapture data with unequal catchability. Biometrics. 1987; 43: 783-91.

67. Nielsen HB, Almeida M, Juncker AS, Rasmussen S, Li J, Sunagawa S, *et al.* Identification and assembly of genomes and genetic elements in complex metagenomic samples without using reference genomes. Nat Biotechnol. 2014;

32**:** 822-8.

68. Shannon P, Markiel A, Ozier O, Baliga NS, Wang JT, Ramage D, *et al.* Cytoscape: a software environment for integrated models of biomolecular interaction networks. Genome Res. 2003; 13**:** 2498-504.

69. Zerbino DR, Birney E. Velvet: algorithms for de novo short read assembly using de Bruijn graphs. Genome Res. 2008; 18**:** 821-9.

70. Bowers RM, Kyrpides NC, Stepanauskas R. Minimum information about a single amplified genome (MISAG) and a metagenome-assembled genome (MIMAG) of bacteria and archaea. 2017; 35**:** 725-31.

71. Parks DH, Chuvochina M, Waite DW, Rinke C, Skarshewski A, Chaumeil PA, *et al.* A standardized bacterial taxonomy based on genome phylogeny substantially revises the tree of life. Nat Biotechnol. 2018; 36**:** 996-1004.

72. Jia B, Raphenya AR, Alcock B, Waglechner N, Guo P, Tsang KK, *et al.* CARD 2017: expansion and model-centric curation of the comprehensive antibiotic resistance database. Nucleic Acids Res. 2017; 45**:** D566-D73.

73. Chen L, Zheng D, Liu B, Yang J, Jin Q. VFDB 2016: hierarchical and refined

dataset for big data analysis--10 years on. Nucleic Acids Res. 2016; 44**:** D694-7.

74. Ji M, Lee NS, Oh JM, Jo JY, Choi EH, Yoo SJ, *et al.* Single-nucleotide polymorphism PCR for the detection of Mycoplasma pneumoniae and determination of macrolide resistance in respiratory samples. J Microbiol Methods. 2014; 102**:** 32-6.

Tables

Table 1. Sample information

	**Pneumonia Patients (n=76)**	**Healthy Children (n=171)**
**Characteristics**		
Gender		
Female	24	87
Male	52	84
Age (years)	2.9(0.2-12.7)	4.3(0.1-8.9)
Sampling Site		
OP	75	171
NP	42	-
Lung	46	-
Delivery Mode		
Vaginally born	46	102
Cesarean section	30	69
Feeding Pattern		
Breast	48	84

Breast+Milk	12	66
Milk feed	16	21
Family history of allergy	1	-
History of pneumonia	14	-
Asthma	-	-
**Clinical symptoms**		
Lung consolidation, atelectasis, infiltration	76	NA
Fever	44	-
Cough	72	-
Wheezing	20	-
Hospitalization time (days)	9(2-37)	-
CRP(<0.499mg/l)	22	NA
PCT(<0.5ng/ml)	73	NA
Eosinophils(0.5–5%)	44	NA

"-" represents no detection result; "NA" represents not available; CRP, C-response protein; PCT, procalcitonin

Figure Legends

Figure 1. **Construction of the human RMGC**. Genome assembly was performed for each sample with ⩾650 Mbp of data. For samples with <650 Mbp of data, the data from the same respiratory site (NP, OP or the lung) were mixed and assembled. Gene predictions were conducted for all assembled contigs with ⩾500 bp and respiratory bacterial genomes in IMG. Genes with ⩾100 bp were retained. Respiratory gene sets in HMP and PARTIC were combined to construct the non-redundant RMGC containing 2,245,343 genes.

Figure 2. Rarefaction curves for genes and KOs/gene families. a, Rarefaction curve for the gene count. **b,** Rarefaction curve for Chao2. The RMGC captured 90.52% of the prevalent genes. **c,** Rarefaction curve for KOs/gene families. Known functions saturate quickly to 6,346 groups. After including novel gene families, the rarefaction curve plateaus when 12,924 groups are detected. Boxes represent the interquartile ranges (IQRs) between the first and third quartiles, and the line inside the box represents the median value. Whiskers represent the lowest or highest values within values 1.5 times the IQR from the first or third quartiles. Circles represent data points located outside of the whiskers.

Figure 3. Core microbial species in healthy OP microbiota. The barplot on the top represent the prevalence of each core species, boxplot beneath the barplot means the relative abundance of each core species. The specific color stands for different phylum.

Figure 4. Differentiation of OP microbial samples between healthy children and MPP patients. a, Gene counts in the OP microbiomes of healthy children and children with pneumonia. **b,** Alpha diversity of the OP microbiome in healthy children

and children with pneumonia. Boxes represent the IQRs between the first and third quartiles, and the line inside the box represents the median. Whiskers represent the lowest or highest values within values 1.5 times the IQR from the first or third quartiles. Points represent data located outside of the whiskers. *** represents *p*-value ⩽0.001.

Figure 5. Phylogenetic and functional alterations in children with pneumonia. a, Size of the circle represents the average relative abundance of CAGs in healthy children or children with pneumonia. A line between two circles indicates a Spearman's rank correlation coefficient ⩾0.6 and an adjusted *p*-value ⩽0.05. The phylum and genus corresponding to each CAG are indicated by the information listed on the left. **b,** The X-axis represents level-2 functional categories in KEGG, and the colour of the characters represents level-1 functional categories, which are listed on the right. The Y-axis shows the relative abundance of level-2 functional categories. *,** and *** represent adjusted *p*-value ⩽0.05, ⩽0.01 and ⩽0.001, respectively.

Figure 6. Virulence-factor genes (VFGs) and antibiotic-resistance genes (ARGs) on *Mycoplasma pneumoniae* genome. The tracks from outside to inside represent

ARGs, genes on plus strand, genes on negative strand and GC skew, respectively. VFGs painted with different colours refer to the different types of VFGs.

Figure 7. Comparison of relative abundance of 14 re-assembled genomes between healthy children and MPP patients. The blue circles and red triangles represent the microbial relative abundance of healthy children and MPP patients. Solid dot and paired whiskers represent the mean and SD of each microbial relative abundance. *, ** and *** represents *p*-value $\leqslant 0.05$, $\leqslant 0.01$ and $\leqslant 0.001$, respectively. NS stands for no statistical significance.

Figure 1

Click here to access/download;Figure;Figure 1.pdf

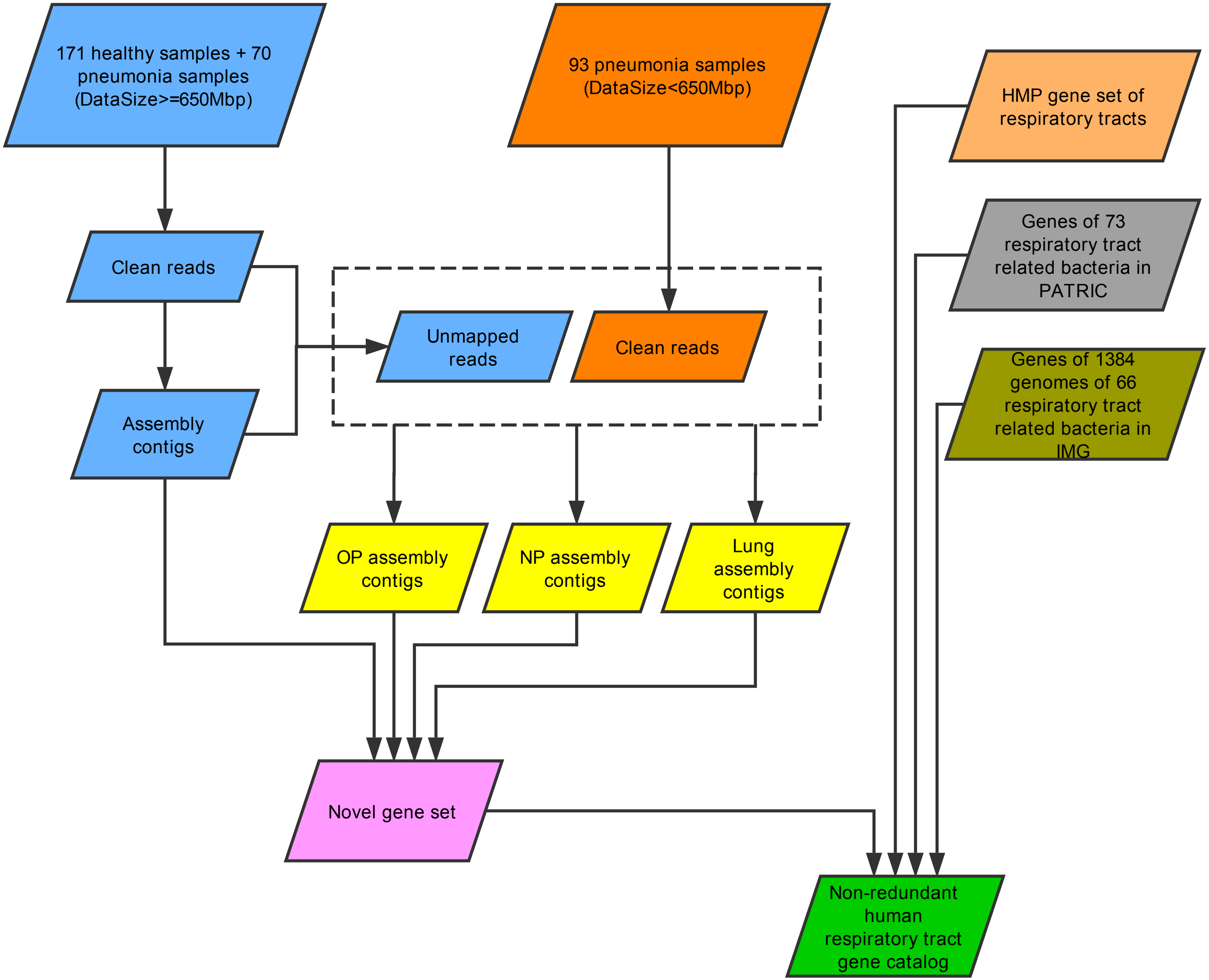

Figure 2

Click here to access/download;Figure;Figure 2.pdf

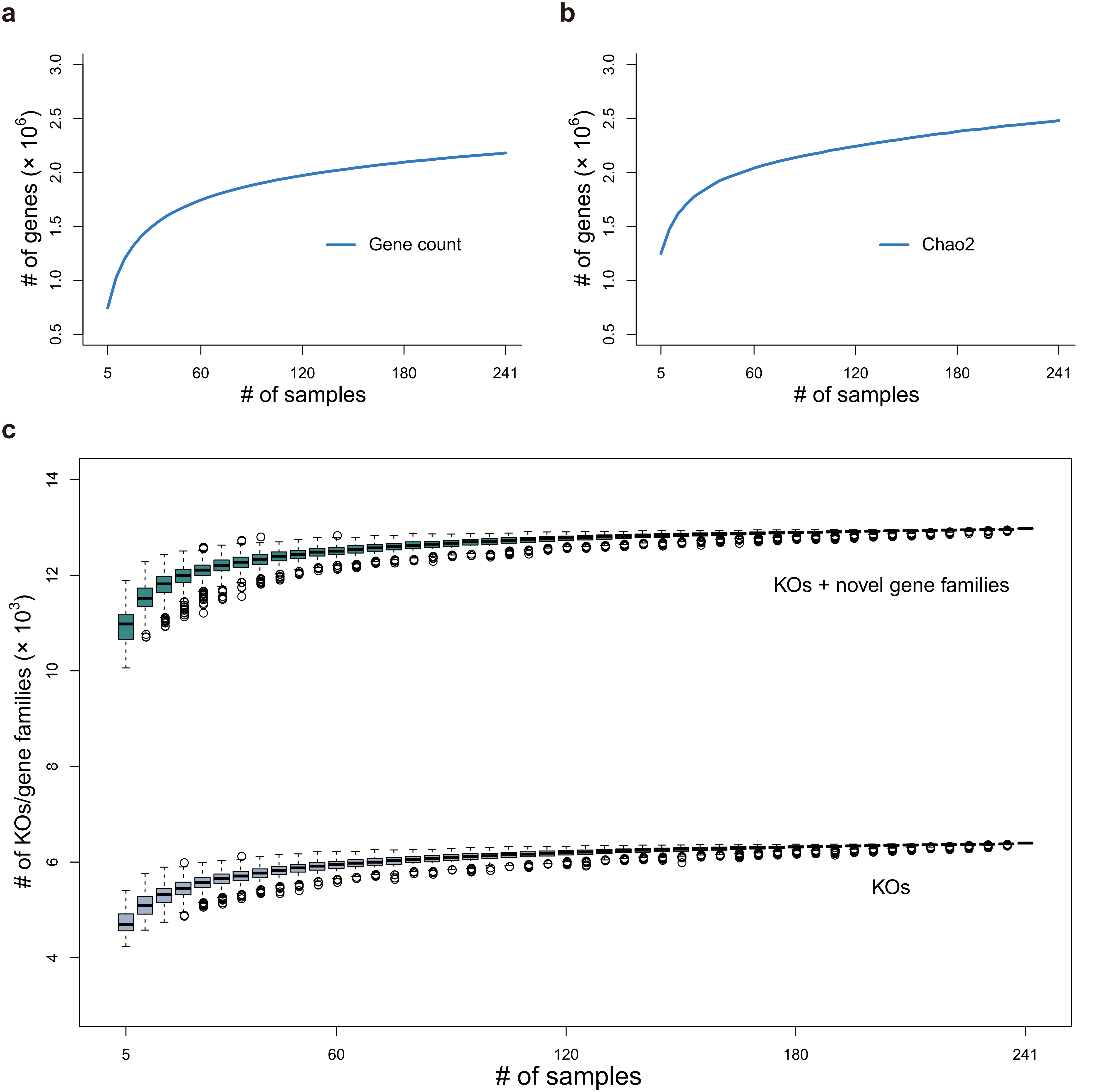

Figure 3

Click here to access/download;Figure;Figure 3.pdf

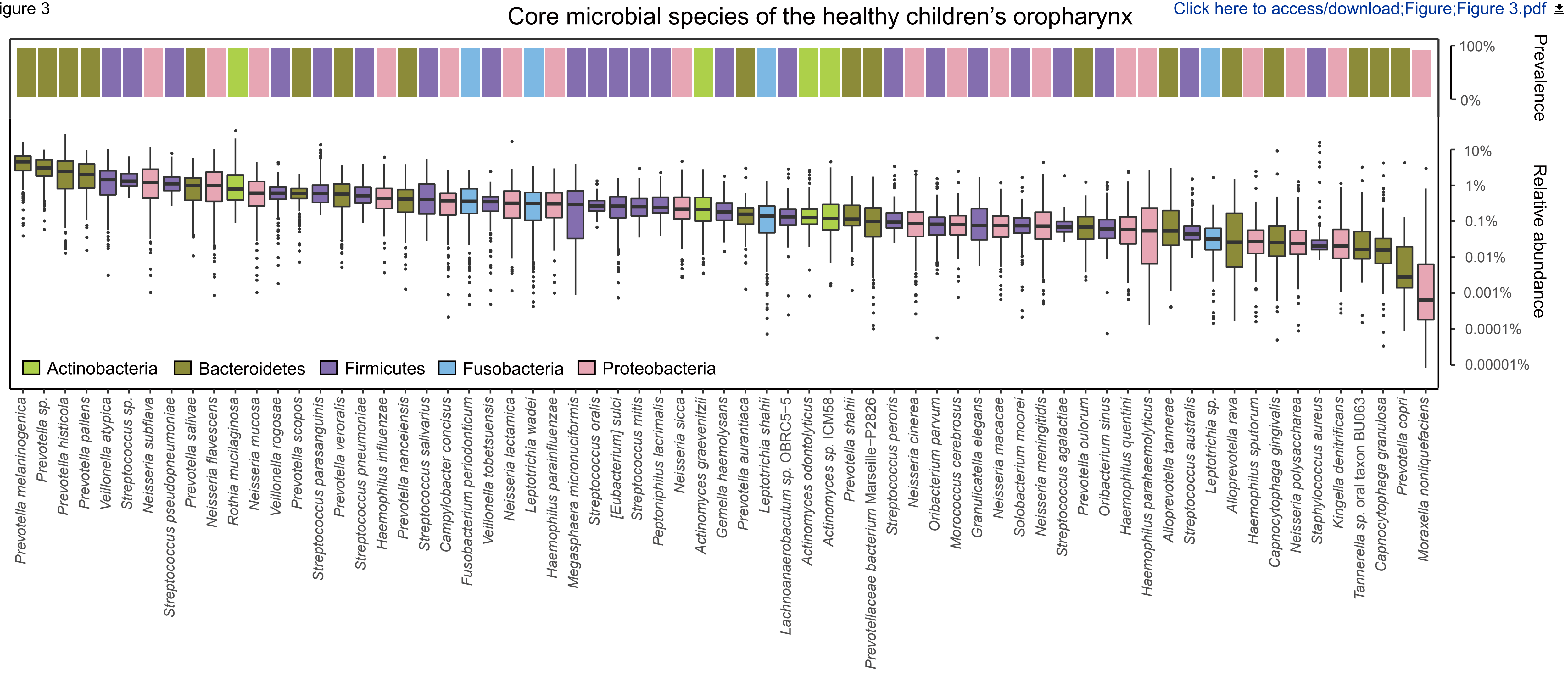

Figure 4
Click here to access/download;Figure;Figure 4.pdf

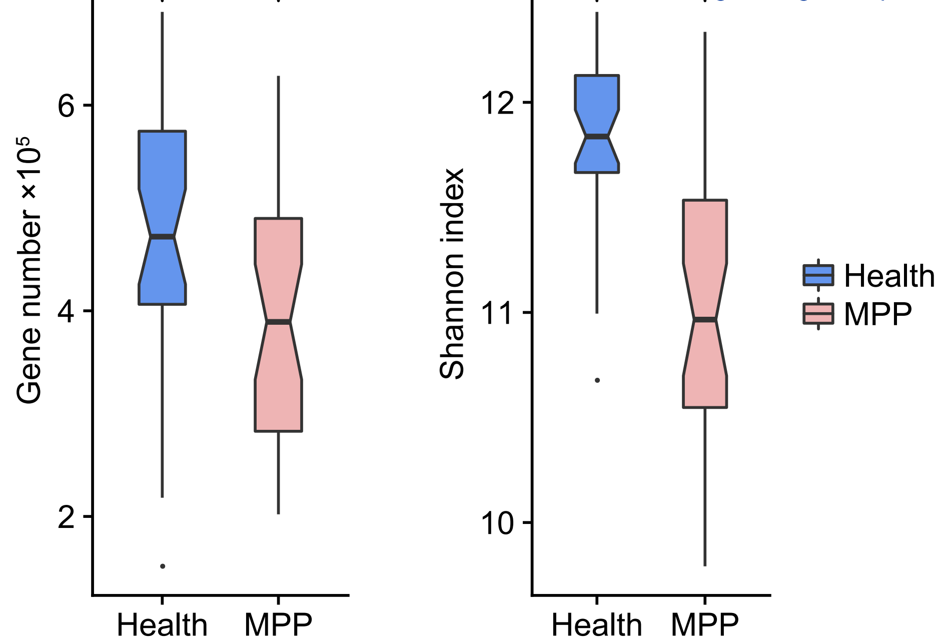

Figure 5

Click here to access/download;Figure;Figure 5.pdf

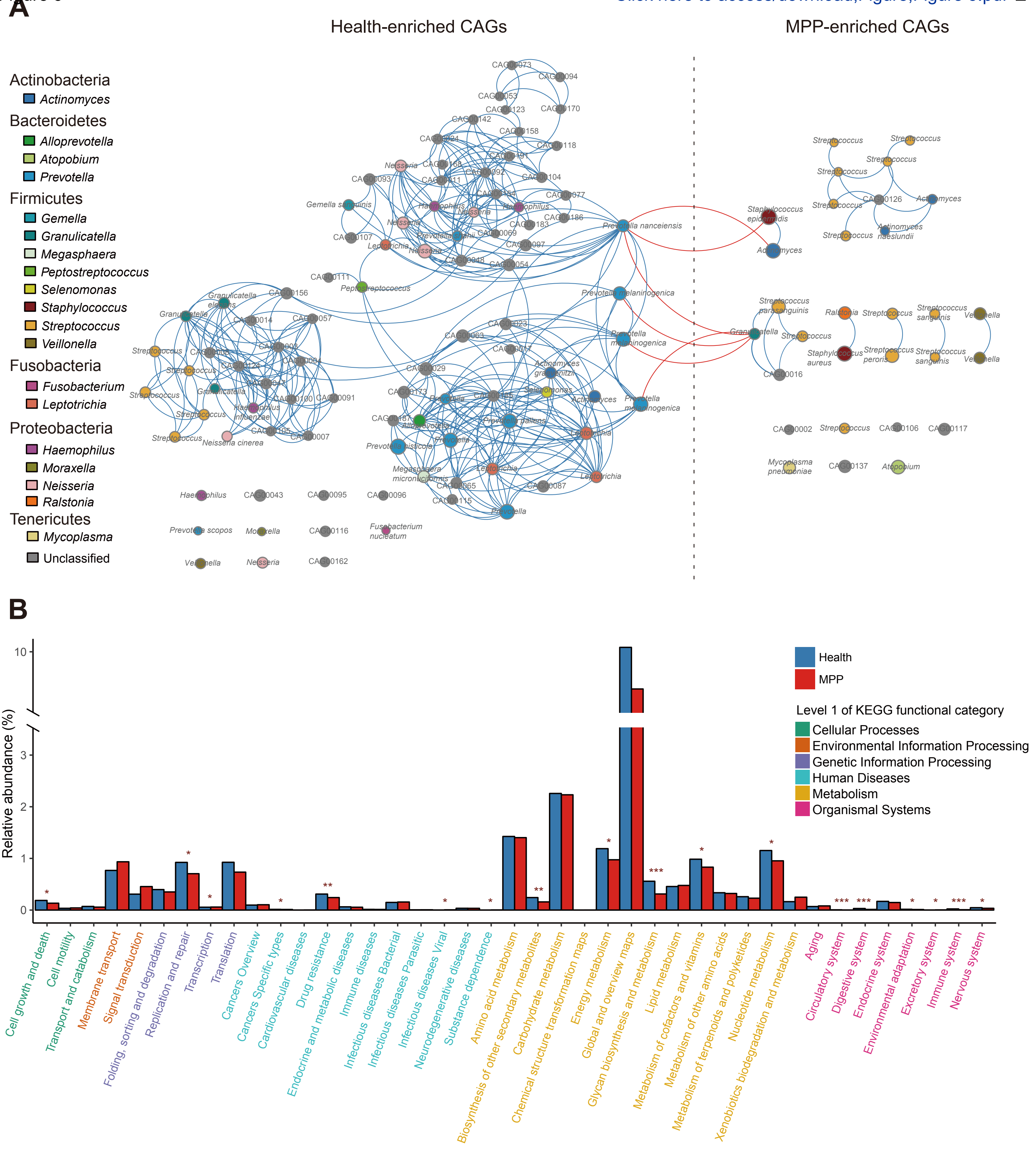

Click here to access/download;Figure;Figure 6.pdf

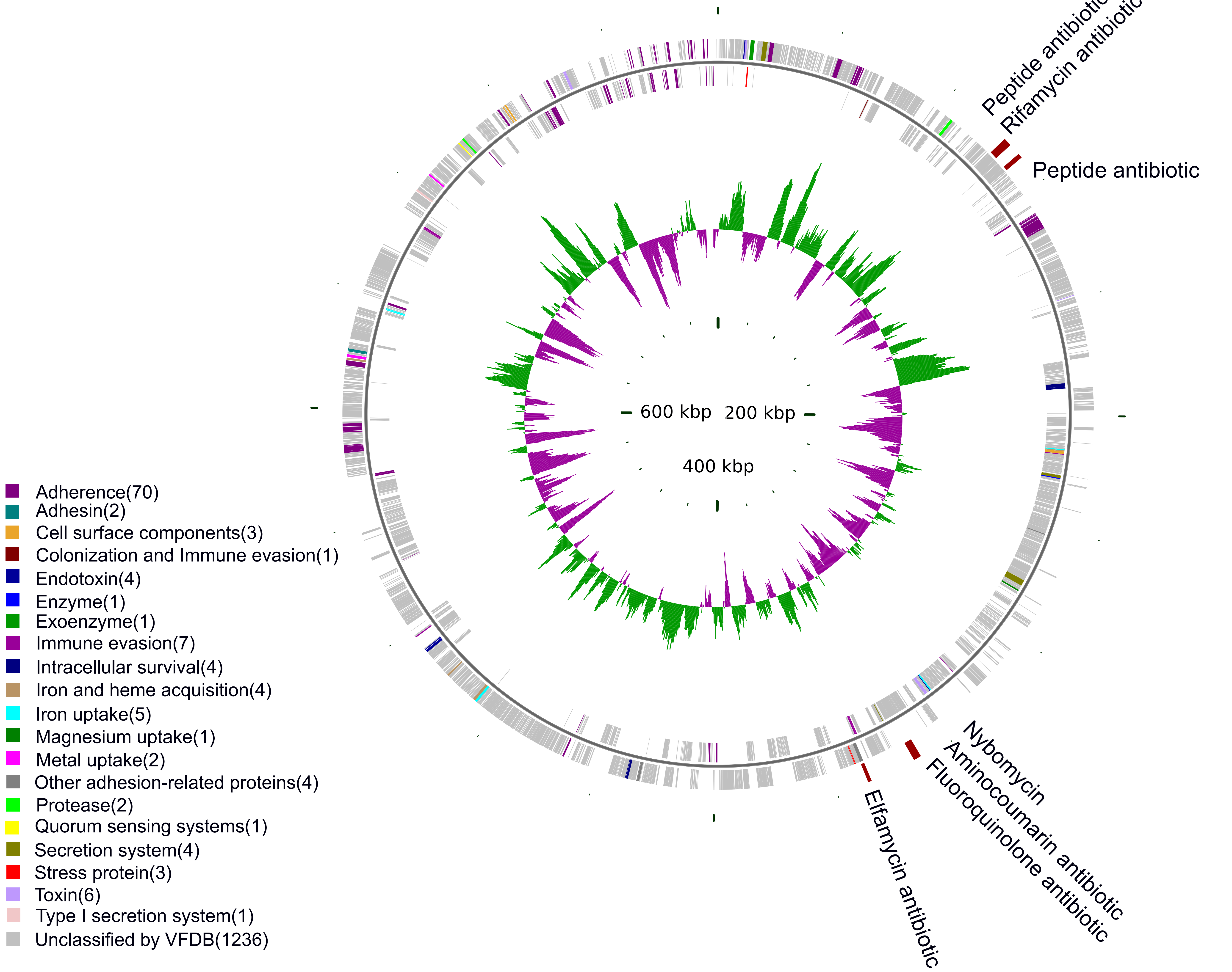

Figure 7

Click here to access/download;Figure;Figure 7.pdf

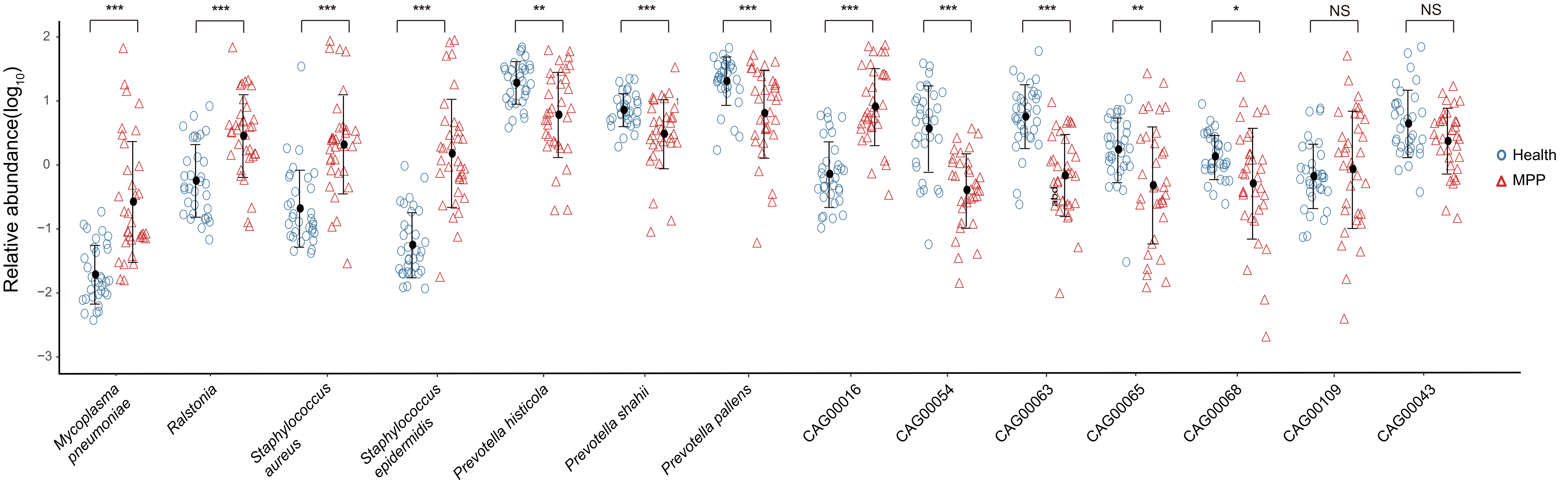

Supplemental Material Legends

Click here to access/download
Supplementary Material
Supplemental material legends.docx

Supplementary Figure 1

Click here to access/download
Supplementary Material
Supplementary Figure 1.pdf

Supplementary Figure 2

Click here to access/download
Supplementary Material
Supplementary Figure 2.pdf

Supplementary Figure 3

Click here to access/download
Supplementary Material
Supplementary Figure 3.pdf

Supplementary Table 1

Click here to access/download
Supplementary Material
Supplementary Table 1.xlsx

Supplementary Table 2

Click here to access/download
Supplementary Material
Supplementary Table 2.xlsx

Supplementary Table 3

Click here to access/download
Supplementary Material
Supplementary Table 3.xlsx

Supplementary Table 4

Click here to access/download
Supplementary Material
Supplementary Table 4.xlsx

Supplementary Table 5

Click here to access/download
Supplementary Material
Supplementary Table 5.xlsx

Supplementary Table 6

Click here to access/download
Supplementary Material
Supplementary Table 6.xlsx
