# Supplementary material for: An integrated respiratory microbial gene catalogue to better understand the microbial aetiology of Mycoplasma pneumoniae pneumonia
Source: Gigascience. 2019 Jul 31;8(8):giz093. doi: 10.1093/gigascience/giz093 (PMC6669060; doi:10.1093/gigascience/giz093)
Supplement: giz093_GIGA-D-19-00029_Revision_3 [file giz093_giga-d-19-00029_revision_3.pdf]

GigaScience

An integrated respiratory microbial gene catalogue to better understand the microbial aetiology of Mycoplasma pneumoniae pneumonia

--Manuscript Draft--

**Manuscript Number:**	GIGA-D-19-00029R3
**Full Title:**	An integrated respiratory microbial gene catalogue to better understand the microbial aetiology of Mycoplasma pneumoniae pneumonia
**Article Type:**	Research
**Funding Information:**	Sanming Project of Medicine in Shenzhen (SZSM201512030) — Dr Yuejie Zheng<br>Shenzhen Science and Technology Project (JCYJ20170303155012371) — Mr Heping Wang<br>Shenzhen Science and Technology Project (JCYJ20170816170527583) — Mr Heping Wang<br>Key Medical Disciplines Building Project of Shenzhen (SZXJ2017005) — Mr Zhiwei Lu<br>Guangdong Medical Research Foundation (A2017213) — Mrs Yanmin Bao
**Abstract:**	Background<br>The imbalanced respiratory microbiota observed in pneumonia causes high morbidity and mortality in childhood. Respiratory metagenomic analysis demands a comprehensive microbial gene catalogue, which will significantly advance our understanding of host–microorganism interactions.<br><br>Results<br>We collected 334 respiratory microbial samples from 171 healthy children and 76 children with pneumonia. The respiratory microbial gene catalogue we established comprised 2.25 million non-redundant microbial genes, covering 90.52% of prevalent genes. The major oropharyngeal microbial species found in healthy children were Prevotella and Streptococcus. In children with Mycoplasma pneumoniae pneumonia (MPP), oropharyngeal microbial diversity and associated gene numbers decreased compared to those of healthy children. The concurrence network of oropharyngeal microorganisms in patients predominantly featured Staphylococcus spp. and M. pneumoniae. Functional orthologues, which are associated with the metabolism of various lipids, membrane transport and signal transduction, accumulated in the oropharyngeal microbiome of children with pneumonia. Several antibiotic resistance genes and virulence factor genes were identified in the genomes of M. pneumoniae and 13 other microorganisms reconstructed via metagenomic data. Although the common macrolide/beta-lactam resistance genes were not identified in the assembled M. pneumoniae genome, a single nucleotide polymorphism (A2063G) related to macrolide resistance was identified in a 23S rRNA gene.<br><br>Conclusions<br>The results of this study will facilitate exploration of unknown microbial components and host–microorganism interactions in studies of the respiratory microbiome. It will also yield further insights into the microbial aetiology of MPP.
**Corresponding Author:**	Yuejie Zheng<br>Shenzhen Children's Hospital<br>Shenzhen, Guangdong CHINA
**Corresponding Author Secondary Information:**	
**Corresponding Author's Institution:**	Shenzhen Children's Hospital
**Corresponding Author's Secondary Institution:**	

Powered by Editorial Manager® and ProduXion Manager® from Aries Systems Corporation

**First Author:**	Wenkui Dai
**First Author Secondary Information:**	
**Order of Authors:**	Wenkui Dai
	Heping Wang
	Dongfang Li
	Qian Zhou
	Xin Feng
	Zhenyu Yang
	Wenjian Wang
	Chuangzhao Qiu
	Zhiwei Lu
	Ximing Xu
	Mengxuan Lyu
	Gan Xie
	Yinhu Li
	Yanmin Bao
	Yanhong Liu
	Kunling Shen
	Kaihu Yao
	Xikang Feng
	Yonghong Yang
	Shuaicheng Li
	Ke Zhou
	Yuejie Zheng
**Order of Authors Secondary Information:**	
**Response to Reviewers:**	Dear Editors,<br><br>Thanks for your professional assessments and comments.<br>We have addressed the comments and approved the revision.<br>Please kindly check the updated manuscript without tracked changes.<br><br>Yuejie Zheng
**Additional Information:**	
**Question**	**Response**
Are you submitting this manuscript to a special series or article collection?	No
**Experimental design and statistics**<br><br>Full details of the experimental design and statistical methods used should be given in the Methods section, as detailed in our Minimum Standards Reporting Checklist. Information essential to interpreting the	Yes

data presented should be made available in the figure legends.<br><br>Have you included all the information requested in your manuscript?	
**Resources**<br><br>A description of all resources used, including antibodies, cell lines, animals and software tools, with enough information to allow them to be uniquely identified, should be included in the Methods section. Authors are strongly encouraged to cite Research Resource Identifiers (RRIDs) for antibodies, model organisms and tools, where possible.<br><br>Have you included the information requested as detailed in our Minimum Standards Reporting Checklist?	Yes
**Availability of data and materials**<br><br>All datasets and code on which the conclusions of the paper rely must be either included in your submission or deposited in publicly available repositories (where available and ethically appropriate), referencing such data using a unique identifier in the references and in the "Availability of Data and Materials" section of your manuscript.<br><br>Have you have met the above requirement as detailed in our Minimum Standards Reporting Checklist?	Yes

Manuscript
Click here to view linked References
Click here to access/download;Manuscript;Manuscript-revision-20190709.docx

An integrated respiratory microbial gene catalogue to better understand the microbial aetiology of Mycoplasma pneumoniae pneumonia

Wenkui Dai[2,#], Heping Wang[1,#], Dongfang Li[3,#], Qian Zhou[4], Xin Feng[4], Zhenyu Yang[4], Wenjian Wang[1], Chuangzhao Qiu[4], Zhiwei Lu[1], Ximing Xu[5], Mengxuan Lyu[2], Gan Xie[1], Yinhu Li[4], Yanmin Bao[1], Yanhong Liu[4], Kunling Shen[1,6], Kaihu Yao[1,6], Xikang Feng[2], Yonghong Yang[1,4,6], Shuaicheng Li[2], Ke Zhou[3,*], Yuejie Zheng[1,*]

[1]Department of Respiratory Diseases, Shenzhen Children's Hospital, Shenzhen 518026, China

[2]Department of Computer Science, City University of Hong Kong, Hong Kong 999077, China

[3]Wuhan National Laboratory for Optoelectronics, Huazhong University of Science and Technology, No. 1037 Luoyu Road, Wuhan 430074, China

[4]Department of Microbial Research, WeHealthGene Institute, Shenzhen 518000, China

[5]Institute of Statistics, Nankai University, No. 94 Weijin Road, Tianjin 300071, China

[6]Department of Respiratory Diseases, Beijing Children's Hospital, Beijing 100045, China

[#]These authors contributed equally to this work.

[*]Corresponding authors

ORCIDs and email addresses:

Wenkui Dai, 0000-0002-9723-1416, daiwenkui84@gmail.com; Heping Wang, 0000-0002-9527-1334, szetgmy@163.com; Dongfang Li, 0000-0002-6587-2892, loveli_biocc@163.com; Qian Zhou, 0000-0002-8433-2875, zhouqian@wehealthgene.com; Xin Feng, 0000-0003-1853-7255, fengxin@wehealthgene.com; Zhenyu Yang, yangzhy@wehealthgene.com; Wenjian Wang, 0000-0001-9459-3764, dhbk2005@163.com; Chuangzhao Qiu, 0000-0001-5560-0992, qiuchzh@wehealthgene.com; Zhiwei Lu, luzhiwei1950@163.com; Ximing Xu, ximing@nankai.edu.cn; Mengxuan Lyu, mengxualv2-c@my.cityu.edu.hk; Gan Xie, 0000-0001-6041-2523,

xiegan1987@163.com; Yinhu Li, 0000-0001-6378-6571, liyh@wehealthgene.com; Yanmin Bao, baoyanming1978@163.com; Yanhong Liu, liuyanhong@wehealthgene.com; Kunling Shen, kunlingshen1717@163.com; Kaihu Yao, jiuhu2655@sina.com; Xikang Feng, xikangfeng2-c@my.cityu.edu.hk; Yonghong Yang, 0000-0002-8423-1652, yyh628628@sina.com; Shuaicheng Li, 0000-0001-6246-6349; shuaicli@cityu.edu.hk; Ke Zhou, k.zhou@hust.edu.cn; Yuejie Zheng, 0000-0003-1094-2395, shine1990@sina.com.

Abstract

Background: The imbalanced respiratory microbiota observed in pneumonia causes high morbidity and mortality in childhood. Respiratory metagenomic analysis demands a comprehensive microbial gene catalogue, which will significantly advance our understanding of host–microorganism interactions. **Results:** We collected 334 respiratory microbial samples from 171 healthy children and 76 children with pneumonia. The respiratory microbial gene catalogue we established comprised 2.25 million non-redundant microbial genes, covering 90.52% of prevalent genes. The

major oropharyngeal microbial species found in healthy children were *Prevotella* and *Streptococcus*. In children with *Mycoplasma pneumoniae* pneumonia (MPP), oropharyngeal microbial diversity and associated gene numbers decreased compared to those of healthy children. The concurrence network of oropharyngeal microorganisms in patients predominantly featured *Staphylococcus spp.* and *M. pneumoniae*. Functional orthologues, which are associated with the metabolism of various lipids, membrane transport and signal transduction, accumulated in the oropharyngeal microbiome of children with pneumonia. Several antibiotic resistance genes and virulence factor genes were identified in the genomes of *M. pneumoniae* and 13 other microorganisms reconstructed via metagenomic data. Although the common macrolide/beta-lactam resistance genes were not identified in the assembled *M. pneumoniae* genome, a single nucleotide polymorphism (A2063G) related to macrolide resistance was identified in a 23S rRNA gene. **Conclusions:** The results of this study will facilitate exploration of unknown microbial components and host–microorganism interactions in studies of the respiratory microbiome. It will also yield further insights into the microbial aetiology of MPP.

Keywords: Pneumonia; *Mycoplasma pneumoniae*; Oropharynx; Microbiome; Respiratory microbial gene catalogue

Background

Several microorganisms have been identified as indispensible for the respiratory microbiota (RM) [1–5]. In cases of pneumonia, an imbalance in the levels of the microorganisms within the RM [6, 7] is a leading cause of high morbidity and mortality [8, 9] worldwide, especially in children under 5 years old [10, 11]. In recent years, the number of cases of *Mycoplasma pneumoniae* pneumonia (MPP) in Chinese children has been increasing [12] and the microbial aetiology of this disease remains poorly understood.

Previous studies by our group have revealed altered RM in children with MPP [13, 14]. However, current RM research has mainly focused on analysis of 16S rRNA [6, 7, 15, 16], which only provides cues about known bacterial components at the genus level. Emerging 16S rRNA analysis has revealed an imbalanced microbial structure in the respiratory tracts of children with pneumonia [7, 17, 18]; however,

changes in microbial functions and the species-level microbial components of the RM of patients with MPP remain unexplored. In addition, current multi-omics studies are limited to explorations of known bacterial genomes in the RM [15]. Nevertheless, the RM includes many unknown microbial species [1–3, 5, 6] that require further exploration.

A comprehensive catalogue of reference genes is crucial for in-depth functional metagenomic analysis such as species/gene profiling, discovery of microbial biomarkers and functional annotation. Given that the RM varies with the environment [19], age [1, 2, 4] and disease [6, 7, 15, 16], we took samples from the nasopharynx (NP), oropharynx (OP) and lungs of 76 children with pneumonia and OP samples from 171 healthy children in China. These samples were used to establish an integrated respiratory microbial gene catalogue (RMGC), with which to study the imbalanced RM in Chinese children with MPP. Using this catalogue, we assessed the microbial components and functions in the OP microbiota of healthy children and children with MPP, as well as the characteristics of recovered microbial genomes.

Data Description

Between 3 July 2016 and 27 August 2016, 247 patients were recruited from the hospitalization zone in the Department of Respiratory Diseases of Shenzhen Children's Hospital, China. Inclusion criteria were characteristic chest radiographic abnormalities consistent with pneumonia, exclusion of asthma and clearance of respiratory infections or exposure to antibiotics within one month of sampling (Table 1). NP (25-800-A-50, Puritan, Guilford, ME, USA) and OP (155C, COPAN, Murrieta, CA, USA) swabs were collected from 76 hospitalized patients within 24 hours of hospitalization and before the administration of antibiotics. Broncho-alveolar lavage fluid (BALF) was collected 2–15 days after hospitalization (Supplementary Table 1).

Healthy children were recruited during physical examinations at Shenzhen Children's Hospital, China, between July and August of 2016. OP swabs were collected from 171 healthy children meeting the following inclusion criteria: no diagnosis of asthma or family history of allergy; no history of pneumonia; a lack of wheezing, fever, cough or other respiratory/allergic symptoms at sampling 1 month prior to the study and 1 week after sampling; no exposure to antibiotics 1 month prior to sampling.

All samples were collected by an experienced clinician. Samples were stored at −80°C within 20 minutes of collection and DNA was extracted within 10 days of sampling. A TGuide S32 Magnetic Swab Genomic DNA Kit (DP603-T2, TIANGEN Biotech (Beijing) Co., Ltd., Beijing, China) was utilized to extract the DNA. Metagenomic sequencing was performed using the Illumina Hi-Seq platform (San Diego, USA) according to the manufacturer’s instructions. Unused swabs and DNA extraction kits from the same batch served as negative controls to assess DNA contamination.

Sample information and data output

Two hundred forty-seven children aged <13 years were enrolled in this study (Table 1 and Supplementary Table 1). After removing host contamination and low-quality data, metagenomic sequencing produced 4,765,288,986 reads, with an average of 14,267,332 reads per sample. The DNA concentration of unused sampling swabs and DNA extraction kits was less than 0.01 ng/μl, whereas the DNA concentration was greater than 80 ng/μl in sampling swabs and BALF. Furthermore, the DNA

amplification results of extracted bacterial DNA were less than 0.01 nmol/l for the enveloped sampling or extraction materials (Supplementary Figure 1)

Construction of the RMGC

By applying metagenomics sequencing data from 247 children and three respiratory-related microbial genome/gene resources (Figure 1), we constructed a comprehensive RMGC with 2,245,343 non-redundant ORF. Data are freely accessible on our website [20]. The total length of ORFs in the RMGC was 1.71 Gbp and the average length was 760 nt (range: 102–32,241 nt). We selected 241 samples with ≥650 Mb data to examine the coverage of the microbial genes in the RMGC. In accordance with the rarefaction curve, 90.52% of prevalent microbial genes were captured in the RMGC (Figure 2a and 2b).

Taxonomic assessment and functional annotation of the RMGC

Based on taxonomic profiling, 1,281,673 genes (57.08% of RMGC) were assigned to phyla and 1,143,382 genes (50.92% of RMGC) were assigned to genera, representing

56.58% and 51.75% of the sequencing reads, respectively. A total of 617,968 genes (25.92% of RMGC) were annotated to known bacterial species, representing 33.49% of the sequencing reads. The RMGC was dominated by the phyla Firmicutes, Bacteroides, Proteobacteria, Actinobacteria and Fusobacteria, while the most prevalent microbial genera were *Staphylococcus*, *Streptococcus*, *Haemophilus*, *Corynebacterium*, *Dolosigranulum*, *Prevotella*, *Blautia*, *Rothia*, *Porphyromonas*, *Lactobacillus*, *Veillonella*, *Fusobacterium* and *Leptotrichia*. Unknown microbial species accounted for 9.62–55.50% of the RMGC. Detailed taxonomic information of the RMGC is deposited on our website [20].

Metagenomic analysis revealed a genus-level microbial structure resembling the results of the 16S rRNA analysis (Supplementary Figure 2). Notably, a greater proportion of microbial genera remained unclassified in the metagenomic analysis than in the 16S rRNA analysis; this might be attributed to the wide detection by metagenomics sequencing and limited reference microbial genomes.

By aligning the RMGC to the KEGG database, 6,408 KEGG orthologous (KO) groups were identified, including 853,446 genes representing 37.85% of the total

sequencing data. As more samples were included, known microbial functions (annotated by KEGG) saturated quickly to 6,346 groups (Figure 2c). Combining novel gene families, the rarefaction curve plateaued when 12,924 groups were detected (Figure 2c). Upon alignment to the eggNOG database, 53.95% of genes in the RMGC were assigned to known functional categories.

Core microbial species in the OP microbiota of healthy children

We acquired 67 core species across five dominant phyla: Bacteroidetes, Firmicutes, Proteobacteria, Actinobacteria and Fusobacteria (Figure 3). *Prevotella melaninogenica* (4.38 ± 2.91%, mean ± sd), *Prevotella sp.* (3.06 ± 1.92%), *P. histicola* (3.23 ± 3.58%), *P. pallens* (2.31 ± 1.88%) and *Veillonella atypical* (1.60 ± 1.44%) were the top five microbial species. In addition, *Streptococcus pseudopneumoniae* (1.26 ± 0.96%), *Haemophilus influenzae* (0.60 ± 0.68%), *S. pneumoniae* (0.60 ± 0.50%), *H. parainfluenzae* (0.42 ± 0.49%) and *Staphylococcus aureus* (0.27 ± 1.52%), which were generally defined as opportunistic pathogens, were also prevalent in the OP microbiota of healthy children (Figure 3).

Microbial structure and functions of the OP microbiome of children with MPP differed from those of healthy children

Based on permutational analysis of variance (PERMANOVA), onset of pneumonia is the most significant factor (adjusted *P*-value <0.001) explaining the variations observed in OP microbial samples. This is followed by feeding pattern (adjusted *P*-value = 0.037) and age (adjusted *P*-value = 0.048). Compared with healthy children, children with MPP exhibited significantly decreased microbial gene numbers and alpha diversity of the OP microbiota (Figure 4a and 4b). Thirty co-abundance gene groups (CAGs) accumulated significantly in the OP microbiota of children with MPP, comprising six unknown and 24 known microbial species. These species were primary respiratory pathogens such as *M. pneumoniae*, *Staphylococcus epidermidis* and *S. aureus* (Figure 5a). Ninety-five CAGs were enriched in the OP microbiota of healthy children, including prevalent colonizers such as *Prevotella* species (Figure 5a). The microbial co-occurrence networks of children with MPP were simpler than those of healthy children; negative correlations were only identified between

healthy-enriched and MPP-enriched CAGs (Figure 5a). For example, healthy-enriched *Prevotella spp.* negatively correlated with MPP-enriched *S. epidermidis* ($r < -0.60$, adjusted P-value ≤ 0.05, Figure 5a).

By comparing functional KEGG annotations (Supplementary Table 2), we assessed the functional alterations of the OP microbiota in children with MPP. Microbial functions relateing to lipid metabolism, membrane transport and signal transduction were slightly enriched in children with MPP (Figure 5b). In contrast, the OP microbiota of healthy children was significantly enriched in orthologues involved in glycan biosynthesis and metabolism, biosynthesis of secondary metabolites, and cell growth and death (Figure 5b and Supplementary Table 2). Host homeostatis-associated functions, such as the immune system, digestive system, circulatory system and environmental adaptation, were also significantly abundant in the OP microbiota of healthy children (Figure 5b and Supplementary Table 2).

Characterization of the *M. pneumoniae* genome and 13 other reconstructed microbial genomes

We re-assembled 14 qualified microbial CAGs (Supplementary Table 3), representing the *M. pneumoniae* genome (0.80 Mbp) and the genomes of 13 other microorganisms (average genome size: 2.30 Mbp). The *M. pneumoniae* genome accumulated significantly in the OP microbiota of MPP patients and had high similarity with the reference genome (97.79% genome coverage; Supplementary Table 3). The *M. pneumoniae* genome included four antibiotic resistance genes (ARGs) against common antibiotics including peptide, rifamycin and fluoroquinolone (Figure 6, Supplementary Table 4). In eight patients who had been given experimental macrolides or beta-lactams (such as azithromycin, erythromycin or sulbactam), a SNP mutation, A2063G, which is related to macrolide resistance, was identified in the 23S rRNA (Supplementary Table 1). In addition, 136 virulence factor genes (VFGs) were found along its reassembled genome sequence (Supplementary Table 5), as well as redundant *M. pneumoniae* VFGs enriched in the secretion of adhesin P1, cytadherence protein and community-acquired respiratory distress syndrome (CARDS) toxin (Figure 6 and Supplementary Table 5).

Of the 13 other microbial genomes included in our study, five could be annotated

as specific species, one was annotated at the genus level (*Ralstonia*), and the remaining seven were novel microbial genomes (average genome size 1.74 Mbp) (Supplementary Table 3). For the five annotated microbial species, *S. aureus* and *S. epidermidis* increased significantly in MPP patients, while the other three *Prevotella spp.* mainly accumulated in healthy children (Figure 7, Supplementary Table 3). The largest reassembled *Ralstonia* genome (5.89 Mbp) carried numerous ARGs, including 13 beta-lactam ARGs, 21 tetracycline ARGs, and 11 macrolide ARGs. *P. histicola*, *P. shahii* and CAG00068 all had one copy each of a macrolide resistance gene and a beta-lactam antibiotic resistance gene. These genomes also harboured abundant VFG resources, ranging from 105 to 808 copies of relative genes. Correlation analysis revealed no significant correlation between these 14 reassembled microbial genomes and five clinical indices (Supplementary Table 6).

Discussion

Morbidity and mortality related to MPP is increasing in Chinese children. The development of RM studies has improved our understanding of the microbial

aetiology of MPP by revealing infection-associated RM imbalances [13, 14]. However, microbial functions and host–microbiota interactions in the RM of patients with MPP remain to be explored, particularly for novel microbial species.

In recent years, several reference gut microbial catalogues have been constructed to promote understanding of host–microorganism interactions. Qin et al. built a global view of the human gut microbiome (GM) and revealed a comprehensive functional potential of the prevalent gut microbial genes [21]. Li et al. upgraded the gut gene catalogue in 2014 [22], enabling studies to associate microbial genes with human health conditions. These frameworks have allowed researchers to deepen their understanding of the correlations between GM and various diseases, such as gastrointestinal and cardiovascular diseases [23, 24].

Like the reference gene catalogues that have been developed for the GM, our RMGC will further understanding of microbial aetiology in respiratory diseases. The development of a well-established RMGC is crucial for the functional metagenomics analysis needed to improve our knowledge of host–microbiota interactions in MPP. By aligning metagenomics data directly with the RMGC we have established,

researchers will be able to profile all microbial species, as well as explore microbial functions in both known and unknown microbial species. The fact that microbial assignment using the RMGC proved similar to that by 16S rRNA analysis also suggests that the RMGC may be promising for taxonomic studies using our constructed gene sets. Our data on the core microbial species of the OP microbiota in healthy children will provide a reference for exploring microbial and host–microorganism interactions in RM studies [25]. More generally, the RMGC provides a comprehensive respiratory-associated microbial profile to further microbiota analysis at the species level; functional profiling will facilitate further, more in-depth multi-omics analyses [26, 27], such as associations between proteins products and metabolites with known and novel microbial genomes. This capability would help to clarify interactions between the host and alterations to the RM during the progression of disease in MPP.

The OP microbiota of children with MPP has a simpler structure than that of healthy children. Previous studies have revealed that the bacteria-like *M. pneumoniae* is able to deplete levels of other bacteria through direct competition, and activates

bacterial clearance factors in host responses [28, 29]. This leads to decreased numbers colonising *Prevotella spp.*[30] and reduced proliferation of pathogens such as *S. aureus* and *S. epidermidis*. MPP patient-enriched genes have functions involved in membrane transport and the metabolism of various nutrients, which may partly explain the reduced tight junction proteins and increased respiratory mucosa permeability after infection [31]. Several studies have also identified increased glucose concentration in airway surface liquids [32–34] and associated pathogen proliferation [35]. This also corroborate the existence of enriched nutrient uptake pathways in the OP microbiota of children with MPP. Although the mechanism of *M. pneumoniae* clearance in the respiratory system remains unclear, these findings would provide new insights into host–microbiota interactions in MPP infection.

As well as a variety of well-known microorganisms, respiratory tracts also harbour numerous undiscovered microbial species [36]. Moreover, recent reports have demonstrated that a single bacterial genome can be recovered via reference gene sets and metagenomics data [37, 38]. It is difficult to culture *M. pneumoniae*, thus it is rarely done in clinical diagnosis, and this limits our understanding of antibiotic

resistance and virulence [39] in this species. Reconstruction of the *M. pneumoniae* genome using the RMGC and metagenomics data has indicated the existence of various ARGs related to RNA transcription [40], DNA replication [41] and protein synthesis [42]. According to clinical practice guidelines [43–45], most children with MPP are treated with azithromycin, erythromycin or sulbactam; none of these were associated with ARGs identified in the *M. pneumoniae* genome. Increasingly, reports demonstrate that specific dominant bacteria are associated with severe acute respiratory infections (ARIs) [6, 46, 47], but there have been no meaningful correlations identified between disease severity and *M. pneumonia* – or indeed between the other species of reassembled bacteria found in the OP microbiota of children with MPP. In our previous studies, we confirmed the succession of *M. pneumoniae* infection in NP to OP and lung, and determined an association between *M. pneumoniae* load in the lung microbiota with disease severity [14].

Although no macrolide/beta-lactam resistance genes were discovered in the *M. pneumoniae* genome, one SNP mutation (23S RNA, 2063A–>G) associated with macrolide resistance was identified in children with MPP. Meanwhile, in

patient-enriched microbial genomes such as *Ralstonia*, plenty of ARGs related to macrolide, beta-lactam and tetracycline resistance were found. Given rigorous antibiotic selective pressure and complex microbial interactions, environmentally redundant genetic components can be rapidly transferred into the pathogen genome by horizontal gene transfer [48, 49], causing the emergence of several diseases, such as happened in the European enterohemorrhagic *Escherichia coli* breakout [50] and the emergence of scarlet fever caused by *Streptococcus pyogenes* in Hong Kong [51]. Considering these findings, it should be recognised that current regimes for the treatment of *M. pneumoniae* hold the potential to trigger emerging drug-resistant strains, whether in *M. pneumoniae* or other novel microbial species. Indeed, macrolide resistance has already been reported in *M. pneumoniae*-PCR-positive children [52–54].

In OP microbiota samples from healthy children, several bacterial genomes were recovered, including the key player *Prevotella spp.* [55, 56], and several other novel microorganisms, such as *Vampirovibrio*, which might function as pathogen competitors [57]. Microbial genomes recovered from respiratory tracts hold the

potential to improve our understanding of the microbial aetiology in MPP pneumonia.

There are several limitations of this study. Given that there are currently no effective medicines for MPP, the patients in our study received empirical treatments that might have slightly shifted their airway ecology [58]. Despite promising applications for the RMGC, unclassified CAGs and novel gene families in the RMGC must be annotated and further explored. The copy numbers of several genes require further assessment because of the potential for inaccuracies caused by the low respiratory bacterial biomass, NGS sequencing and assembly methods. In this study, the respiratory microbial samples were obtained from Chinese children; therefore, like the continual updates made to the GM reference genes, metagenomics data from a more diverse sample will be incorporated into the RMGC in the future to more broadly characterise microbial components and functions [22, 59, 60]. This will incrementally improve studies of the imbalanced RM in patients with respiratory diseases. Alterations in the OP microbiota of Chinese patients with MPP will also provide extensive insights into the microbial aetiology of acute respiratory infection.

Potential implications

The respiratory microbial gene catalogue established here will help to deepen respiratory micro-ecology research, and has the potential to elucidate respiratory microbial communities at the microbial species level. In addition, by aligning metagenomics data with the reference catalogue, our work will facilitate assembly of the genomes of novel microbial genera or species, allow exploration of microbial functions and their associated microbial components, and allow the construction of whole microbial networks within the respiratory microbial community. Established reference gene sets can be employed to deepen multi-omics analysis, which will further our understanding of host–microorganism interactions in acute respiratory infection. One example of how these gene sets might be used is in the comparison of the OP microbiota of healthy and diseased children.

Methods

Clinical detection of infectious pathogens

Broncho-alveolar lavage fluid was used to identify common microorganisms of the microbiota. Culturing was conducted to detect *Streptococcus pneumoniae, H. influenzae, Moraxella catarrhalis, Staphylococcus aureus* and *Staphylococcus haemolyticus*. The D3 Ultra DFA Respiratory Virus Screening and ID Kit (Diagnostic

Hybrids, Inc., Athens, OH, USA) was used to detect common viruses, including adenovirus (AdV), respiratory syncytial virus (RSV), influenza virus and parainfluenza virus. Cytomegalovirus (CMV) and Epstein–Barr virus (EBV) were detected using the Diagnostic Kit for Quantification of Human CMV DNA and EBV Polymerase Chain Reaction (PCR) Fluorescence Quantitative Diagnostic Kit, respectively (DaAnGene, Guangzhou, China). *M. pneumoniae* and *Chlamydia pneumonia* were diagnosed using a diagnostic kit for *M. pneumoniae* DNA (PCR Fluorescence Probing; DaAnGene) and Anti *C. pneumoniae* ELISA (IgM) (EUROIMMUN AG, Lübeck, Germany), respectively.

Construction and annotation of the RMGC

Sequencing data were filtered using a previously reported method [61]. Samples with ≥650 Mbp data (Figure 1, Supplementary Figure 3) were selected for genome assembly using SOAPdenovo [62] (SOAPdenovo, RRID:SCR_010752, v2.07, –F –K 39 –M 3 –d 1). For samples with <650 Mbp data, data from the same respiratory site were mixed and assembled (Figure 1). Assembled contigs with ≥500 bps were

selected for gene prediction with MetaGeneMark [63] (v3.26, default parameters). We applied Glimmer3.02 [64] (Glimmer, RRID:SCR_011931, default parameters) to predict genes from the 1,384 respiratory bacterial genomes in the Integrated Microbial Genomes and Microbiomes (IMG) database (2016-12-21 [65]). Gene sequences were also retrieved from the genomes of 73 respiratory bacteria in the Pathosystems Resource Integration Center (PATRIC) database (2017-3-25 [66]) and 450,204 open reading frames (ORFs) of respiratory bacteria in Human Microbiome Project (HMP) (2016-10-20 [67]). Genes with a length ≥100 bp and without Ns (unidentified nucleotides) were selected to construct non-redundant gene sets using CD-HIT [68] (CD-HIT, RRID:SCR_007105, v4.66, -c 0.95 –aS 0.9). Genes with ≥2 mapped reads were retained in the established RMGC.

Taxonomic annotation of genes was conducted as follows:

i) Bacterial and viral genome sequences were retrieved from IMG (2016-12-21), PATRIC (2017-03-25) and National Center for Biotechnology Information (NCBI) NT database (2016-08-09 [69]);. The genome sequence with the longest N50 was selected as the representative

genome for each bacterial species. Non-redundant viral genomes were produced by CD-HIT (v4.66, -aS 0.95 -aL 0.9 -M 0). Gene sets in the RMGC were aligned to 6,869 representative bacterial genomes and 18,916 non-redundant viral DNA genomes using BLASTN (BLASTN, RRID:SCR_001598, v2.5.0, default parameters except –e 0.01)

ii) The top 10% highest-scoring alignments of each gene were retained, with ≥65% identity and ≥80% gene length coverage

iii) Assignment of each gene was determined based on ≥50% consensus above the similarity threshold for a specific rank: ≥65% for phylum, ≥85% for genus and ≥95% for species.

The functional annotation of each gene was determined by searching protein sequences in the Kyoto Encyclopedia of Genes and Genomes (KEGG) (v78.1) database and eggNOG (version 4.0) with BLASTP (BLASTP, RRID:SCR_001010, v2.5.0, default parameters, except -e 1e-5). The best-hit alignment (identity ≥30% and coverage ≥70%) was selected as the functional annotation for the gene. Genes without annotations in KEGG were identified as novel gene families by the Markov Cluster

Algorithm (MCL) [70] (inflation factor=1.1, bit-score cut-off=60).

Comparing taxonomic assessments of 16S rRNA gene analysis and metagenomic analysis

Seventy-two OP microbial samples with ≥650 Mb metagenomic sequencing data were aligned to establish the RMGC to determine taxonomic assignments. The same samples were also sequenced via the V3–V4 region of the 16S rRNA gene [13]. Microbial compositions obtained by these two methods were compared to assess the accuracy of taxonomic assignments via metagenomic analysis.

Rarefaction analysis

We downsized the number of mapped reads to 3 million per sample to eliminate variable influence caused by the quantity of sequencing data. Estimation of total gene richness was done by randomly sampling five individuals 1,000 times using gene counting and Chao2 richness estimator [71].

To produce rarefaction curves of KEGG orthologous groups (KOs) and novel

gene families, saturation was evaluated by randomly sampling five individuals 1,000 times. Relative rarefaction curves were visualized using R software (v3.3.2).

Calculation of gene relative abundance in RMGC

Filtered reads of metagenomics data from each sample were aligned to the RMGC using BWA (BWA, RRID:SCR_010910, v0.7.13, default parameters, except for the mem and identity ≥95%). Alignments meeting the following two criteria were accepted: i) paired-end reads mapped onto the same gene with the correct insert size; and ii) one end of a paired-reads was mapped onto the end of a gene, while the other was located outside of the gene.

If the number of genes in a given sample was n, the relative abundance was calculated using the following steps:

1. The copy number of the gene i ($c(i)$) was calculated as:

$$c(i) = \frac{t(i)}{l(i)}$$

where $t(i)$ is the total number of mapped reads of gene i in a given sample, and $l(i)$ is the length of the gene i.

2. The relative abundance of gene *i (Ab_g(i))* was defined as:

$$Ab\_g(i) = \frac{c(i)}{\sum_{1}^{n} c(i)}$$

3. If m genes can be assigned to the phylogenetic assignment s, the abundance of this phylogenetic assignment *(Ab_p(s))* was calculated using the following equation:

$$Ab\_p(s) = \sum_{1}^{m} Ab\_g(j)$$

Phylogenetic and functional profile of the OP microbiome

All filtered reads of the OP microbiota were aligned to the RMGC using BWA with same parameters as described above. The relative abundance of each phylogenetic assignment was calculated as shown above, while the abundance of KOs in the functional profiling table was determined as described in a previous report [59].

Identification of core OP microbial species in healthy children

Microbial species were selected as ‘core’ species if they existed in more than 50% of healthy children and had more than 1% relative abundance in one OP microbial sample. Distributions of core microbial species in the OP of healthy children were

described using ggplot2 in R.

Comparison of the OP microbiota between healthy children and children with MPP

Thirty-three healthy children were chosen, with a similar age distribution to that of the 34 children with MPP (data size ≥650 Mbp). Genes in the OP microbiota of selected microbial samples were clustered into co-abundance gene groups (CAGs) via Capony-based algorithms [72] (default parameters). Selected CAGs containing more than 700 genes were regarded as being derived from the same bacterial genome and were selected to construct a correlation network using Spearman's rank coefficient (≤–0.6 or ≥0.6). The co-occurrence network was visualized using Cytoscape (v3.4.0) [73]. If ≥50% of the included genes had consensus phylogenetic annotations, the corresponding CAG was assigned to a related microbial taxonomic assignment.

The relative abundance of each CAG in our microbial samples was calculated as previously reported [60]. Intergroup comparisons between CAGs and KEGG functions were performed using the two-tailed Wilcoxon rank-sum test and corrected

via the Benjamini-Hochberg method (adjusted *P*-value ≤0.05). Confounding factors including pneumonia, sex, age, delivery mode and feed pattern were also assessed using permutational analysis of variance (PERMANOVA) by vegan package (v2.3-4) in R.

Single microbial genome assembly from OP metagenomic data

OP metagenomic data were aligned to the filtered CAGs (those containing ≥700 genes) by BWA (v0.7.13, identity ≥95%). The mapped reads of each CAG were extracted for microbial genome assembly with Velvet [74] (Velvet, RRID:SCR_010755, kmer: from 45 to 75, cov_cutoff: auto, exp_cov: auto). The assembled sequences with the longest contig N50 were selected as representative draft genomes. Assembly quality was assessed using the following six criteria [24]: (i) 90% of the genome assembly included in contigs >500 bp; (ii) 90% of the assembled bases at >5× read coverage; (iii) contig N50 >5 kb; (iv) scaffold N50 >20 kb; (v) average contig length >5 kb; and (vi) >90% of core genes present in the assembly. Fourteen draft microbial genomes passed five or six of these criteria (Supplementary Table 3). The assembly quality

estimation standard published by the Genomic Standards Consortium (GSC) was then applied (Supplementary Table 3) [75]. The microbial species designation of 14 assembled genome sequences followed these standards: 1) concordance with taxonomical assignment of CAGs [72]; 2) aligned to the genome sequences published by IMG, NCBI and PATRIC via BLASTN (v2.5.0, default parameters except –e 0.01), with ≥95% nucleotide identity and ≥95% genome coverage; and 3) assigned by CheckM (CheckM, RRID:SCR_016646, v1.0.12, default parameters) from the Genome Taxonomy database [76]. Furthermore, gene prediction was executed with Glimmer3.02 (Glimmer, RRID:SCR_011931), while related annotations of antibiotic resistance and virulence genes were acquired through CARD [77] and VFDB [78]. The SNP mutation associated with macrolide resistance of *M. pneumoniae* was identified by mapping sequencing reads against 23S rRNA genes [79] using BWA.

Correlations between reassembled microbial genomes and disease severity in MPP patients

The correlation between reconstructed microbial genomes and hospitalization

duration and fever peak was assessed using R software, a well as serum CRP, PCT and eosinophil levels at 24 hours after hospitalization. The distributions of relative abundance of 14 reassembled genomes in children ith MPP and healthy children were showed via scatter plot.

Availability of supporting data and materials

The BioProject ID for this study is PRJNA413615. The sequencing data supporting the results of this article are available in the GenBank repository under accession number SRP119571. The gene profiles are freely accessible on RMGC database [20].

All supporting data and materials are available in the *GigaScience* GigaDB database [80].

Declarations

List of abbreviations

AdV, Adenovirus; ARI, Acute respiratory infection; BALF, Broncho-alveolar lavage fluid; CAG, Co-abundance gene group; CMV, Cytomegalovirus; EBV, Epstein–Barr

virus; GM, Gut microbiome; KEGG: Kyoto Encyclopedia of Genes and Genomes; KO, KEGG orthologous group; MCL, Markov cluster algorithm; NP, Nasopharynx; OP, Oropharynx; ORF, Open reading frame; PCA, Principal component analysis; PCR, Polymerase chain reaction; PERMANOVA, Permutational multivariate analysis of variance analysis; PP, Paediatric pneumonia; RM, Respiratory microbiome; RMGC, Respiratory microbial gene catalogue; RSV, Respiratory syncytial virus.

Ethics approval

Ethical approval for this study was obtained from the Ethical Committee of Shenzhen Children's Hospital (Shenzhen, Guangdong Province, China) under registration number 2016013. All experiments were performed under the relevant guidelines and regulations. Guardians of all children included in this study provided their informed consent to participate.

Consent for publication

All authors have given their consent to publish the manuscript.

Competing interests

The authors declare no that they have no competing interests.

Funding

This study was supported by grants from the Key Medical Disciplines Building Project of Shenzhen (grant number SZXJ2017005), the Sanming Project of Medicine in Shenzhen (grant number SZSM201512030), the Shenzhen Science and Technology Project (grant numbers JCYJ20170303155012371 and JCYJ20170816170527583), and the Guangdong Medical Research Fund (grant number A2017213).

Author contributions

Y.Z., Y.Y. and K.Z. managed the project. Z.L., G.X. and Y.B. collected samples and information. W.W. and Q.Y. prepared the DNA extraction. D.L., Q.Z., X.F. and Z.Y. performed the bioinformatics analysis. C.Q., Y.L. and Y.L. optimized the graphs. X.X. and M.L. optimized the data curation. S.L. and Y.Y. guided data interpretation. X.F.

developed the website. H.W. and W.D. mined the data and wrote the paper. K.S. and K.Y. polished the article. All authors read and approved the final version of the manuscript.

Acknowledgements

We thank members of the Collaborating Group of Pediatric Respiratory Microbiome, Chinese Pediatric Society and Chinese Medical Association, for their suggestions.

Author information

Y.Y. is a Russian academician of paediatric and vaccine research. Y.Z is Director of the Respiratory Disease Department at Shenzhen Children’s Hospital, China. S.L is a professor in the Department of Computer Science at the City University of Computer Science, Hong Kong. K.Z is a professor at Wuhan National Laboratory for Optoelectronics, Huazhong University of Science and Technology, China.

References

1. Stearns JC, Davidson CJ, McKeon S, Whelan FJ, Fontes ME, Schryvers AB, *et al.* Culture and molecular-based profiles show shifts in bacterial communities of the upper respiratory tract that occur with age. ISME J. 2015; 9**:** 1268.

2. Biesbroek G, Tsivtsivadze E, Sanders EA, Montijn R, Veenhoven RH, Keijser BJ*, et al.* Early respiratory microbiota composition determines bacterial succession patterns and respiratory health in children. Am J Respir Crit Care Med. 2014; 190**:** 1283-92.

3. Biesbroek G, Bosch AA, Wang X, Keijser BJ, Veenhoven RH, Sanders EA*, et al.* The impact of breastfeeding on nasopharyngeal microbial communities in infants. Am J Respir Crit Care Med. 2014; 190**:** 298-308.

4. Bosch AA, de Steenhuijsen Piters WA, van Houten MA, Chu M, Biesbroek G, Kool J*, et al.* Maturation of the infant respiratory microbiota, environmental drivers and health consequences: a prospective cohort study. Am J Respir Crit Care Med. 2017; 196**:** 1582-90.

5. Charlson ES, Bittinger K, Haas AR, Fitzgerald AS, Frank I, Yadav A*, et al.*

Topographical continuity of bacterial populations in the healthy human respiratory tract. Am J Respir Crit Care Med. 2011; 184**:** 957-63.

6. de Steenhuijsen Piters WA, Huijskens EG, Wyllie AL, Biesbroek G, van den Bergh MR, Veenhoven RH, *et al.* Dysbiosis of upper respiratory tract microbiota in elderly pneumonia patients. ISME J. 2016; 10**:** 97-108.

7. Sakwinska O, Bastic Schmid V, Berger B, Bruttin A, Keitel K, Lepage M, *et al.* Nasopharyngeal microbiota in healthy children and pneumonia patients. J Clin Microbiol. 2014; 52**:** 1590-4.

8. Prina E, Ranzani OT, Torres A. Community-acquired pneumonia. Lancet. 2015; 386**:** 1097-108.

9. Musher DM, Thorner AR. Community-acquired pneumonia. N Engl J Med. 2014; 371**:** 1619-28.

10. Liu L, Oza S, Hogan D, Perin J, Rudan I, Lawn JE, *et al.* Global, regional, and national causes of child mortality in 2000-13, with projections to inform post-2015 priorities: an updated systematic analysis. Lancet. 2015; 385**:** 430-40.

11. Dagan R, Bhutta ZA, de Quadros CA, Garau J, Klugman KP, Khuri-Bulos N, *et al.* The remaining challenge of pneumonia: the leading killer of children. Pediatr Infect Dis J. 2011; 30**:** 1-2.

12. Qin Q, Baoping Xu, Liu X, Shen K. Status of Mycoplasma pneumoniae pneumonia in chinese children: a systematic review. Advances in Microbiology. 2014; 4**:** 704-11.

13. Lu Z, Dai W, Liu Y, Zhou Q, Wang H, Li D, *et al.* The alteration of nasopharyngeal and oropharyngeal microbiota in children with MPP and non-MPP. Genes (Basel). 2017; 8.

14. Dai W, Wang H, Zhou Q, Feng X, Lu Z, Li D, *et al.* The concordance between upper and lower respiratory microbiota in children with Mycoplasma pneumoniae pneumonia. Emerg Microbes Infect. 2018; 7**:** 92.

15. Hasegawa K, Mansbach JM, Ajami NJ, Espinola JA, Henke DM, Petrosino JF, *et al.* Association of nasopharyngeal microbiota profiles with bronchiolitis severity in infants hospitalised for bronchiolitis. Eur Respir J. 2016; 48**:** 1329-39.

16. de Steenhuijsen Piters WA, Heinonen S, Hasrat R, Bunsow E, Smith B, Suarez-Arrabal MC, *et al.* Nasopharyngeal microbiota, host transcriptome, and disease severity in children with respiratory syncytial virus infection. Am J Respir Crit Care Med. 2016; 194**:** 1104-15.

17. Pettigrew MM, Gent JF, Kong Y, Wade M, Gansebom S, Bramley AM, *et al.* Association of sputum microbiota profiles with severity of community-acquired pneumonia in children. BMC Infect Dis. 2016; 16**:** 317.

18. Vissing NH, Chawes BL, Bisgaard H. Increased risk of pneumonia and bronchiolitis after bacterial colonization of the airways as neonates. Am J Respir Crit Care Med. 2013; 188**:** 1246-52.

19. Mika M, Mack I, Korten I, Qi W, Aebi S, Frey U, *et al.* Dynamics of the nasal microbiota in infancy: a prospective cohort study. J Allergy Clin Immunol. 2015; 135**:** 905-12.e11.

20. The Respiratory Microbial Gene Catalogue database. https://rmgc.deepomics.org. Accessed 2017

21. Zhang R, Wang H, Deng J. A 4-Year-Old Girl With Progressive Cough and

Abnormal Blood Smear. Clinical Infectious Diseases. 2017; 64**:** 1630–31.

22. Li J, Jia H, Cai X, Zhong H, Feng Q, Sunagawa S, *et al.* An integrated catalog of reference genes in the human gut microbiome. Nat Biotechnol. 2014; 32**:** 834-41.

23. Qin J, Li Y, Cai Z, Li S, Zhu J, Zhang F, *et al.* A metagenome-wide association study of gut microbiota in type 2 diabetes. Nature. 2012; 490**:** 55-60.

24. Zhang C, Yin A, Li H, Wang R, Wu G, Shen J, *et al.* Dietary modulation of gut microbiota contributes to alleviation of both genetic and simple obesity in children. EBioMedicine. 2015; 2**:** 968-84.

25. Rosas-Salazar C, Shilts MH, Tovchigrechko A, Schobel S, Chappell JD, Larkin EK, *et al.* Differences in the nasopharyngeal microbiome during acute respiratory tract infection with human rhinovirus and respiratory syncytial virus in infancy. J Infect Dis. 2016; 214**:** 1924-28.

26. Stewart CJ, Mansbach JM, Wong MC, Ajami NJ, Petrosino JF, Camargo CAJ, *et al.* Associations of nasopharyngeal metabolome and microbiome with severity among infants with bronchiolitis: a multi-omic analysis. Am J Respir

Crit Care Med. 2017; 196: 882-91.

27. Quinn RA. Integrating microbiome and metabolome data to understand infectious airway disease. Am J Respir Crit Care Med. 2017; 196: 806-07.

28. Yang J, Hooper WC, Phillips DJ, Talkington DF. Cytokines in Mycoplasma pneumoniae infections. Cytokine Growth Factor Rev. 2004; 15: 157-68.

29. Peteranderl C, Sznajder JI, Herold S, Lecuona E. Inflammatory responses regulating alveolar ion transport during pulmonary infections. Front Immunol. 2017; 8: 446.

30. Miller SI, Ernst RK, Bader MW. LPS, TLR4 and infectious disease diversity. Nat Rev Microbiol. 2005; 3: 36-46.

31. Patkee WR, Carr G, Baker EH, Baines DL, Garnett JP. Metformin prevents the effects of Pseudomonas aeruginosa on airway epithelial tight junctions and restricts hyperglycaemia-induced bacterial growth. J Cell Mol Med. 2016; 20: 758-64.

32. Hewitt R, Webber J, Farne H, Trujillo-Torralbo M-B, Footitt J, Molyneaux PL, *et al.* Airway glucose in virus-induced COPD exacerbations. Am J Respir Crit

Care Med. 2016; 192**:** A6323.

33. Garnett JP, Nguyen TT, Moffatt JD, Pelham ER, Kalsi KK, Baker EH, *et al.* Proinflammatory mediators disrupt glucose homeostasis in airway surface liquid. J Immunol. 2012; 189**:** 373-80.

34. Kalsi KK, Baker EH, Fraser O, Chung YL, Mace OJ, Tarelli E, *et al.* Glucose homeostasis across human airway epithelial cell monolayers: role of diffusion, transport and metabolism. Pflugers Arch. 2009; 457**:** 1061-70.

35. Philips BJ, Redman J, Brennan A, Wood D, Holliman R, Baines D, *et al.* Glucose in bronchial aspirates increases the risk of respiratory MRSA in intubated patients. Thorax. 2005; 60**:** 761-4.

36. Man WH, de Steenhuijsen Piters WA, Bogaert D. The microbiota of the respiratory tract: gatekeeper to respiratory health. Nat Rev Microbiol. 2017; 15**:** 259-70.

37. Ji P, Zhang Y, Wang J, Zhao F. MetaSort untangles metagenome assembly by reducing microbial community complexity. Nat Commun. 2017; 8**:** 14306.

38. Olm MR, Brown CA-O, Brooks B, Banfield JF. dRep: a tool for fast and

accurate genomic comparisons that enables improved genome recovery from metagenomes through de-replication. ISME J. 2017; 11**:** 2864-68.

39. Saraya T, Kurai D, Nakagaki K, Sasaki Y, Niwa S, Tsukagoshi H, *et al.* Novel aspects on the pathogenesis of Mycoplasma pneumoniae pneumonia and therapeutic implications. Front Microbiol. 2014; 5**:** 410.

40. Floss HG, Yu TW. Rifamycin-mode of action, resistance, and biosynthesis. Chem Rev. 2005; 105**:** 621-32.

41. Nesar S, MH. S, Rahim N, Rehman R. Emergence of resistance to fluoroquinolones among gram positive and gram negative clinical isolates. Pak J Pharm Sci. 2012; 25**:** 877-81.

42. Axelsen PH. A chaotic pore model of polypeptide antibiotic action. Biophys J. 2008; 94**:** 1549-50.

43. Harris M, Clark J, Coote N, Fletcher P, Harnden A, McKean M, *et al.* British Thoracic Society guidelines for the management of community acquired pneumonia in children: update 2011. Thorax. 2011; 66 Suppl 2**:** ii1-23.

44. Bradley JS, Byington CL, Shah SS, Alverson B, Carter ER, Harrison C, *et al.*

The management of community-acquired pneumonia in infants and children older than 3 months of age: clinical practice guidelines by the Pediatric Infectious Diseases Society and the Infectious Diseases Society of America. Clin Infect Dis. 2011; 53**:** e25-76.

45. Lee H, Yun KW, Lee HJ, Choi EH. Antimicrobial therapy of macrolide-resistant Mycoplasma pneumoniae pneumonia in children. Expert Rev Anti Infect Ther. 2018; 16**:** 23-34.

46. Hasegawa K, Mansbach JM, Ajami NJ, Espinola JA, Henke DM, Petrosino JF, *et al.* Association of nasopharyngeal microbiota profiles with bronchiolitis severity in infants hospitalised for bronchiolitis. Eur Respir J. 2016; 48**:** 1329-39.

47. Hasegawa K, Linnemann RW, Mansbach JM, Ajami NJ, Espinola JA, Petrosino JF, *et al.* Nasal airway microbiota profile and severe bronchiolitis in infants: a case-control study. Pediatr Infect Dis J. 2017; 36**:** 1044-51.

48. Citti C, Dordet-Frisoni E, Nouvel LX, Kuo CH, Baranowski E. Horizontal gene transfers in Mycoplasmas (Mollicutes). Curr Issues Mol Biol. 2018; 29**:**

3-22.

49. Xiao L, Ptacek T, Osborne JD, Crabb DM, Simmons WL, Lefkowitz EJ, *et al.* Comparative genome analysis of Mycoplasma pneumoniae. BMC Genomics. 2015; 16**:** 610.

50. Rohde H, Qin J, Cui Y, Li D, Loman NJ, Hentschke M, *et al.* Open-source genomic analysis of Shiga-toxin-producing E. coli O104:H4. N Engl J Med. 2011; 365**:** 718-24.

51. Davies MR, Holden MT, Coupland P, Chen JH, Venturini C, Barnett TC, *et al.* Emergence of scarlet fever Streptococcus pyogenes emm12 clones in Hong Kong is associated with toxin acquisition and multidrug resistance. Nat Genet. 2015; 47**:** 84-7.

52. Kutty PK, Jain S, Taylor TH, Bramley AM, Diaz MH, Ampofo K, *et al.* Mycoplasma pneumoniae among children hospitalized with community-acquired pneumonia. Clin Infect Dis. 2019; 68**:** 5-12.

53. Blyth CC, Gerber JS. Macrolides in children with community-acquired pneumonia: panacea or placebo? J Pediatric Infect Dis Soc. 2018; 7**:** 71-77.

54. Yang D, Chen L, Chen ZA-O. The timing of azithromycin treatment is not associated with the clinical prognosis of childhood Mycoplasma pneumoniae pneumonia in high macrolide-resistant prevalence settings. PLoS One. 2018; 13**:** e0191951.

55. Larsen JM, Musavian HS, Butt TM, Ingvorsen C, Thysen AH, Brix S. Chronic obstructive pulmonary disease and asthma-associated Proteobacteria, but not commensal Prevotella spp., promote Toll-like receptor 2-independent lung inflammation and pathology. Immunology. 2015; 144**:** 333-42.

56. Segal LN, Clemente JC, Tsay JC, Koralov SB, Keller BC, Wu BG, *et al.* Enrichment of the lung microbiome with oral taxa is associated with lung inflammation of a Th17 phenotype. Nat Microbiol. 2016; 1**:** 16031.

57. de Dios Caballero J, Vida R, Cobo M, Maiz L, Suarez L, Galeano J, *et al.* Individual patterns of complexity in cystic fibrosis lung microbiota, including predator bacteria, over a 1-year period. MBio. 2017; 8**:** e00959-17.

58. Maier L, Pruteanu M, Kuhn M, Zeller G, Telzerow A, Anderson EE, *et al.* Extensive impact of non-antibiotic drugs on human gut bacteria. Nature. 2018;

555**:** 623-28.

59. Qin J, Li R, Raes J, Arumugam M, Burgdorf KS, Manichanh C, *et al.* A human gut microbial gene catalogue established by metagenomic sequencing. Nature. 2010; 464**:** 59-65.

60. Lloyd-Price J, Mahurkar A, Rahnavard G, Crabtree J, Orvis J, Hall AB, *et al.* Strains, functions and dynamics in the expanded Human Microbiome Project. Nature. 2017; 550**:** 61-66.

61. Yan L, Yang M, Guo H, Yang L, Wu J, Li R, *et al.* Single-cell RNA-Seq profiling of human preimplantation embryos and embryonic stem cells. Nat Struct Mol Biol. 2013; 20**:** 1131-9.

62. Luo R, Liu B, Xie Y, Li Z, Huang W, Yuan J, *et al.* SOAPdenovo2: an empirically improved memory-efficient short-read de novo assembler. Gigascience. 2012; 1**:** 18.

63. Zhu W, Lomsadze A, Borodovsky M. Ab initio gene identification in metagenomic sequences. Nucleic Acids Res. 2010; 38**:** e132.

64. Delcher AL, Bratke KA, Powers EC, Salzberg SL. Identifying bacterial genes

and endosymbiont DNA with Glimmer. Bioinformatics. 2007; 23**:** 673-9.

65. Integrated Microbial Genomes and Microbiomes (IMG) database. https://img.jgi.doe.gov/. Accessed 21 Dec 2016.

66. Pathosystems Resource Integration Center (PATRIC) database. https://www.patricbrc.org/. Accessed 25 Mar 2017.

67. Human Microbiome Project (HMP). https://portal.hmpdacc.org/. Accessed 20 Oct 2016.

68. Li W, Godzik A. Cd-hit: a fast program for clustering and comparing large sets of protein or nucleotide sequences. Bioinformatics. 2006; 22**:** 1658-9.

69. National Center for Biotechnology Information (NCBI) NT database. ftp://ftp.ncbi.nlm.nih.gov/. Accessed 17 Aug 2016.

70. Enright AJ, Van Dongen S, Ouzounis CA. An efficient algorithm for large-scale detection of protein families. Nucleic Acids Res. 2002; 30**:** 1575-84.

71. Chao A. Estimating the population size for capture-recapture data with unequal catchability. Biometrics. 1987; 43**:** 783-91.

72. Nielsen HB, Almeida M, Juncker AS, Rasmussen S, Li J, Sunagawa S, *et al.* Identification and assembly of genomes and genetic elements in complex metagenomic samples without using reference genomes. Nat Biotechnol. 2014; 32: 822-8.

73. Shannon P, Markiel A, Ozier O, Baliga NS, Wang JT, Ramage D, *et al.* Cytoscape: a software environment for integrated models of biomolecular interaction networks. Genome Res. 2003; 13: 2498-504.

74. Zerbino DR, Birney E. Velvet: algorithms for de novo short read assembly using de Bruijn graphs. Genome Res. 2008; 18: 821-9.

75. Bowers RM, Kyrpides NC, Stepanauskas R. Minimum information about a single amplified genome (MISAG) and a metagenome-assembled genome (MIMAG) of bacteria and archaea. 2017; 35: 725-31.

76. Parks DH, Chuvochina M, Waite DW, Rinke C, Skarshewski A, Chaumeil PA, *et al.* A standardized bacterial taxonomy based on genome phylogeny substantially revises the tree of life. Nat Biotechnol. 2018; 36: 996-1004.

77. Jia B, Raphenya AR, Alcock B, Waglechner N, Guo P, Tsang KK, *et al.* CARD

2017: expansion and model-centric curation of the comprehensive antibiotic resistance database. Nucleic Acids Res. 2017; 45: D566-D73.

78. Chen L, Zheng D, Liu B, Yang J, Jin Q. VFDB 2016: hierarchical and refined dataset for big data analysis--10 years on. Nucleic Acids Res. 2016; 44: D694-7.

79. Ji M, Lee NS, Oh JM, Jo JY, Choi EH, Yoo SJ, *et al.* Single-nucleotide polymorphism PCR for the detection of Mycoplasma pneumoniae and determination of macrolide resistance in respiratory samples. J Microbiol Methods. 2014; 102: 32-6.

80. Dai W; Wang H; Li D; Zhou Q; Feng X; Yang Z; Wang W; Qiu C; Lu Z; Xu X; Lyu M; Xie G; Li Y; Bao Y; Liu Y; Shen K; Yao K; Feng X; Yang Y; Li S; Zhou K; Zheng Y (2019): Supporting data for "The integrated respiratory microbial gene catalogue facilitate the understanding of microbial aetiology in Mycoplasma pneumoniae pneumonia" GigaScience Database. http://dx.doi.org/10.5524/100620

Tables

Table 1. Sample information

**Characteristics**	**Pneumonia patients (n=76)**	**Healthy children (n=171)**
**Gender**		
Female	24	87
Male	52	84
**Age (years)**	2.9 (0.2–12.7)	4.3 (0.1–8.9)
**Sampling site**		
OP	75	171
NP	42	-
Lung	46	-
**Delivery mode**		
Vaginally born	46	102
Caesarean section	30	69
**Feeding pattern**		
Breast	48	84
Breast + milk	12	66
Milk	16	21
**Family history of allergy**	1	-
**History of pneumonia**	14	-
**Asthma**	-	-
**Clinical symptoms**		
Lung consolidation, atelectasis, infiltration	76	NA
Fever	44	-
Cough	72	-
Wheezing	20	-
Hospitalization time (days)	9 (2–37)	-
CRP (<0.499 mg/l)	22	NA

PCT (<0.5 ng/ml)	73	NA
Eosinophils (0.5–5%)	44	NA

-, no detection result; NA, not available; CRP, C-response protein; PCT, procalcitonin

Figure Legends

Figure 1. **Construction of the human Respiratory Microbial Gene Catalogue**.

Genome assembly was performed for each sample with ≥650 Mbp of data. For samples with <650 Mbp of data, data from the same respiratory site (NP, OP or lung) were mixed and assembled. Gene predictions were conducted for all assembled contigs with ≥500 bp and respiratory bacterial genomes in the Integrated Microbial Genomes and Microbiomes (IMG) database. Genes with ≥100 bp were retained. Respiratory gene sets from the Human Microbiome Project (HMP) and Pathosystems Resource Integration Center (PATRIC) were combined to construct the non-redundant Respiratory Microbial Gene Catalogue (RMGC) containing 2,245,343 genes.

Figure 2. Rarefaction curves for genes and KEGG orthologous groups/gene families.

a) Rarefaction curve for gene count.

b) Rarefaction curve for Chao2. The Respiratory Microbial Gene Catalogue (RMGC) captured 90.52% of the prevalent genes.

c) Rarefaction curve for KEGG (Kyoto Encyclopedia of Genes and Genomes) orthologous groups/gene families. Known functions saturate quickly to 6,346 groups. After including novel gene families, the rarefaction curve plateaus when 12,924 groups are detected. Boxes represent the interquartile ranges (IQRs) between the first and third quartiles and the line inside the box represents the median value. Whiskers represent the lowest or highest values within values 1.5 times the IQR from the first or third quartiles. Circles represent data points located outside of the whiskers.

Figure 3. Core microbial species in healthy OP microbiota.

Upper bar plot represents the prevalence of each core species; lower box plot shows the relative abundance of each core species. Different colours refer to different phyla.

Figure 4. Differentiation of oropharyngeal microbial samples between healthy

children and children with *Mycoplasma pneumoniae* pneumonia.

a) Gene counts in the oropharyngeal (OP) microbiotas of healthy children and children with *Mycoplasma pneumoniae* pneumonia (MPP).

b) Alpha diversity of the OP microbiota in healthy children and children with MPP. Boxes represent the interquartile ranges (IQRs) between the first and third quartiles and the line inside the box represents the median. Whiskers represent the lowest or highest values within values 1.5 times the IQR from the first or third quartiles. Points represent data located outside of the whiskers. *** $P \leq 0.001$.

Figure 5. Phylogenetic and functional alterations in children with pneumonia.

a) Size of the circle represents the average relative abundance of co-abundance gene groups (CAGs) in healthy children or children with *Mycoplasma pneumoniae* pneumonia (MPP). A line between two circles indicates a Spearman's rank correlation coefficient ≥0.6 and an adjusted *P*-value ≤0.05. The phylum and genus corresponding to each CAG are indicated by the information listed on the left.

b) X-axis represents level 2 functional categories in the Kyoto Encyclopedia of Genes

and Genomes (KEGG); colours of the characters represent level 1 functional categories (listed on the right) Y-axis shows the relative abundance of level 2 functional categories. Adjusted P-values at *, ≤0.05; **, ≤0.01; and ***, ≤0.001, respectively.

Figure 6. Virulence factor genes and antibiotic resistance genes on the *Mycoplasma pneumoniae* genome.

Tracks from outside to inside represent antibiotic resistance genes (ARGs), genes on the plus strand, genes on the negative strand and GC skew, respectively. Virulence factor genes (VFGs) painted with different colours refer to the different types of VFGs.

Figure 7. Comparison of relative abundance of 14 re-assembled genomes between healthy children and children with *Mycoplasma pneumoniae* pneumonia.

Blue circles and red triangles represent the microbial relative abundance of healthy children and children with *Mycoplasma pneumoniae* pneumonia (MPP)**.** Solid dot and

paired whiskers represent the mean and SD of each microbial relative abundance. P-values at *, ≤0.05; **, ≤0.01; and ***, ≤0.001, respectively. NS, not significant.

Figure 1

Click here to access/download;Figure;Figure 1.pdf

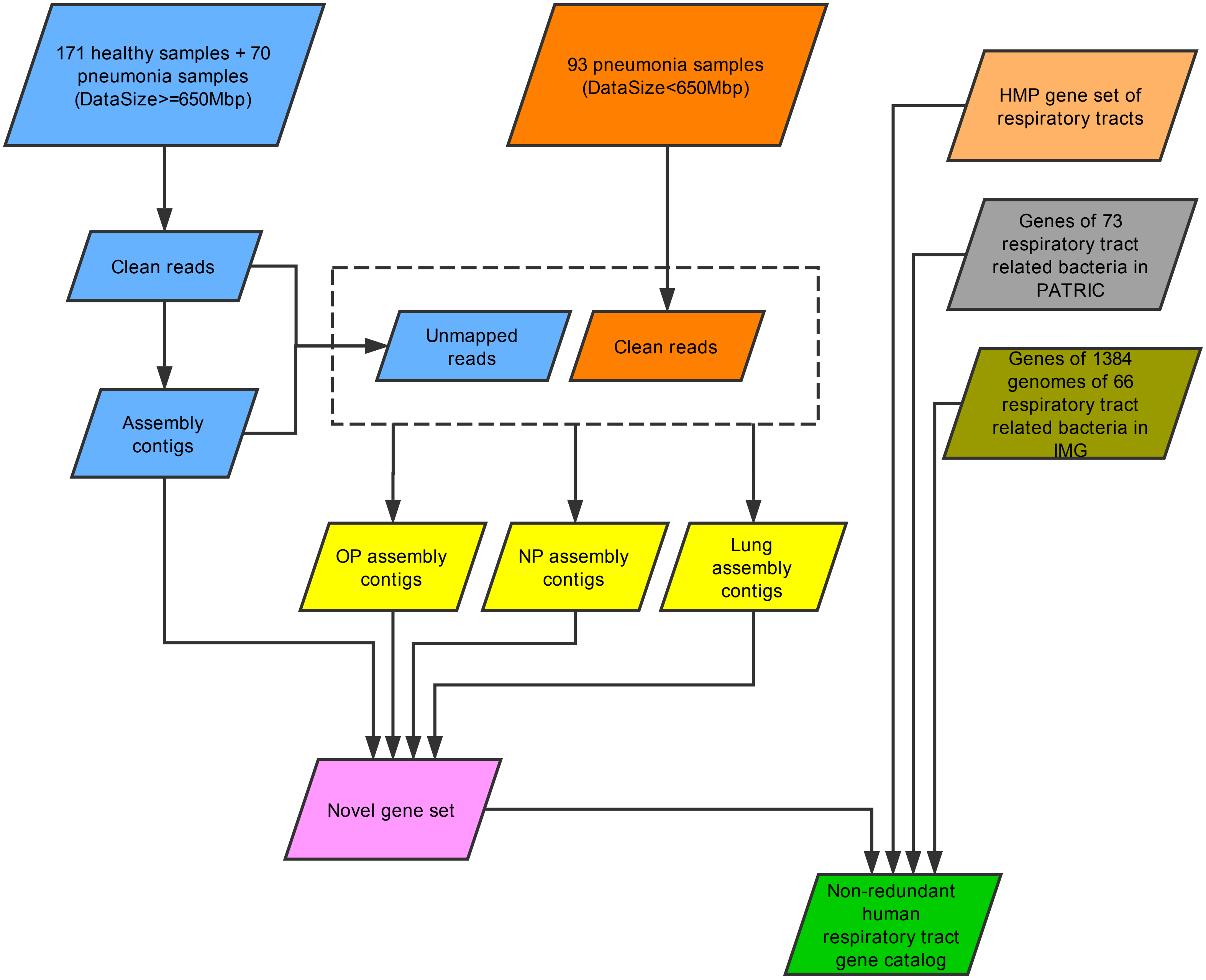

Figure 2

Click here to access/download;Figure;Figure 2.pdf

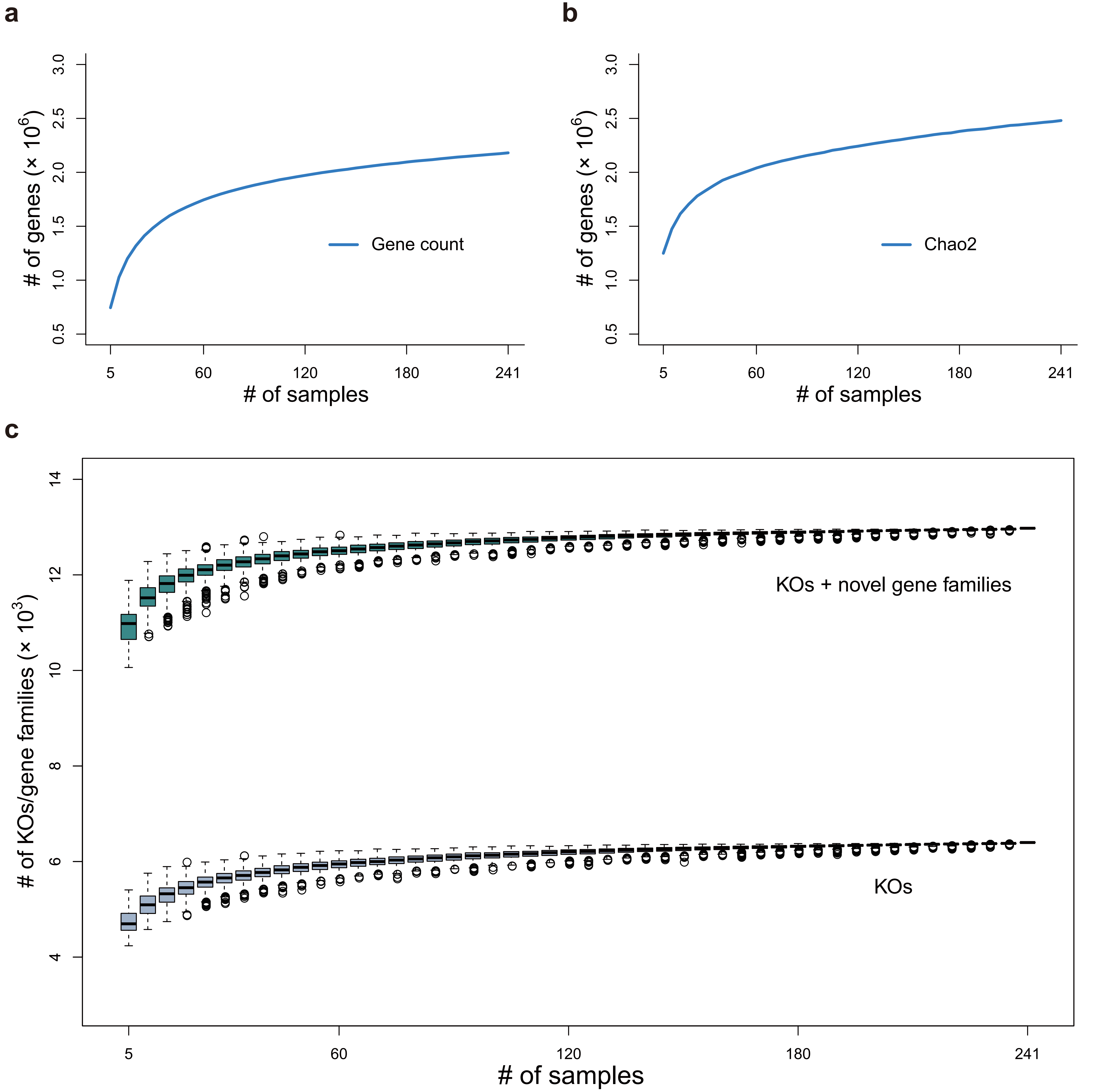

Figure 3

Click here to access/download;Figure;Figure 3.pdf

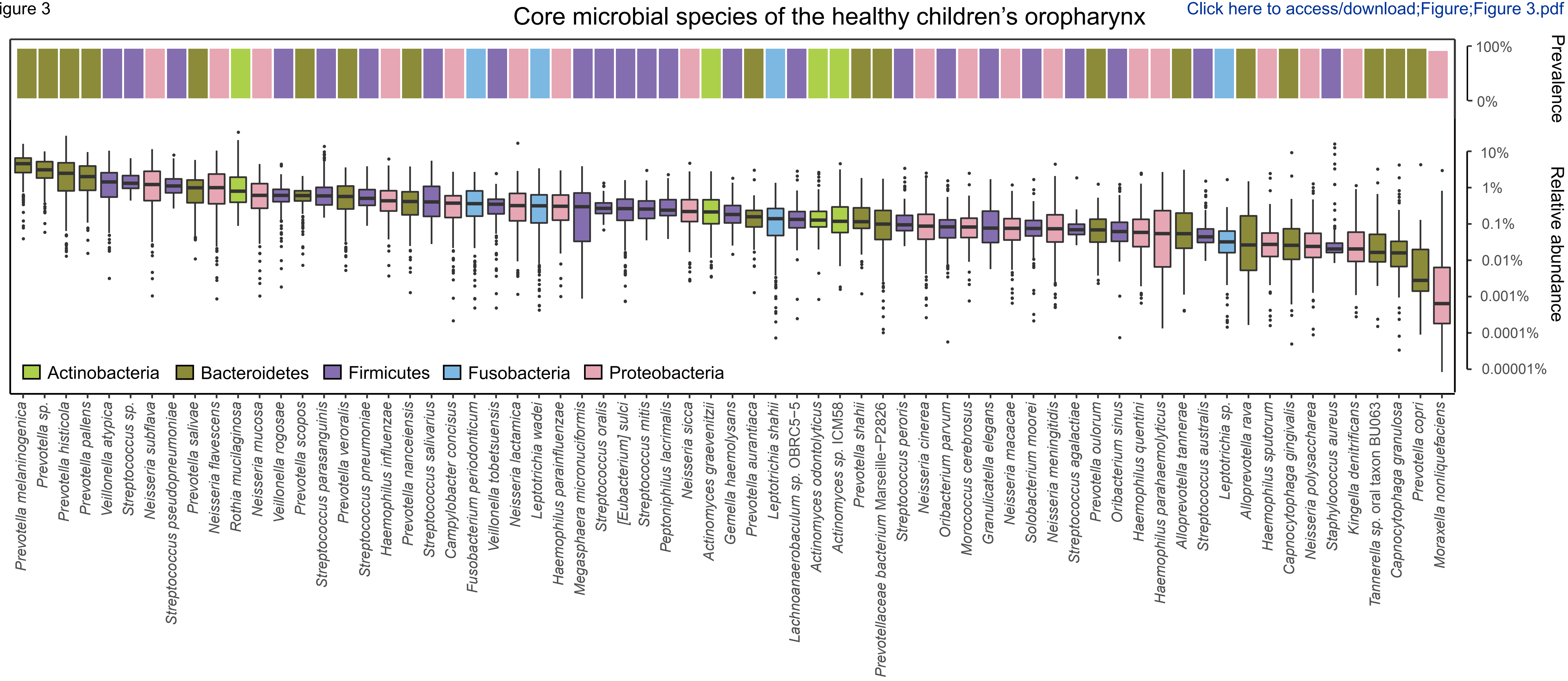

Figure 4

Click here to access/download;Figure;Figure 4.pdf

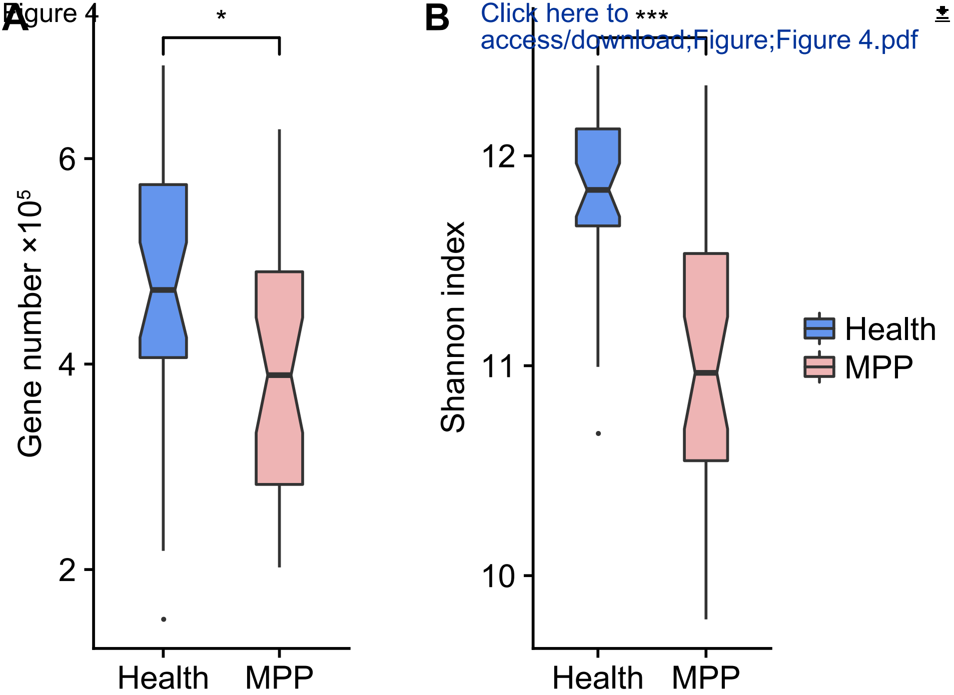

Figure 5

Click here to access/download;Figure;Figure 5.pdf

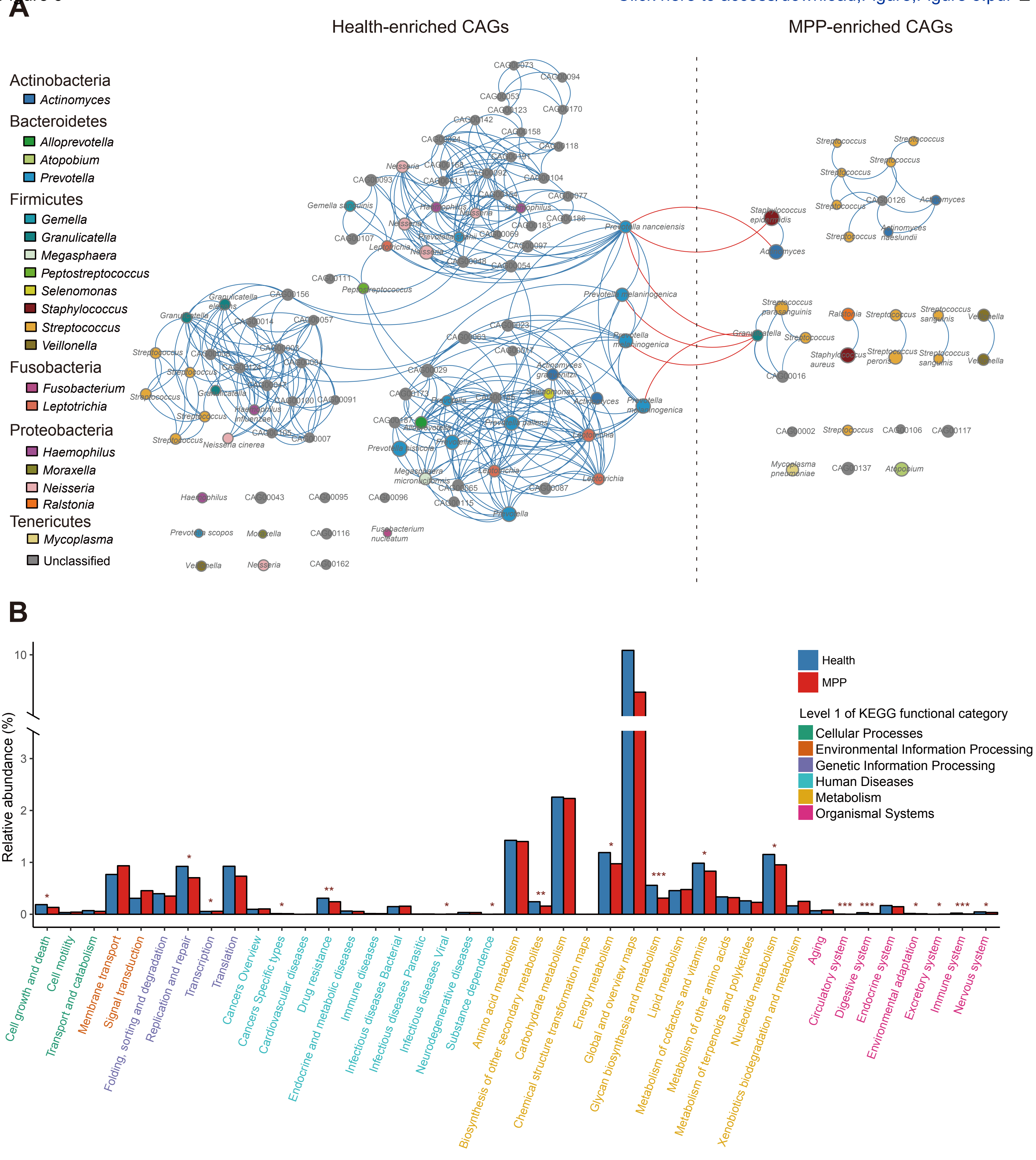

Figure 6

Click here to access/download;Figure;Figure 6.pdf

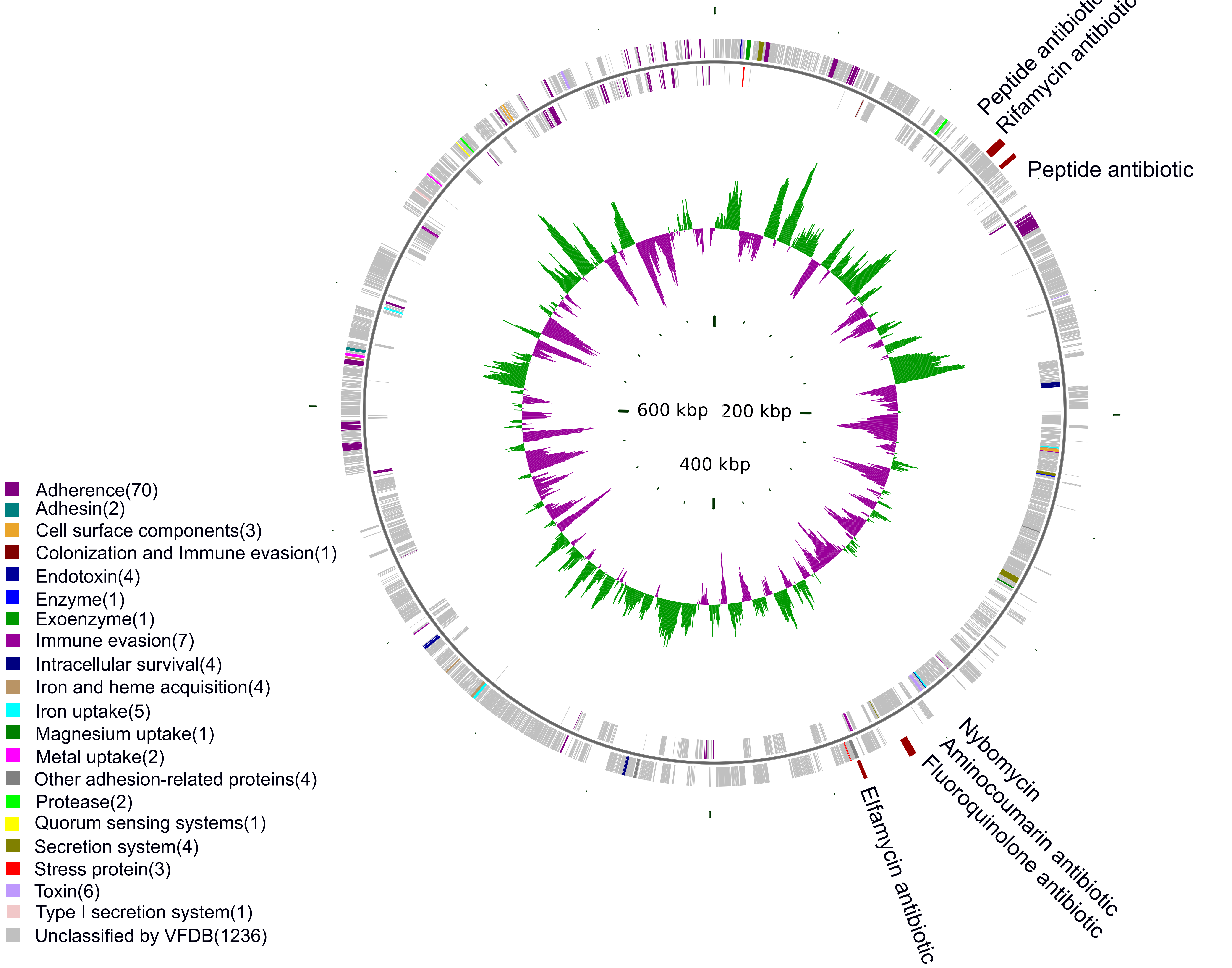

Figure 7

Click here to access/download;Figure;Figure 7.pdf

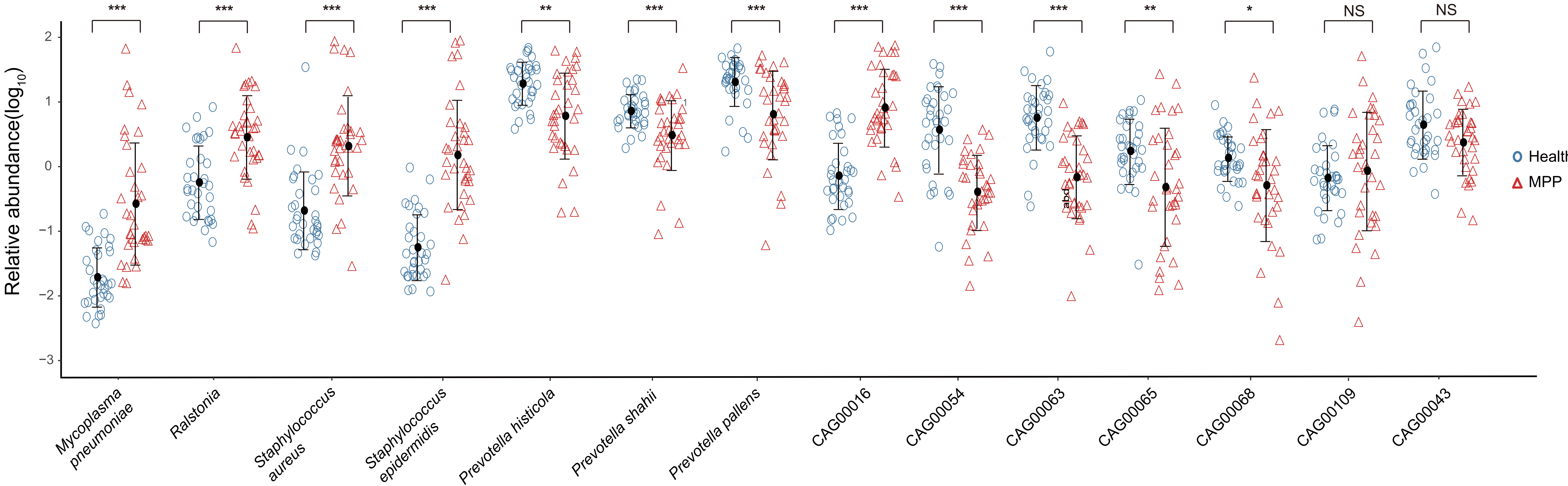

Supplemental Material Legends

Click here to access/download
Supplementary Material
Supplemental material legends.docx

Supplementary Figure 1

Click here to access/download
Supplementary Material
Supplementary Figure 1.pdf

Supplementary Figure 2

Click here to access/download
Supplementary Material
Supplementary Figure 2.pdf

Supplementary Figure 3

Click here to access/download
Supplementary Material
Supplementary Figure 3.pdf

Supplementary Table 1

Click here to access/download
Supplementary Material
Supplementary Table 1.xlsx

Supplementary Table 2

Click here to access/download
Supplementary Material
Supplementary Table 2.xlsx

Supplementary Table 3

Click here to access/download
Supplementary Material
Supplementary Table 3.xlsx

Supplementary Table 4

Click here to access/download
Supplementary Material
Supplementary Table 4.xlsx

Supplementary Table 5

Click here to access/download
Supplementary Material
Supplementary Table 5.xlsx

Supplementary Table 6

Click here to access/download
Supplementary Material
Supplementary Table 6.xlsx
