# Supplementary material for: An integrated respiratory microbial gene catalogue to better understand the microbial aetiology of Mycoplasma pneumoniae pneumonia
Source: Gigascience. 2019 Jul 31;8(8):giz093. doi: 10.1093/gigascience/giz093 (PMC6669060; doi:10.1093/gigascience/giz093)
Supplement: giz093_Supplemental_Files [file giz093_supplemental_files.zip › Supplemental material legends.docx]

**Supplementary materials**

**Supplementary Figure 1.** DNA gel electrophoresis results. M1: Marker 1(Trans 2k plus); M2: Marker 2(Trans 15k plus). The lightest bands are highlighted with red fonts. S: Human DNA, as standard sample. 1, 2, 3: Unused nasopharyngeal swabs; 4, 5, 6: Unused oropharyngeal swabs; 7, 8, 9: Enveloped DNA extraction kits.

**Supplementary Figure 2.** Comparison between taxonomic annotation by aligning metagenomics data with RMGC and 16S rRNA analysis. The number on the left of horizontal histogram represents sample ID. Upper histograms mean top 20 genera in 16S rRNA analysis results, and lower histograms represent microbial structure based on metagenomics analysis.

**Supplementary Figure 3.** Estimation of sequencing data abundance after filtering human sequence**.** The curve sharply decreased as the data size became less than 650 Mbp. X-axis means samples sorted by the usable sequencing data with descending order; Y-axis means the data size of sample.

**Supplementary Table 1.** Sample characteristics.

**Supplementary Table 2**. Enriched gene function (based on KEGG) between healthy children and patients with *Mycoplasma pneumoniae* pneumonia.

**Supplementary Table 3**. Evaluation of 14 assembled genomes derived from CAGs.

**Supplementary Table 4.** Antibiotic-resistance genes (ARGs) annotation in 14 re-assembled genomes

**Supplementary Table 5**. Virulence-factor genes (VFGs) annotation in 14 re-assembled genomes

**Supplementary Table 6**. Correlation between 14 genomes and clinical characters
