# Supplementary figures and images for: An integrated respiratory microbial gene catalogue to better understand the microbial aetiology of Mycoplasma pneumoniae pneumonia
Source: Gigascience. 2019 Jul 31;8(8):giz093. doi: 10.1093/gigascience/giz093 (PMC6669060; doi:10.1093/gigascience/giz093)

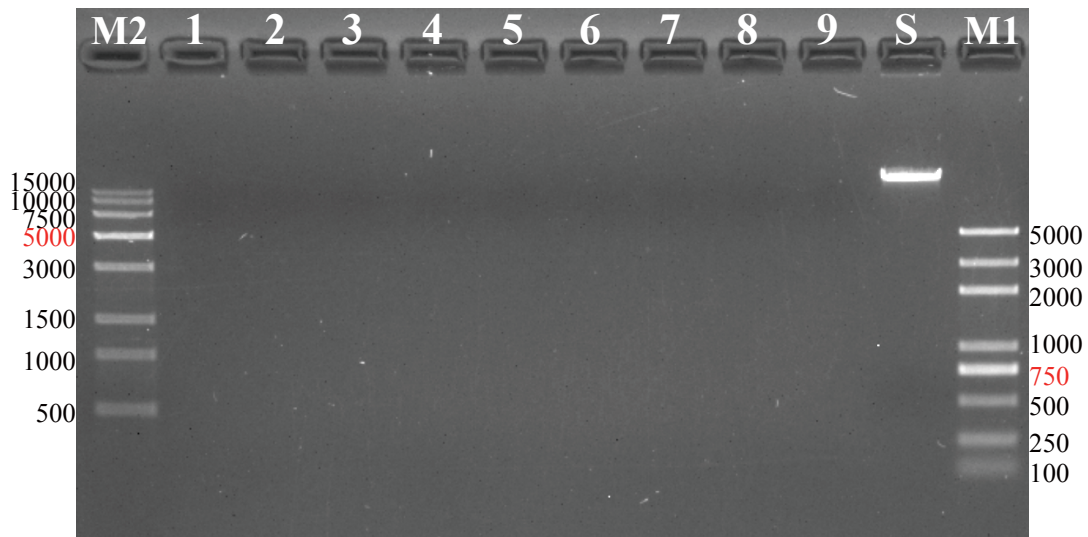

Supplement: giz093_Supplemental_Files [file giz093_supplemental_files.zip › Supplementary Figure 1.pdf]

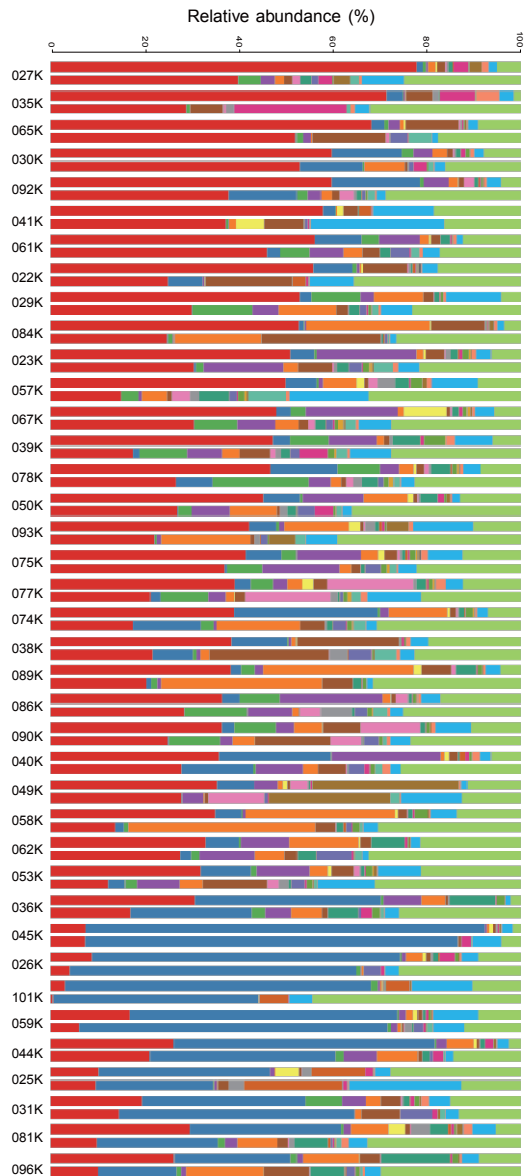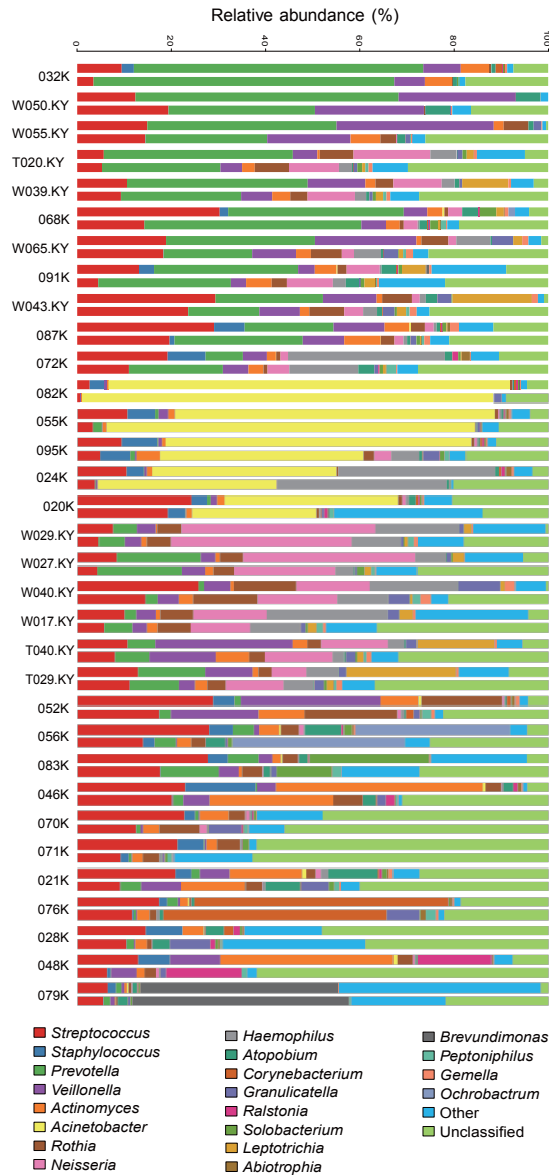

Supplement: giz093_Supplemental_Files [file giz093_supplemental_files.zip › Supplementary Figure 2.pdf]

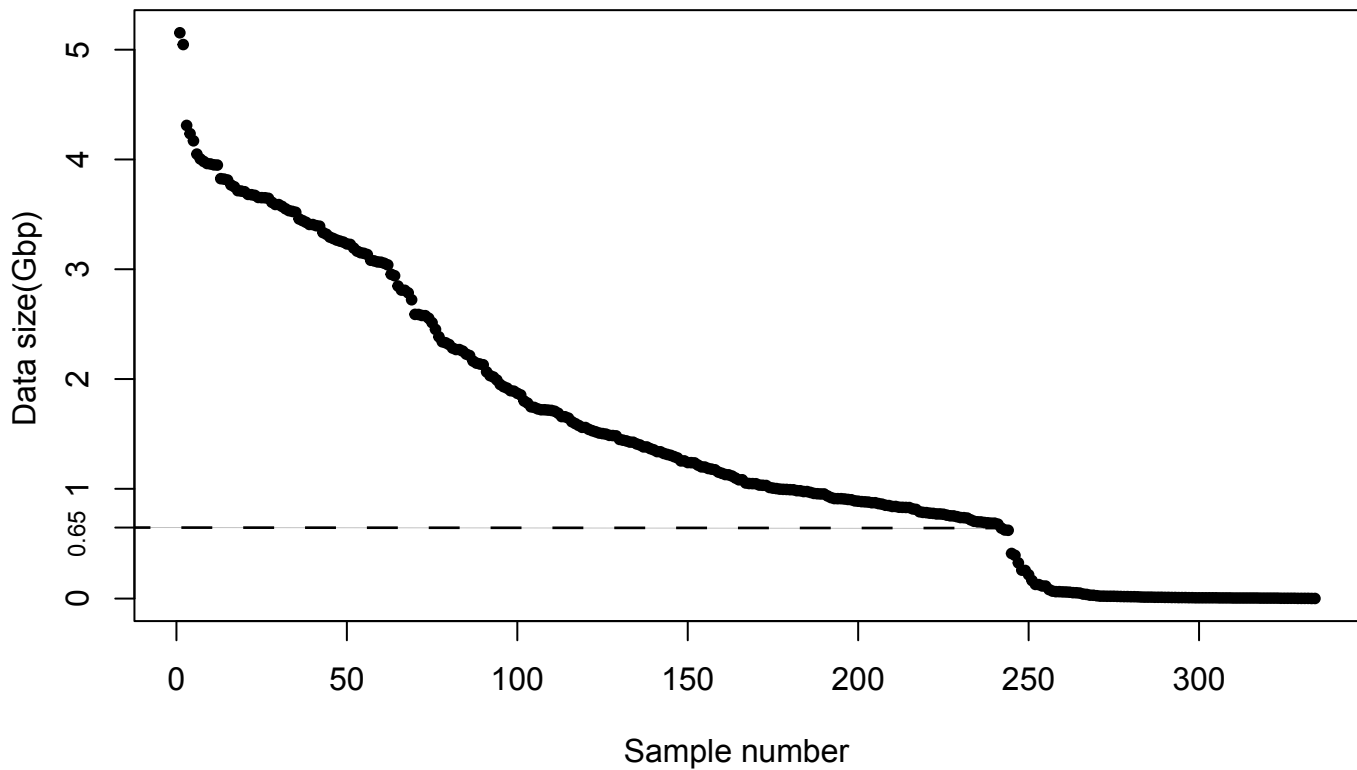

Supplement: giz093_Supplemental_Files [file giz093_supplemental_files.zip › Supplementary Figure 3.pdf]
