# Supplementary material for: An integrated respiratory microbial gene catalogue to better understand the microbial aetiology of Mycoplasma pneumoniae pneumonia
Source: Gigascience. 2019 Jul 31;8(8):giz093. doi: 10.1093/gigascience/giz093 (PMC6669060; doi:10.1093/gigascience/giz093)
Supplement: giz093_Response_to_Reviewer_Comments_Original_Submission [file giz093_response_to_reviewer_comments_original_submission.pdf]

Author's Response To Reviewer Comments

Close

Dear Editors and Reviewers,
Many thanks for your professional comments, please kindly check the responses below. As for the author list, we did adjustment based on manuscript revision, please kindly check the updated list in the manuscript.
Reviewer #1:
The authors constructed a respiratory microbial gene catalogue (RMGC) by metagenomics analysis on respiratory microbial samples from 171 healthy and 76 pneumonia children and compared the difference of gene set and microbiome between the healthy and pneumonia children. Data showed obvious difference between the two groups. I have some questions below:
1. Patient inclusion. This study focused on the etiology of Mycoplasma pneumoniae pneumonia (MPP), however, the enrollment criteria for the 76 patients was based on pneumonia and no specific requirement for Mpn positive test. Actually, there were only 52 patients listed in Supplemental Table 1 were Mpn positive in BALF. Then how did the comparison performed in the subsequent analysis? Were all 76 patients included or only the 52 Mpn-positive patients considered?
1. Response: Thanks for your questions. We aimed to construct a non-redundant integrated gene catalogue of respiratory microbes and applied the gene set to deepen the understanding of imbalanced microbial components as well as functions in Mycoplasma pneumoniae-associated pneumonia. Given different respiratory microbiota imbalance when infected by bacterial, Mycoplasma or viral pathogens, we included pneumonia children with infections of different pathogens and public respiratory microbial genomes to construct the integrated gene set. Then we focused on the imbalanced respiratory microbiome analysis for 34 patients, who were diagnosed as M. pneumoniae infection by BALF detection, compared to randomly selected 33 age-matched healthy children.

2. Line 146. The website is not accessible.
2. Response: We have updated the website of the RMGC and the URL also been changed (https://rmgc.deepomics.org), please kindly check the new URL link.

3. Line 169. Spell out "KOs" since it appeared first here in the text, not in the Methods part. Same as "CAGs" in line 193.
3. Response: Thanks for your kind reminding. The full names of aberrations (KOs and CAGs) were mentioned in the first appearance. KOs, KEGG Orthology; CAGs, Co-Abundance Gene Groups.

4. Line 72-73, 205-206 and 267-269. In the abstract, the authors stated "orthologues, which are associated with the metabolism of various lipids, membrane transport and signal transduction, accumulated significantly in the OP microbiome of sick children". However, in the text and Figure 5b, the enrichment is "slightly". In the discussion, the authors stated these gene functions could partly explain the reduced tight junction proteins and increased respiratory mucosa permeability after infection. Please discuss with more details and supporting references.
4. Response: Thanks for your careful review. We have checked the analysis results, and removed the inaccurate word “significantly” in the abstract. We also rewrote the related description in the manuscript. Please kindly check the revised manuscript.

5. Line 220-221. Please list the names of the 4 antibiotic-resistance genes.
5. Response: Thanks for your comment. We totally identified 4 homological antibiotic resistance genes via aligning the assembled genome to CARD (The Comprehensive Antibiotic Resistance Database), named “Mycobacterium tuberculosis gyrA conferring resistance to fluoroquinolones”, “Staphylococcus aureus rpoC conferring resistance to daptomycin”, “Staphylococcus aureus rpoB mutants conferring resistance to rifampicin” and “Escherichia coli EF-Tu mutants conferring resistance to kirromycin”. These gene names were also mentioned in Supplementary Table 4, please kindly check the revision.

6. Line 224-228. Please explain why the there are multiple copies of CARDS toxin genes (MPN372 in M129 genome) in the virulence-factor genes list. In all published Mpn genomes, CARDS toxin is a single copy gene. P1 adhesin is also a single copy gene, although it contains a repetitive element (repMp2/3)

which other copies are spread across the genome. Could there be any annotation errors for the assembled Mpn genome?
6. Response: Thanks for your professional comments. We have reassessed the genome assembly through the published international standard developed by the Genomic Standards Consortium (GSC), and the criterion of Mpn genome is "Medium-quality draft". Though the assembled genome size of Mycoplasma pneumonia (0.80Mb) is close to the reference genome(0.82Mb), 49 genomic fragments were not assembled. Due to the limitations of respiratory metagenomic sequencing, such as low bacterial biomass in airway samples, short paired-end reads and assembly methods, the genome sequences would be separated by homologous or repetitive sequences, and multiple gene segments could be found in different contigs. We have also rechecked the gene prediction results, it showed that multiple copies of CARDS toxin genes and P1 adhesion genes existed in different contigs. This limitation was also listed in the discussion, please kindly check the revision.

7. Line 292. "no macrolide/beta-lactam resistance genes were identified in M. pneumoniae genome". Macrolide resistance in Mpn is due to the point mutations in 23S rRNA gene. I believe 23S rRNA gene must present in the assembled Mpn genome. Please check if there are any mutations conferring macrolide resistance in 23S rRNA gene.
7. Response: Thanks for your professional suggestion. The RMGC was composed of coding sequences(CDS) gene rather than non-CDS gene (rRNA, tRNA). As you suggested, the sequencing data of M. pneumoniae-positive samples were further mapped against M. pneumoniae 23S rRNA gene, and we analyzed the samples with read-mapping coverage on the 23S rRNA more than 10x depths. We finally verified that 8 samples contained reliable 23S rRNA SNP mutations (A2063G), which was correlated to macrolide resistance. We have rewritten the manuscript about this SNP description. Please kindly check the new submission.

Reviewer #2:
This is an observational metagenomic/microbiome study that examines differences in taxonomic composition and gene content between children with pneumonia and controls. It compiles a rather large sample dataset, and the authors make available a gene catalog specific to the upper respiratory tract. It seems that the respiratory microbiome was well sampled, as evidenced by the rarefaction results.
1. I think the authors need to discuss their results in light of findings from other respiratory microbiome studies (which they partially do), and more importantly, of other gene catalog efforts such as:

Qin, J., Li, R., Raes, J., Arumugam, M., Burgdorf, K.S., Manichanh, C., Nielsen, T., Pons, N., Levenez, F., Yamada, T. and Mende, D.R., 2010. A human gut microbial gene catalogue established by metagenomic sequencing. nature, 464(7285), p.59.

Li, J., Jia, H., Cai, X., Zhong, H., Feng, Q., Sunagawa, S., Arumugam, M., Kultima, J.R., Prifti, E., Nielsen, T. and Juncker, A.S., 2014. An integrated catalog of reference genes in the human gut microbiome. Nature biotechnology, 32(8), p.834.

1. Response: Thanks for your suggestion. Refer to your suggested publications, we have rewritten the discussion about the contribution and potential influence of integrated respiratory microbial gene catalogue to respiratory microbiome study. Please kindly check the revision.

General Comments
2. Is it OK to compare NP, OP, and lung microbiota from pneumonia patients with OP from controls? How do you control for site specific microbiota composition?
2. Response: Thanks for your comment. We combined the data from NP, OP and lung microbiome in both diseased and healthy children to establish the respiratory microbial gene set. Given enough data depth in OP microbiota, we applied the reference gene set to analyze microbial components, genes and functions in each OP microbial sample, and then compared the OP microbiome differences between diseased and healthy children. We did not conduct comparison between NP, OP and lung microbiome in patients with OP microbiome in healthy children. This study design has been clarified in the revision, please kindly check it.

3. Did the authors control for the differences in gender and age? Those seem to be significant as judging by the proportion of females 31% and 50% for example. Did you perform any test to check whether these differences are significant?
3. Response: Thanks for your comments. We compared the OP microbiome differences between 34 pneumonia patients (16 girls and 18 boys) and 33 age-matched random-selected healthy children (15 girls and 18 boys) with data size more than 650 Mbp. There are no significant difference of age (p-value

=0.545, by t test) or gender (p-value=0.542, by Chi-square test) between two groups. We also checked other characteristics related to the microbial composition by PERMANOVA, and the “pneumonia onset” was the most significant factor (adjust p-value <0.001).

4. Assembly quality should be judge using published standards. The authors describe in detail how they obtained genomes from metagenomes. However, there are international standards regarding this, and it would be preferable that they use them so as to provide the readership with an objective point of comparison. See https://www.ncbi.nlm.nih.gov/pubmed/28787424
4. Response: Really appreciated your professional suggestions. We firstly assessed the assembly quality via 6 criteria at the “Single microbial genome assembling from OP metagenomic data” (referring to https://www.ncbi.nlm.nih.gov/pmc/articles/pmid/26425705/). As you suggested, we updated the assembly quality estimation following the published international standard developed by the Genomic Standards Consortium (GSC). The more comprehensive objective metrics of assembly quality were added in the Supplementary Table 3. Please kindly check the updated manuscript and Supplementary Table 3.

Minor Comments
5. Abstract
Line 62 - Define RMGC
Line 70 - What do you mean by "simple co-occurrence network"? What's so simple and what are complex co-occurrence networks
Line 74 - Please do not use apostrophes
5. Response:
Line 62: RMGC is the abbreviation for respiratory microbial gene catalogue, which comprised non-redundant respiratory microbial genes.
Line 70: Many thanks for your professional comments and we optimized the expressions in the revision.
Line 74: Thanks for your reminding and we removed it as you suggested.

6. Background
First paragraph - Please add something related to pneumonia worldwide. The authors say it's relevant in China but I'm sure it's relevant elsewhere as well.
Line 87 - Is it not the etiology of MPP Mycoplasma?
Line 89 - Please replace "the present" with "current"
Line 91 - There are plenty of pipelines that do not use OTU clustering and can get taxonomic classifications down to species. For example, see https://f1000research.com/articles/5-1492/v2
Line 93 - The authors mention that strain level microbiome studies are needed in MPP but they do not perform any strain-level analysis
6. Response:
First paragraph: Thanks for your suggestion. The worldwide medical information of pneumonia has been added in Background paragraph, please kindly check it.
Line 87: Mycoplasma pneumoniae is the pathogenic agent of MPP. However, it remained to be fully understood for differed disease severity in MPP patients. We aimed to deepen the understanding of microbial contributions in MPP incidence as well as progression, as a series of reports indicated the association of respiratory microbiota with respiratory disease severity.
Line 89: We have replaced the "the present" with "current".
Line 91: Many thanks for your professional suggestions, it is really a great tool for microbial amplicon analysis to get taxonomic classifications. We will refer to these pipelines to update previous respiratory tract microbiota studies. In this study, we conducted metagenome sequencing instead of 16S rRNA analysis to build a respiratory microbial gene catalogue as well as identify microbial functions and gene elements from assembled genomes.
Line 93: Really appreciated for your careful and professional comment. We intended to conduct species instead of strain-level analysis, and we revised it in the revision.

7. Analyses
Line 136 - It is better to express this in "read numbers" instead of Gbps.
Line 144 - Why do authors say bacterial genes? Did you filter any non-bacterial DNA sequences/reads? If you do shotgun sequencing you will likely find fungi, viruses and others. Please explain.
Line 146 - The webpage https://deepomics.org/respiratory_microbial_gene_catalogue/ does not exist
Line 148 - Could you elaborate on why using samples with more than 650 Mb? Why not 600 or 700?
Line 177 - Please replace "We totally acquired" by "We acquired 67 core species in total"
Line 193 - Please define CAGs as on this line the acronym appears for the first time
Line 217 - 2.30 Mb is very low for a bacterial genome. Please elaborate on why this might be. Comment

on genome completeness
Line 229 - What's the evidence regarding the designation of a new species? Nucleotide identity, gene content? Please discuss in the manuscript and consider some objective metric such as the Genome Taxonomy DB https://www.nature.com/articles/nbt.4229
Line 254 - Please revise this sentence as it is grammatically incorrect
7. Response:
Line 136: Thanks for your suggestion. We have replaced the “Gbps” by “read numbers”.
Line 144: We replaced this inadequate word “bacterial” by “microbial”.
Line 146: This website was updated recently, and moved to a new website (https://rmgc.deepomics.org). We declaimed that this website will be permanent. Please kindly check the revised manuscript.
Line 148: We have drawn the curve of sequencing data size (See Supplementary Figure 3), indicating sharp decrease after 0.65 Gb data size. In Method section, each sample with ≥650 Mbp data was selected for genome assembly, and the data of other samples were mixed for genome assembly. To better assess the RMGC, we then keep the consistent criterion (more than 650 Mb in Line 148).
Line 177: We modified the sentence as your suggestion, thanks for your kind comment.
Line 193: The full words of aberrations CAGs were Co-Abundance Gene Groups, please kindly check the revised manuscript.
Line 217: The average bacterial genome size ranged from 1Mb to 10Mb. The completeness of assembled microbial genomes was evaluated through the international standards (https://www.ncbi.nlm.nih.gov/pubmed/28787424), and the assembled genomes were classified as “Medium-quality draft genome”. Given the draft genome, several homologous or repetitive sequences would be removed in the assembly process. The detailed assessment of all 14 microbial genomes could refer to the update Table S3.
Line 229: Thanks for your suggestions. The species designation of 14 assembled genomes were following three evidences, including 1) concordance with taxonomic assignment of CAGs (https://www.nature.com/articles/nbt.2939); 2) aligned to the published genome sequences from IMG, NCBI and PATRIC via BLASTN (parameter: –e 0.01), with ≥95% nucleotide identity and ≥95% genome coverage; 3) assigned by CheckM from the Genome Taxonomy DB. Combined three evidences, the genome which could not be assigned to any known microbial species were defined as novel species. The detailed species designation method is added in the “Single microbial genome assembling from OP metagenomic data” paragraph of the Method section, and the updated taxonomy information could refer to Supplementary Table 3.
Line 254: We have revised the sentence as following: “The core microbial species of OP microbiota in healthy children will provide a reference for exploring microbial as well as host-microbe interactions in RM study.”

8. Please add the BioProject instead of the SRP accession
8. Response: We added the BioProject ID (PRJNA413615) in the revision, please kindly check it.

Close
