# Supplementary material for: An integrated respiratory microbial gene catalogue to better understand the microbial aetiology of Mycoplasma pneumoniae pneumonia
Source: Gigascience. 2019 Jul 31;8(8):giz093. doi: 10.1093/gigascience/giz093 (PMC6669060; doi:10.1093/gigascience/giz093)
Supplement: giz093_Response_to_Reviewer_Comments_Revision_1 [file giz093_response_to_reviewer_comments_revision_1.pdf]

Author's Response To Reviewer Comments

Close

Dear Editors and Reviewers,
Thanks for your comments. We have resubmitted the manuscript, please kindly check it.

1. Before we can proceed further you need to address the authorship issues we previously raised. i.e. that we only allow up to three joint-first authors and two corresponding authors. You can read more here:
https://doi.org/10.1093/gigascience/giy122
Please ensure all authors meet ICJME guidelines and have made an intellectual contribution to this paper, as if they haven't they should be moved to the acknowledgements:
http://www.icmje.org/recommendations/browse/roles-and-responsibilities/defining-the-role-of-authors-and-contributors.html
We require detailed authors contributions explaining all of this as well.

Response: We have resubmitted the revised manuscript without "Track Change" mode, and there are three joint-first authors and two corresponding authors in the revision. The authors who made an intellectual contribution were also mentioned in the "Authors' contributions" section. We hope this new submission could match your request.

Close
