# Supplementary material for: An integrated respiratory microbial gene catalogue to better understand the microbial aetiology of Mycoplasma pneumoniae pneumonia
Source: Gigascience. 2019 Jul 31;8(8):giz093. doi: 10.1093/gigascience/giz093 (PMC6669060; doi:10.1093/gigascience/giz093)
Supplement: giz093_Response_to_Reviewer_Comments_Revision_2 [file giz093_response_to_reviewer_comments_revision_2.pdf]

Author's Response To Reviewer Comments

Close

Dear Editors,

Thanks for your professional assessments and comments.
We have addressed the comments and approved the revision.
Please kindly check the updated manuscript without tracked changes.

Yuejie Zheng

Close
