# Supplementary material for: An integrated respiratory microbial gene catalogue to better understand the microbial aetiology of Mycoplasma pneumoniae pneumonia
Source: Gigascience. 2019 Jul 31;8(8):giz093. doi: 10.1093/gigascience/giz093 (PMC6669060; doi:10.1093/gigascience/giz093)
Supplement: giz093_Reviewer_1_Report_Original_Submission — Xiao Li -- 4/10/2019 Reviewed [file giz093_reviewer_1_report_original_submission.pdf]

Reviewer Report

Title: An integrated respiratory microbial gene catalogue to better understand the microbial aetiology of Mycoplasma pneumoniae pneumonia

Version: Original Submission **Date:** 4/10/2019

Reviewer name: Xiao Li

Reviewer Comments to Author:

The authors constructed a respiratory microbial gene catalogue (RMGC) by metagenomics analysis on respiratory microbial samples from 171 healthy and 76 pneumonia children and compared the difference of gene set and microbiome between the healthy and pneumonia children. Data showed obvious difference between the two groups. I have some questions below:

1. Patient inclusion. This study focused on the etiology of Mycoplasma pneumoniae pneumonia (MPP), however, the enrollment criterial for the 76 patients was based on pneumonia and no specific requirement for Mpn positive test. Actually, there were only 52 patients listed in Supplemental Table 1 were Mpn positive in BALF. Then how did the comparison performed in the subsequent analysis? Were all 76 patients included or only the 52 Mpn-positive patients considered?
2. Line 146. The website is not accessible.
3. Line 169. Spell out "KOs" since it appeared first here in the text, not in the Methods part. Same as "CAGs" in line 193.
4. Line 72-73, 205-206 and 267-269. In the abstract, the authors stated "orthologues, which are associated with the metabolism of various lipids, membrane transport and signal transduction, accumulated significantly in the OP microbiome of sick children". However, in the text and Figure 5b, the enrichment is "slightly". In the discussion, the authors stated these gene functions could partly explain the reduced tight junction proteins and increased respiratory mucosa permeability after infection. Please discuss with more details and supporting references.
5. Line 220-221. Please list the names of the 4 antibiotic-resistance genes.
6. Line 224-228. Please explain why the there are multiple copies of CARDS toxin genes (MPN372 in M129 genome) in the virulence-factor genes list. In all published Mpn genomes, CARDS toxin is a single copy gene. P1 adhesin is also a single copy gene, although it contains a repetitive element (repMp2/3) which other copies are spread across the genome. Could there be any annotation errors for the assembled Mpn genome?
7. Line 292. "no macrolide/beta-lactam resistance genes were identified in M. pneumoniae genome". Macrolide resistance in Mpn is due to the point mutations in 23S rRNA gene. I believe 23S rRNA gene must present in the assembled Mpn genome. Please check if there are any mutations conferring macrolide resistance in 23S rRNA gene.

Methods

Are the methods appropriate to the aims of the study, are they well described, and are necessary controls included? Choose an item.

Conclusions

Are the conclusions adequately supported by the data shown? Choose an item.

Reporting Standards

Does the manuscript adhere to the journal's guidelines on minimum standards of reporting? Choose an item.

Choose an item.

Statistics

Are you able to assess all statistics in the manuscript, including the appropriateness of statistical tests used? Choose an item.

Quality of Written English

Please indicate the quality of language in the manuscript: Choose an item.

Declaration of Competing Interests

Please complete a declaration of competing interests, considering the following questions:

- Have you in the past five years received reimbursements, fees, funding, or salary from an organisation that may in any way gain or lose financially from the publication of this manuscript, either now or in the future?
- Do you hold any stocks or shares in an organisation that may in any way gain or lose financially from the publication of this manuscript, either now or in the future?
- Do you hold or are you currently applying for any patents relating to the content of the manuscript?
- Have you received reimbursements, fees, funding, or salary from an organization that holds or has applied for patents relating to the content of the manuscript?
- Do you have any other financial competing interests?
- Do you have any non-financial competing interests in relation to this paper?

If you can answer no to all of the above, write 'I declare that I have no competing interests' below. If your reply is yes to any, please give details below.

I declare that I have no competing interests.

I agree to the open peer review policy of the journal. I understand that my name will be included on my report to the authors and, if the manuscript is accepted for publication, my named report including any attachments I upload will be posted on the website along with the authors' responses. I agree for my

report to be made available under an Open Access Creative Commons CC-BY license (http://creativecommons.org/licenses/by/4.0/). I understand that any comments which I do not wish to be included in my named report can be included as confidential comments to the editors, which will not be published.

I agree to the open peer review policy of the journal

To further support our reviewers, we have joined with Publons, where you can gain additional credit to further highlight your hard work (see: https://publons.com/journal/530/gigascience). On publication of this paper, your review will be automatically added to Publons, you can then choose whether or not to claim your Publons credit. I understand this statement.

Yes Choose an item.
