# Supplementary material for: An integrated respiratory microbial gene catalogue to better understand the microbial aetiology of Mycoplasma pneumoniae pneumonia
Source: Gigascience. 2019 Jul 31;8(8):giz093. doi: 10.1093/gigascience/giz093 (PMC6669060; doi:10.1093/gigascience/giz093)
Supplement: giz093_Reviewer_2_Report_Original_Submission — Eduardo Castro -- 4/20/2019 Reviewed [file giz093_reviewer_2_report_original_submission.pdf]

**Reviewer Report**

**Title: An integrated respiratory microbial gene catalogue to better understand the microbial aetiology of Mycoplasma pneumoniae pneumonia**

**Version: Original Submission** **Date:** 4/20/2019

**Reviewer name: Eduardo Castro**

**Reviewer Comments to Author:**

This is an observational metagenomic/microbiome study that examines differences in taxonomic composition and gene content between children with pneumonia and controls. It compiles a rather large sample dataset, and the authors make available a gene catalog specific to the upper respiratory tract. It seems that the respiratory microbiome was well sampled, as evidenced by the rarefaction results.

I think the authors need to discuss their results in light of findings from other respiratory microbiome studies (which they partially do), and more importantly, of other gene catalog efforts such as:

Qin, J., Li, R., Raes, J., Arumugam, M., Burgdorf, K.S., Manichanh, C., Nielsen, T., Pons, N., Levenez, F., Yamada, T. and Mende, D.R., 2010. A human gut microbial gene catalogue established by metagenomic sequencing. nature, 464(7285), p.59.

Li, J., Jia, H., Cai, X., Zhong, H., Feng, Q., Sunagawa, S., Arumugam, M., Kultima, J.R., Prifti, E., Nielsen, T. and Juncker, A.S., 2014. An integrated catalog of reference genes in the human gut microbiome. Nature biotechnology, 32(8), p.834.

General Comments

Is it OK to compare NP, OP, and lung microbiota from pneumonia patients with OP from controls? How do you control for site specific microbiota composition?

Did the authors control for the differences in gender and age? Those seem to be significant as judging by the proportion of females 31% and 50% for example. Did you perform any test to check whether these differences are significant?

Assembly quality should be judge using published standards. The authors describe in detail how they obtained genomes from metagenomes. However, there are international standards regarding this, and it would be preferable that they use them so as to provide the readership with an objective point of comparison. See https://www.ncbi.nlm.nih.gov/pubmed/28787424

Minor Comments

Abstract

Line 62 - Define RMGC

Line 70 - What do you mean by "simple co-occurrence network"? What's so simple and what are complex co-occurrence networks

Line 74 - Please do not use apostrophes

Background

First paragraph - Please add something related to pneumonia worldwide. The authors say it's relevant in China but I'm sure it's relevant elsewhere as well.

Line 87 - Is it not the etiology of MPP Mycoplasma?
Line 89 - Please replace "the present" with "current"
Line 91 - There are plenty of pipelines that do not use OTU clustering and can get taxonomic classifications down to species. For example, see https://f1000research.com/articles/5-1492/v2
Line 93 - The authors mention that strain level microbiome studies are needed in MPP but they do not perform any strain-level analysis
Analyses
Line 136 - It is better to express this in "read numbers" instead of Gbps.
Line 144 - Why do authors say bacterial genes? Did you filter any non-bacterial DNA sequences/reads? If you do shotgun sequencing you will likely find fungi, viruses and others. Please explain.
Line 146 - The webpage https://deepomics.org/respiratory_microbial_gene_catalogue/ does not exist
Line 148 - Could you elaborate on why using samples with more than 650 Mb? Why not 600 or 700?
Line 177 - Please replace "We totally acquired" by "We acquired 67 core species in total"
Line 193 - Please define CAGs as on this line the acronym appears for the first time
Line 217 - 2.30 Mb is very low for a bacterial genome. Please elaborate on why this might be. Comment on genome completeness
Line 229 - What's the evidence regarding the designation of a new species? Nucleotide identity, gene content? Please discuss in the manuscript and consider some objective metric such as the Genome Taxonomy DB https://www.nature.com/articles/nbt.4229
Line 254 - Please revise this sentence as it is grammatically incorrect
Please add the BioProject instead of the SRP accession

**Methods**

Are the methods appropriate to the aims of the study, are they well described, and are necessary controls included? Choose an item.

**Conclusions**

Are the conclusions adequately supported by the data shown? Choose an item.

**Reporting Standards**

Does the manuscript adhere to the journal's guidelines on minimum standards of reporting? Choose an item.

Choose an item.

**Statistics**

Are you able to assess all statistics in the manuscript, including the appropriateness of statistical tests used? Choose an item.

**Quality of Written English**

Please indicate the quality of language in the manuscript: Choose an item.

**Declaration of Competing Interests**

Please complete a declaration of competing interests, considering the following questions:

- Have you in the past five years received reimbursements, fees, funding, or salary from an organisation that may in any way gain or lose financially from the publication of this manuscript, either now or in the future?
- Do you hold any stocks or shares in an organisation that may in any way gain or lose financially from the publication of this manuscript, either now or in the future?
- Do you hold or are you currently applying for any patents relating to the content of the manuscript?
- Have you received reimbursements, fees, funding, or salary from an organization that holds or has applied for patents relating to the content of the manuscript?
- Do you have any other financial competing interests?
- Do you have any non-financial competing interests in relation to this paper?

If you can answer no to all of the above, write 'I declare that I have no competing interests' below. If your reply is yes to any, please give details below.

I declare that I have no competing interests

I agree to the open peer review policy of the journal. I understand that my name will be included on my report to the authors and, if the manuscript is accepted for publication, my named report including any attachments I upload will be posted on the website along with the authors' responses. I agree for my report to be made available under an Open Access Creative Commons CC-BY license (http://creativecommons.org/licenses/by/4.0/). I understand that any comments which I do not wish to be included in my named report can be included as confidential comments to the editors, which will not be published.

I agree to the open peer review policy of the journal

To further support our reviewers, we have joined with Publons, where you can gain additional credit to further highlight your hard work (see: https://publons.com/journal/530/gigascience). On publication of this paper, your review will be automatically added to Publons, you can then choose whether or not to claim your Publons credit. I understand this statement.

Yes Choose an item.
